# FoldAffinity: binding affinities from nDSF experiments

**DOI:** 10.1038/s41598-021-88985-z

**Published:** 2021-05-05

**Authors:** Stephan Niebling, Osvaldo Burastero, Jérôme Bürgi, Christian Günther, Lucas A. Defelipe, Simon Sander, Ellen Gattkowski, Raghavendra Anjanappa, Matthias Wilmanns, Sebastian Springer, Henning Tidow, María García-Alai

**Affiliations:** 1 European Molecular Biology Laboratory - Hamburg Outstation, Notkestr. 85, Hamburg, 22607 Germany; 2 Hamburg Advanced Research Centre for Bioorganic Chemistry (HARBOR) & Department of Chemistry, Institute for Biochemistry and Molecular Biology, University of Hamburg, Luruper Chaussee 149, 22761 Hamburg, Germany; 3 Department of Life Sciences and Chemistry, Jacobs University Bremen, 28759 Bremen, Germany; 4 Centre for Structural Systems Biology, Notkestrasse 85, 22607 Hamburg, Germany; 5 University Hamburg Clinical Centre Hamburg-Eppendorf, Martinistraße 52, 20246 Hamburg, Germany; 6 Departamento de Química Biológica, Facultad de Ciencias Exactas y Naturales, Universidad de Buenos Aires, Intendente Güiraldes 2620, Buenos Aires, Argentina; 7 IQUIBICEN-UBA/CONICET, Intendente Güiraldes, 2620 Buenos Aires, Argentina

**Keywords:** Biochemistry, Biophysics, Chemical biology, Drug discovery

## Abstract

Differential scanning fluorimetry (DSF) using the inherent fluorescence of proteins (nDSF) is a popular technique to evaluate thermal protein stability in different conditions (e.g. buffer, pH). In many cases, ligand binding increases thermal stability of a protein and often this can be detected as a clear shift in nDSF experiments. Here, we evaluate binding affinity quantification based on thermal shifts. We present four protein systems with different binding affinity ligands, ranging from nM to high μM. Our study suggests that binding affinities determined by isothermal analysis are in better agreement with those from established biophysical techniques (ITC and MST) compared to apparent *K*_*d*_s obtained from melting temperatures. In addition, we describe a method to optionally fit the heat capacity change upon unfolding ($\Delta {C}_{p}$) during the isothermal analysis. This publication includes the release of a web server for easy and accessible application of isothermal analysis to nDSF data.

## Introduction

One protein binds a few molecules out of the thousands of different types it encounters in a cell and the properties of those physical interactions are key determinants in biological processes. There are well-established in vitro biophysical methods to address protein–ligand interactions and every technique has advantages and disadvantages for the study of a particular biological system^1^. Some of the established technologies to determine binding affinities are isothermal titration calorimetry (ITC), surface plasmon resonance (SPR), microscale thermophoresis (MST)^2,3^ and fluorescence-based assays among others^4,5^.

Particularly, differential scanning fluorimetry (DSF), also termed fluorescent thermal shift assay (FTSA), a methodology commonly applied to investigate the stability of proteins in different environments^6–9^, is often used to probe for the interaction between compounds and proteins in drug discovery and fragment screening^10–16^. Typically, the ligand binding affinity is presented by showing the ${T}_{m}$-shift as a function of ligand concentration^17^. However, quantifying the corresponding binding constant (${K}_{d}$) from the unfolding curves at varying ligand concentrations and temperatures retrieves only an approximated value known as “apparent ${K}_{d}$” that does not take into account the temperature dependence of the binding constant.

The isothermal approach instead uses the fraction of protein that is folded at a temperature range near the unfolding transition assuming a constant temperature, where protein folding/unfolding and the ligand binding equilibria are coupled^16^. However, often proteins do not unfold in a reversible two-state manner, and therefore the equilibrium thermodynamics models do not apply.

Here, we evaluate the isothermal strategy and improve the analysis of DSF data. We assess the isothermal approach with different biological model systems by comparing the binding affinities to the ones determined by either isothermal titration calorimetry (ITC) or microscale thermophoresis (MST). We also compare binding affinities determined by isothermal analysis to “apparent” binding affinities obtained by fitting a single-site model to the nDSF melting temperatures^17^. In this manuscript, we describe a method to optionally fit the heat capacity change upon unfolding ($\Delta {C}_{p}$) during the isothermal analysis and show that this approach provides more accurate results to assuming a value of zero for a set of simulated cases. The whole methodology is made available as a web server including the latter experimental feature.

## Results

### “Apparent” binding affinities from melting temperatures

A common approach to compare binding affinities of different ligands is the determination of a so-called “apparent ${K}_{D}$” by fitting a simple 1:1 binding model to the melting temperatures versus ligand concentrations^17^:

1 $${T}_{m}([L{]}_{0})={T}_{m, unbound}+{(T}_{m, upper}-{T}_{m, unbound})*(1-a([L{]}_{0}))$$

2 $$\mathrm{a}({[L]}_{0})=\left({\left[P\right]}_{0}-{K}_{d}-{\left[L\right]}_{0} +\sqrt{({\left[P\right]}_{0}+{\left[L\right]}_{0}+ {K}_{d})2-4{\left[P\right]}_{0}{\left[L\right]}_{0} )}\right)/ (2 * {[P]}_{0})$$

In this 0th order approximation, ${T}_{m}$ values are fitted directly versus a complexation ratio. It is important to stress that this approach has no physico-chemical foundation. In the following, this will be referred to as model 1. Since a ${K}_{d}$ is defined for a certain temperature, this approach is thermodynamically not correct. Furthermore, this model assumes the T_m_ to converge to a well-defined melting temperature T_m,upper_, which is incorrect^11,12,18^. Despite its shortcomings, its simplicity made this approach popular.

A thermodynamic-based approach is to use the following expression^18,19^:

3 $$\frac{{\Delta T}_{m}({\left[L\right]}_{0})}{{T}_{m}({\left[L\right]}_{0})}=\frac{{\Delta T}_{m}\left({\left[L\right]}_{0}\right)- {T}_{m0}}{{T}_{m}({\left[L\right]}_{0})}= \frac{R*{T}_{m0}}{{\Delta H}_{{T}_{m0}}}*\mathrm{ln}\left(1+\frac{{\left[L\right]}_{0}}{{K}_{d}}\right)$$

Here T_m_([L]_0_) is the melting temperature of the protein in presence of the initial ligand concentration [L]_0_. T_m0_ is the protein melting temperature in absence of the ligand. ΔH_Tm0_ is the unfolding enthalpy of the protein in absence of the ligand at T_m0_ and K_d_ the apparent dissociation constant. Rearranging this equation yields an expression for T_m_([L]_0_) that we have used to fit binding affinities directly from the observed melting temperatures:

4 $${T}_{m}\left({\left[L\right]}_{0}\right)= \frac{{T}_{m0}}{1- \frac{R*{T}_{m0}}{{\Delta H}_{{T}_{m0}}}*\mathrm{ln}\left(1+\frac{{\left[L\right]}_{0}}{{K}_{d}}\right)}$$

In the following this approach will be referred to as model 2. It is important to note that this model approximates the free ligand concentration [L] to be equal to the initial ligand concentration [L]_0_ which can lead to deviations for low ligand concentrations and/or high binding affinities (low K_d_).

### Isothermal approach and fitting of $\Delta {C}_{p}$ values

**Figure 1:**
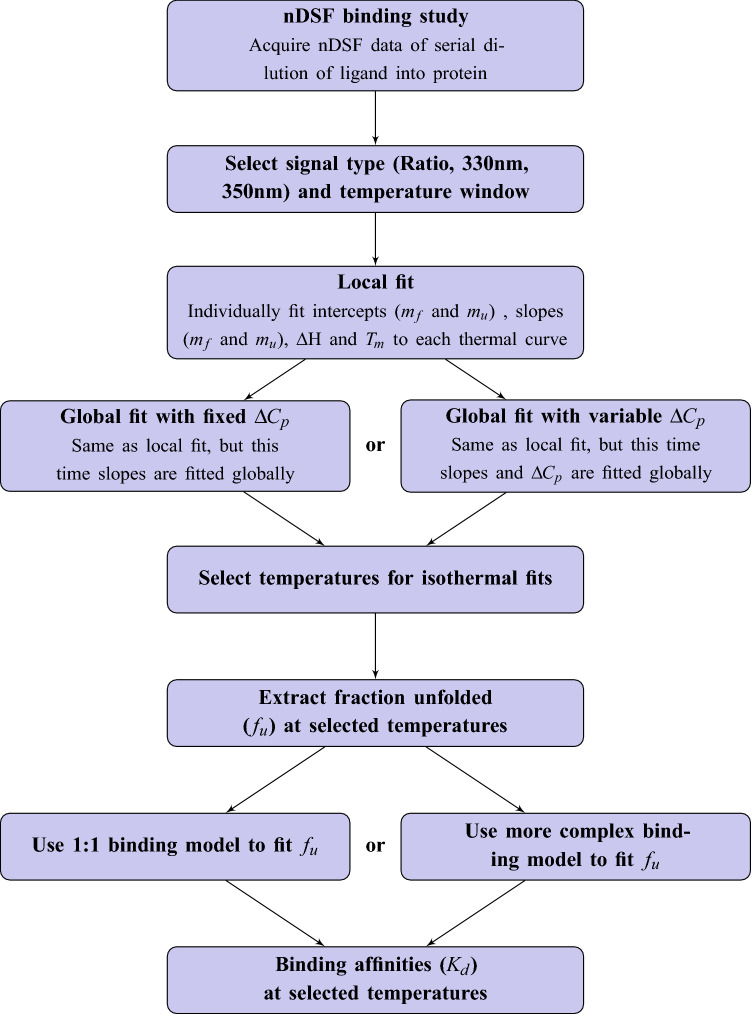
Flow chart of isothermal analysis of nDSF binding study. After selecting the signal type (*F*_330_, *F*_350_ or Ratio) and the spectral window, a local fit of the thermal curves yields starting values for the subsequent global fit. This can be either done with a fixed ∆*C*_*p*_ or with ∆*C*_*p*_ as fitted variable. The latter option is still an experimental feature. The next step is the calculation of the fraction unfolded *f*_*u*_ for selected temperatures. Fitting these *f*_*u*_ with a suitable binding model then yields a binding affinity for each selected temperature. A detailed description of the isothermal fitting routine can be found in Supplementary Information S10.

Similar to model 2 [Eq. (4)], isothermal analysis of differential scanning fluorimetry (DSF) data is based on a thermodynamic model^16^. However, the K_d_ is determined at a chosen fixed temperature by fitting the fraction unfolded versus the initial ligand concentration [L]_0_, instead of fitting the melting temperature T_m_ (i.e. at a constant fraction unfolded f_u_ = 0.5) versus the initial ligand concentration [L]_0_ as assumed in model 2. The isothermal method involves several steps that are shown in Fig. 1. In all the examples shown here, we use a reversible 2-state unfolding model between folded ($F$) and unfolded protein ($U$) and a 1:1 binding model for the complex ($FL$) formation. Ligand binding of the unfolded species is not taken into account. Combining the two equilibria results in the following equation:

5 $$ {\text{FL}}\overset {K_{{\text{d}}} } \rightleftharpoons {\text{F}} + {\text{L}}\overset {K_{{\text{U}}} } \rightleftharpoons {\text{U}} + {\text{L}} $$

where ${K}_{D}$ is the dissociation constant and ${K}_{U}$ the unfolding equilibrium constant:

6 $${K}_{d}=\frac{[F][L]}{[FL]}$$

7 $${K}_{U}=\frac{[U]}{[F]}$$

where squared brackets denote equilibrium concentrations. During the analysis, it is assumed that DSF can only distinguish between folded (sum of $F$ and $FL$) and unfolded species ($U$). The fraction unfolded is a central quantity for the determination of a binding constant or for the simulation of fluorescence melting curves specified in the following section (cf. Equation 14). It is the concentration ratio of unfolded protein and the total protein [P]_0_.

8 $${f}_{u}=\frac{[U]}{[P{]}_{0}}=\frac{ [U]}{[U]+[F]+[FL]}$$

One obstacle in the application of isothermal analysis so far is the necessity to know the change of heat capacity upon unfolding $\Delta {C}_{p}$ prior to the analysis. This quantity is needed for fitting the DSF melting curves and influences the Gibbs energy of unfolding $\Delta {G}_{U}$. At temperatures $T$ close to the melting temperature ${T}_{m}$, $\Delta {G}_{U}$ (in absence of ligand) can be expressed as:

9 $$\Delta {G}_{U}(T)=\Delta {H}_{{T}_{m}}+\Delta {C}_{P}(T-{T}_{m})-T(\Delta {S}_{{T}_{m}}+\Delta {C}_{P} ln(\frac{T}{{T}_{m}}) )$$

$\Delta {C}_{p}$ can experimentally be determined by differential scanning calorimetry (DSC). This method can be sample consuming and challenging as far as data analysis is concerned. Thus it is common practice to assume a $\Delta {C}_{p}$ of zero when it comes to fitting differential scanning fluorimetry curves. It would be desirable to obtain the $\Delta {C}_{p}$ “on-the-fly” during the isothermal analysis approach without the need for a priori knowledge or additional experiments. Therefore, we have complemented the common approach to specify a $\Delta {C}_{p}$ with a global fit that optimizes this quantity as a variable during the fitting process of the thermal unfolding curves (cf. Fig. 1). It is important to note, that this approach so far is an experimental feature and still needs to be carefully assessed case-to-case. To further scrutinize isothermal analysis and in particular the fitting of $\Delta {C}_{p}$ presented here, we will present extensive simulations in the next section.

### Simulations

To assess the robustness of the presented method including the fitting of $\Delta {C}_{p}$ values, data were simulated with different combinations of binding affinities ${K}_{D}$ ranging from ${10}^{-10}$ M to ${10}^{-1}$ M, protein concentrations ranging from ${10}^{-10}$ M to ${10}^{-1}$ M and heat capacity changes upon unfolding ranging from 0 to 12 kcal/mol K (0, 4, 8 and 12 kcal/mol K) using the following equations. The equations shown here are based on previous studies by Matulis et al*.*^11^ and Bai et al*.*^16^. The steps for preparing the data are:

1. Generate the temperature dependent Gibbs energy of unfolding ${G}_{U}\left(T\right)$ with a melting temperature in the absence of ligand ${T}_{m}$, the unfolding enthalpy (at ${T}_{m}$) $\Delta {H}_{U,{T}_{m}}$ and the change in heat capacity $\Delta {C}_{p,U}$ using the Eq. (9).
2. The total Gibbs energy $\Delta G$ is a sum of a term for unfolding $\Delta {G}_{U}\left(T\right)$ and a term for dissociation of the ligand $\Delta {G}_{d}$^11^. The dissociation constant ${K}_{d}$ for the simulated data is assumed to be temperature independent for simplicity. This needs to be done for each initial ligand concentration [L]_0_.
  10 $$\Delta {G}_{U}(T,[L{]}_{0})=\Delta {G}_{U}(T)+RT*ln (1+\frac{[L{]}_{Free}}{{K}_{d}})$$
  Here, R is the gas constant in kcal/mol and [L]free is the unbound ligand concentration that can be calculated with the following equation for each.
  11 $$[L]_{free}=\frac{ [L{]}_{0}-[P{]}_{0}/({K}_{u}+1) -{K}_{d} }{2} +\sqrt{\frac{{([L{]}_{0}+[P{]}_{0}/({K}_{u}+1)+{K}_{d})}^{2}}{4}-\frac{[L{]}_{0}[P{]}_{0}}{{K}_{U}+1}}$$
3. Calculate the apparent unfolding equilibrium constant ${K}_{U}{^{\prime}}\left(T\right)$ using Eqs. (10) and (11) for each [L]_0_.
  12 $$K{^{\prime}}_{U}(T)={e}^{-\Delta G(T) / RT}$$
4. Calculate the fraction unfolded ${f}_{u}\left(T\right)$ (cf. Equation 8)
  13 $${f}_{u}\left(T\right)= \frac{K{^{\prime}}_{U}(T) }{K{^{\prime}}_{U}\left(T\right)+1}$$
5. *f*_u_(*T*) can then be used to model the fluorescence signal $Y\left(T\right)$
  14 $$Y(T)={f}_{u}(T)*({m}_{u}T+{b}_{u})+(1- {f}_{u}(T))*({m}_{f}T+{b}_{f})$$
  Here ${m}_{u}$, ${m}_{f}$ are the slopes of and ${b}_{u}$, ${b}_{f}$ the intercepts of the unfolded/folded state. For simplicity, the signal of the folded bound state is assumed to be equal to the signal of the folded unbound state.
6. Add noise ranging from 0 to 10% of the dynamic range of the data (0, 1, 2, 5 and 10%).

This yields in total 1500 virtual binding studies that were analyzed by the same approach as the real experimental data shown in this manuscript (cf. Fig. 1). To investigate the effect of $\Delta {C}_{p}$ fitting, we have done data analysis with and without this feature. The data was grouped according to *K*_*d*_, $\Delta {C}_{p}$, noise level and whether or not $\Delta {C}_{p}$ was used (each combination contains 100 virtual binding studies). For each of these groups the *K*_*d*_ error was determined by dividing the fitted *K*_*d*_ by the true *K*_*d*_ and color coded as shown in Fig. 2 for $\Delta {C}_{p}=8$ kcal/molK and a noise level of 2%, which is a realistic noise level observed for the experimental spectra shown in this work (cf. Fig. S1). All other results are shown in the supplementary information (Figs. S1–S23).

**Figure 2:**
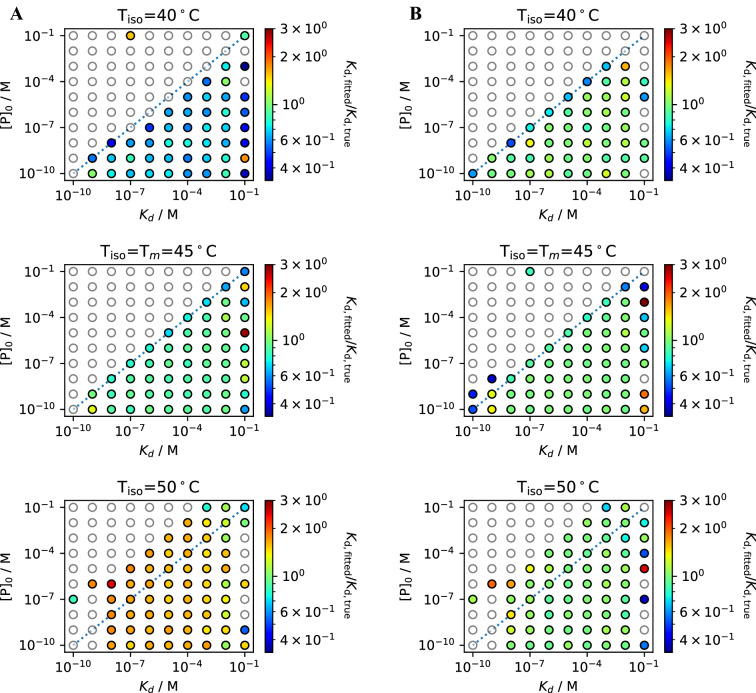
*K*_*d*_ fits for simulated data with ∆*C*_*p*_ = 8 kcal/mol K and 2% noise. *K*_*d*_ deviations $\left( {\frac{{K_{{\text{d,fitted}}} }}{{K_{{\text{d,true}}} }}} \right)$ are color coded with green corresponding to the initial value. Datesets outside the range are shown as white circles. Fitting the ∆*C*_*p*_ during the thermal curve fitting process results in better agreement with the initial *K*_*d*_ values (**B**) compared to fits with a fixed ∆*C*_*p*_ = 0 kcal/mol K (**A**). The fitted ∆*C*_*p*_ values are shown in Supplementary Information S22–S23.

In the investigated cases, the $\Delta {C}_{p}$ could be fitted accurately (Figs. S22/S23) as long as the noise level did not exceed an unusual threshold (ca. 5%). This held true even for high $\Delta {C}_{p}$ values, with the start value for the optimization being always 0. Even for a $\Delta {C}_{p}=12$ kcal/molK with a noise level of 2% the fitting could recover the $\Delta {C}_{p}=12$ kcal/molK accurately (Fig. S23). The simulations underline the recommendation for choosing a concentration smaller than the *K*_*d*_^20^, if experimentally possible, for an accurate *K*_*d*_ determination since the *K*_*d*_ deviations are in general higher above the diagonal (dashed line in Fig. 2). The lower limit of protein concentration is set by the fluorescence signal or the signal-to-noise ratio, which depends on the number of chromophores and their sensitivity to protein unfolding. If a $\Delta {C}_{p}$ of 0 is assumed with a true $\Delta {C}_{p}\ne 0,$ the lowest *K*_*d*_ deviations are obtained when the temperature chosen for isothermal analysis T_iso_ is close to the melting temperature T_m_.

### Proof of principle studies

In the following section, we will present nDSF binding studies with different protein systems and small molecule binding partners. For three systems, the binding affinities determined by isothermal analysis of nDSF data were compared to ITC or MST determined *K*_*d*_ values. In addition to that, we determined apparent binding affinities ${K}_{d,app}$ using model 1 [Eq. (1)] and model 2 [Eq. (4)], to compare these values to the *K*_*d*_ values determined by isothermal analysis. The results of these proof-of-principle studies are summarized in Table 2.

One open question is which $\Delta {C}_{p}$ value to use for isothermal analysis. Fitting the $\Delta {C}_{p}$ has proven to work in our simulations but is still an experimental feature until assessed with experimental values. In the supporting information we show the DSC data for Pcs60, one protein that was used to test isothermal analysis of nDSF data (Fig. S24). In this case, we were not able to determine the $\Delta {C}_{p}$ from a simple DSC experiment due to overlapping transitions. The first of these transitions corresponds to the unfolding observed in nDSF experiments. In the provided example, to obtain an experimental $\Delta {C}_{p}$ would require a measurement series with different pH or denaturant concentrations as the system does not show an ideal two-state unfolding equilibrium. Here we suggest that when $\Delta {C}_{p}$ cannot be determined experimentally, it can be approximated computationally from the surface accessible area changes upon unfolding^21,22^ or from the number of residues ${N}_{res}$ of the protein^21^:

15 $$\Delta {C}_{P}=13.88*{N}_{res }\frac{cal}{mol \,  K}$$

In Table 1, we compared the estimated $\Delta {C}_{p}$ values for the different systems with the fitted values. The fitted value were in each case closer to the estimated value than 0 kcal/mol K. Due to the lack of experimental $\Delta {C}_{p}$ values and the fact that the $\Delta {C}_{p}$ fitting is still an experimental feature that still needs to be scrutinized with experimental values, we have decided to present all isothermal analysis shown in the main text with a $\Delta {C}_{p}$ set to zero. The results are summarized in Table 2. As a comparison, we show all the analysis results with the fitted $\Delta {C}_{p}$ values listed in Table 1 in the supplementary information (Figs. S25–S28). Our results indicate that fitting $\Delta {C}_{p}$ provides more accurate *K*_*d*_s than assuming a value of zero.

**Table 1:** Estimated and fitted ∆C_p_ values for the systems presented in this work. The ∆C_p_ was estimated using Eq. (15).

Measurement	*N*_res_	$\Delta {\text{C}}_{{\text{p}}} /\frac{{{\text{kcal}}}}{{{\text{mol}}*{\text{K}}}}$	
		Estimated (*N*_res_)	Fitted
EG1/ADPR	350	4.8	8.8
SS1/ADPR	422	5.9	7.6
MHC/NT8	374	5.2	4.0
Pcs60/ATP	535	7.4	6.7

**Table 2:** Proof-of-principle studies of isothermal analysis of nDSF data.

System	Isothermal analysis		Melting temperature analysis			ITC/MST measurement		
	T_iso_	*K*_*d*_	*T*_*m*_/°C	*K*_d*,*app*,*model1_	*K*_d*,*app*,*model2_	T_ref_	*K*_*d*_	Ref.
EG1/ADPR	44 °C	442 nM ± 27%	51–56	172 μM ± 5%	14.6 μM ± 12%	20 °C	16 nM ± 25%	Figure 3
SS1/ADPR	50 °C	6.5 μM ± 3%	50–60	77 μM ± 6%	5.8 μM ± 8%	25 °C	3.5 μM	Figure 4
MHC/NT8	32 °C	6.3 μM ± 9%	29–41	45 μM ± 7%	14.8 μM ± 34%	N/A	N/A	Figure 5
Pcs60/γS-ATP	40 °C	18.4 μM ± 3%	37–48	144 μM ± 6%	10.2 μM ± 12%	21 °C	16 μM ± 24%	Figure 6

K_d_ were determined from nDSF data by isothermal analysis at the selected temperature. The change of heat capacity upon protein unfolding (∆C_p_) was assumed to be zero for all nDSF analyses. A melting temperature analysis with a single-site model [model 1, Eq. (1)] and an alternative model [model 2, Eq. (4)] was used to determine apparent binding affinities K_d,app_.

### ADPR-binding proteins

**Figure 3:**
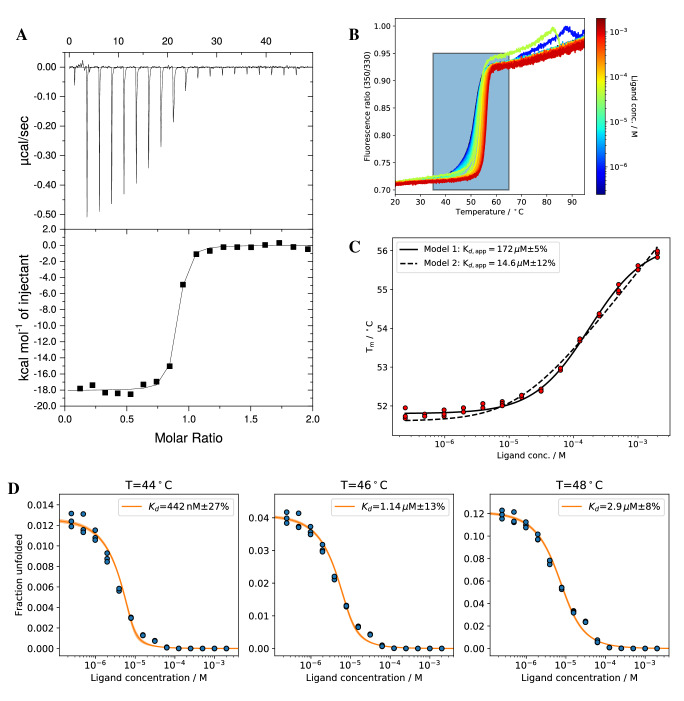
(**A**) The Buffer subtracted ITC of EG1/ADPR yields *K*_*d*_ = 16 ± 4 nM (3 measurements) (**B**) nDSF signal (Ratio) for EG1/ADPR binding study with a protein concentration of 8 µM and ligand concentrations between 2 mM and 24 nM (14 dilutions). The region in the colored box was used for isothermal analysis (shown in **D**). (**C**) Melting temperature analysis with two different models yields apparent *K*_d,app_ values. (**D**) Isothermal analysis of nDSF data for ∆*C*_*p*_ = 0 at three selected temperatures. The same analysis for a fitted ∆*C*_*p*_ (cf. Table 1) is shown in Supplementary Information S25.

**Figure 4:**
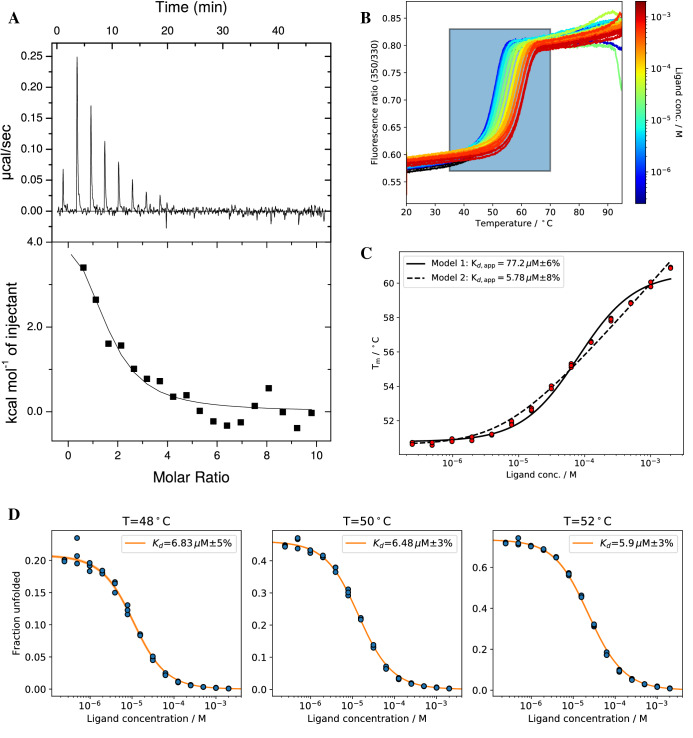
(**A**) The buffer subtracted ITC experiment for SS1/ADPR yields a *K*_*d*_ = 3.5 µM (**B**) nDSF signal (fluorescence ratio F_350*/*330_) for SS1/ADPR binding study. The region in the colored box was used for isothermal analysis (shown in D). The ligand concentrations are color code from blue (low conc.) to red (high conc). The apo protein spectrum in absence of ligand is shown in black. (**C**) Melting temperature analysis with two different models yields apparent *K*_*d,app*_ values. (**D**) Isothermal analysis of nDSF data for ∆*C*_*p*_ = 0 at three selected temperatures. The same analysis for a fitted ∆*C*_*p*_ (cf. Table 1) is shown in Supplementary Information S26.

As first model systems we have investigated two ADPR-binding proteins. EG1 (human) and SS1 (zebrafish) are soluble proteins that bind the physiological ligand adenosine diphosphate ribose (ADPR) enabling a variety of downstream signalling events. According to ITC experiments, EG1/ADPR shows a high nM affinity (16 nM at 20 °C, Fig. 3A) whereas SS1/ADPR shows a low micromolar affinity (3.5 μM at 25 °C, Fig. 4A). Only comparing *T*_*m*_ changes for different systems can lead to wrong conclusions (Figs. 3B and 4B). Even though SS1 shows a much larger melting temperature change than EG1 (5 °C vs. 10 °C, binding is tighter in case of EG1. These wrong conclusions are supported by the apparent binding affinity ${K}_{d,app}$ which is higher for EG1 than for SS1 (Figs. 3C and 4C). For EG1, model 1 fails completely, the respective ${K}_{d,app}$ is 4 orders of magnitude higher than the value determined by ITC. Model 2 performs better but the ${K}_{d,app}$ is still 3 orders of magnitude off. In contrast to that, isothermal analysis of nDSF binding studies not only reflects the trend in binding affinities but also the same order of magnitude compared to ITC. The temperature to extract K_d_ values is, however, limited to the region around the melting temperature. For EG1, the determined K_d_ values in the temperature range between 44 and 48 °C range from 442 nM at 44 °C to 2.9 μM at 48 °C (Fig. 3D). This strong apparent temperature dependence is a possible explanation for the significantly lower *K*_*d*_ value of 16 nM determined by ITC at 20 °C. Contrary to that behavior, isothermal analysis of SS1/ADPR yields stable binding affinities between 6.8 and 5.9 μM in the temperature range between 48 and 52 °C (Fig. 4D) which are in good agreement with the *K*_*d*_ value of 3.5 μM determined by ITC at 25 °C.

### Major histocompatibility complex class I

**Figure 5:**
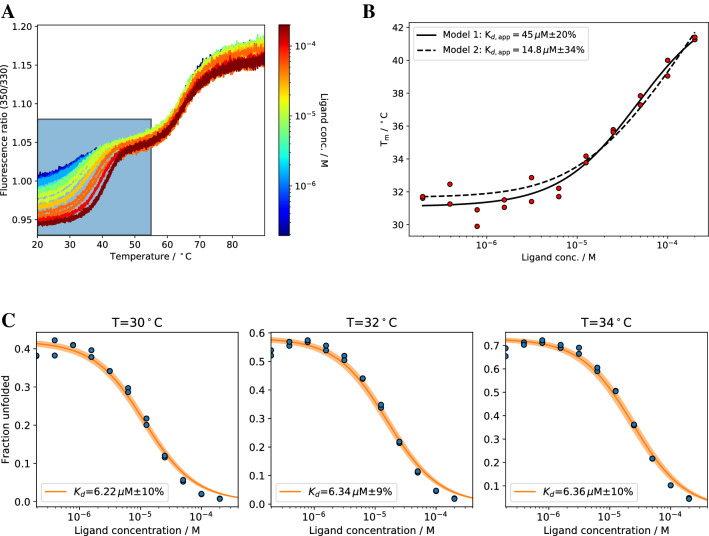
(**A**) nDSF binding study between MHC and the peptide NT8 (fluorescence ratio F_350*/*330_). The region in the colored box was used for isothermal analysis (shown in **C**). The ligand concentrations are color code from blue (low conc.) to red (high conc). (**B**) Melting temperature analysis with two different models yields apparent *K*_d,app_ values. (**C**) Isothermal analysis with a ∆*C*_*p*_ = 0. The same analysis for a fitted ∆*C*_*p*_ is show in supplementary information S27.

Major histocompatibility complex (MHC) class I molecules are an important component in the immune response^23–25^. These molecules bind peptides and present them at the cell surface to mediate T cell immune surveillance. nDSF has already been successfully applied to assessing the thermal and kinetic stability of MHC/peptide complexes^26^. We have performed a nDSF binding study of empty MHC molecules of the HLA-A*02:01 allotype with the suboptimal peptide NT8 (sequence, NLVPMVAT; Fig. 5). Isothermal analysis at 32 °C results in a binding affinity of 6.3 μM, close to the value of 17.0 µM predicted by the NetMHC neural network^27^. Similarly to the case shown before, the apparent *K*_*d,app*_ for model 1 is considerably higher than the value determined by isothermal analysis, while model 2 yields a similar K_d,app_.

### Peroxisomal-coenzyme A synthetase

**Figure 6:**
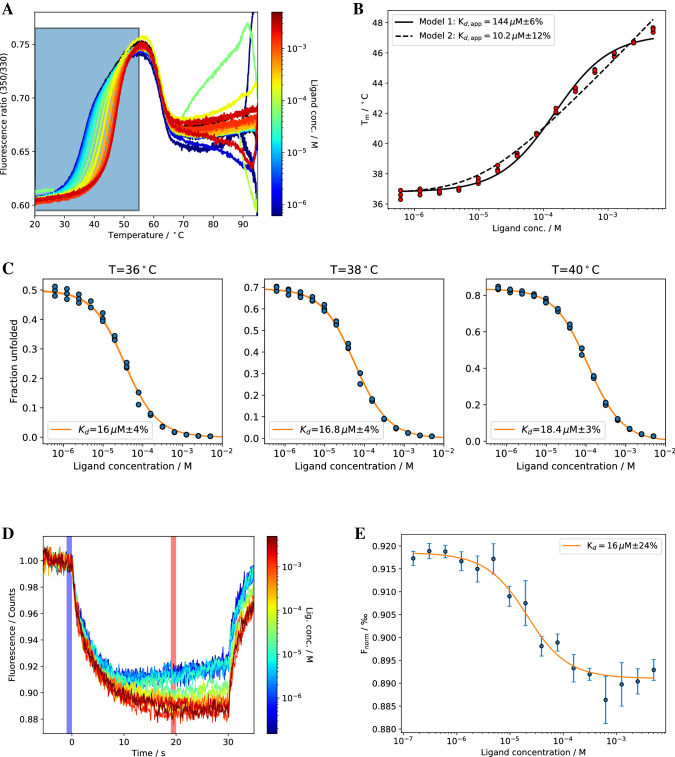
(**A**) nDSF signal (fluorescence ratio) for Pcs60/*γ*S-ATP titration. The region in the colored box was used for isothermal analysis (shown in **C**). (**B**) Melting temperature analysis with two different models yields apparent *K*_d,app_ values. (**C**) Isothermal analysis of nDSF data for ∆*C*_*p*_ = 0 at three selected temperatures. The same analysis for a fitted ∆*C*_*p*_ (cf. Table 1) is shown in supplementary information S28. (**D**) MST experiment with Pcs60/*γ*S-ATP. The “cold” and “hot” regions used for calculating F_norm_ are marked as blue and red shadows, respectively. (**E**) Fitting F_norm_ to a 1:1 model yields a *K*_*d*_ of 16 µM.

We have also applied isothermal analysis to the Peroxisomal-coenzyme A synthetase (Pcs60), which is involved in the peroxisomal fatty-acid metabolism^28^. It catalyzes the formation of oxalyl-CoA from oxalate, ATP and CoA^29^. Similarly, to DSC measurements (cf. S24), nDSF melting curves for Pcs60 in presence of different concentrations of the nucleotide γS-ATP show two transitions around 40 and at 62 °C (Fig. 6A). The second transition does not shift upon addition of the ligand, whereas the first transition is affected by the presence of the ligand. Hence, the first transition was chosen for isothermal and melting temperature analysis, which yields an apparent *K*_*d,app*_ of 152 μM for model 1 and 10.2 μM for model 2, respectively (Fig. 6B). Isothermal analysis of the nDSF data at 40 °C yields a *K*_*d*_ of 18 μM (Fig. 6C). To evaluate isothermal analysis we have done microscale thermophoresis with the same system at 21 °C (MST, Fig. 6D + E). This method yields a *K*_*d*_ of 16 μM, which agrees very well with isothermal analysis and model 2.

To test whether our method is sensitive to defects in ligand binding, we generated a Pcs60 K523A mutant. Lys523 is conserved among different Pcs60 sequences (cf. Fig. S30) and was shown to be crucial for nucleotide binding and catalytic activity in a related Pcs60 enzyme^30^. An isothermal analysis of the nDSF data at 40 °C yields a *K*_*d*_ of 213 μM for nucleotide binding of the K523A mutant (Supplementary Information S31) while the melting temperature analysis yields a higher *K*_*d*_ of 741 μM when using model 1 and a lower *K*_*d*_ of 117 μM when using model 2.

## Discussion

For all cases shown here, binding affinities from isothermal analysis are in good agreement with the ones from isothermal titration calorimetry (ITC), microscale thermophoresis (MST) or a neural network prediction (MHC-I). Binding affinities for EG1/ADPR obtained by isothermal analysis show a strong temperature dependence. Sub μM affinities were retrieved, but only at low temperatures (${T}_{iso}=44$°C) far away from the melting temperature (${T}_{m}=51$°C). At these temperatures, the dynamic range of the fraction of unfolded protein is relatively low, which results in a larger relative error. The lowest fitting error can usually be obtained for a ${T}_{iso}$ around the ${T}_{m}$ of the protein in the absence of ligand. Apparent binding affinities from melting temperatures are considerably higher when using model 1. Together with the lack of physico-chemical foundations, this clearly shows that this model should not be used to estimate binding affinities. Affinities determined by model 2 are in good agreement with isothermal analysis as long as the affinities are above 1 μM. For sub-μM affinities, isothermal analysis performs better for our example system. This is supported by an additional binding study with a nM binder pknG and its inhibitor AX20017^31^ shown in the supplementary information (Supplementary Information, Figs. S34–S35). For SS1 and EG1, the apparent K_d_s do not show the same trend compared to the affinities determined by ITC. The shape of the fitted curves can deviate from the experimental melting temperatures for both model 1 and 2 (cf. Figs. 4B and 6B), due to the thermodynamical incorrectness of model 1 or the approximation [L] = [L]_0_ for model 2. Apparent ${K}_{d}$ values with thermodynamically incorrect model 1 in Eq. (1) needs to be treated with caution. Model 2 yields in general good results but one could expect deviations when determining nM binding affinities. For Pcs60, isothermal analysis yielded binding affinities in agreement with microscale thermophoresis data confirming the crucial role of a particular active site lysine residue for nucleotide binding.

Our results show that isothermal analysis can be used to determine binding affinities in the nM to high μM range. Compared to a simple melting temperature analysis, the binding affinities are in better agreement with isothermal titration calorimetry, especially for nM binders. This label-free method requires a relatively low amount of protein sample when compared to other biophysical methods like ITC. Assuming that most proteins would contain at least one tryptophan residue, the concentration has to be adjusted to the detection range of the instrument, with an average working concentration around 0.1–0.2 mg/ml. When compared to the isothermal analysis performed on Thermofluor data, it is important to remember that most standard dyes are not compatible with membrane proteins solubilised in detergents. In addition, the Thermofluor assay reports unfolding/aggregation when hydrophobic patches are accessible while the nDSF assay reports the exposure of buried tryptophan residues upon protein unfolding. For Pcs60, nDSF experiment provided separated transitions that could be easily analyzed in contrast to the respective Thermofluor experiment (Supplementary Information S37). In our experience, the nDSF isothermal analysis is a suitable probe for intermediate to low binders (high nM to mM) where other biophysical techniques have limitations. The fact that autofluorescence of ligands does not seem to be of major concern when nucleotides have been analysed shows the robustness of the technique. Also, this method can be used to determine binding constants at higher temperatures (e.g., 37 °C), where a fraction of protein is already partly unfolded. Finally, we have developed a web server that is fast and easy to use. Our code reduces the calculation time for the computational bottleneck, which is global fitting of the thermal curves down to 5–10 s (45 curves with 649 points each). This server is now publicly available at https://spc.embl-hamburg.de and removes one obstacle in the application of isothermal analysis, namely easy access without programming skills. In the following, we will discuss common questions and criticism when it comes to the application of this method.

### Choice of fluorescence signal and temperature range

Isothermal analysis can be applied to both intrinsic and extrinsic (external dye) fluorescence data. In the case of nDSF there are two quantities obtained during the measurements. Thermal unfolding can be simultaneously followed by the fluorescence at 330 nm or 350 nm. In many cases the unfolding can be visually most easily followed by the ratio between the fluorescence intensities at 350 nm and 330 nm, since they mostly show a different trend upon unfolding. However, there can be also exceptions where this ratio is not a good measure of unfolding^32^. In all the cases shown in this work, the fluorescence ratio was a good measure of protein unfolding and obtaining binding affinities. One exception is shown in the supplementary information (Fig. S34–S36) where different ligand quenching effects on the intrinsic protein fluorescence are observed for 330 and 350 nm. This distorts the fluorescence ratio and prevents a proper analysis. It is therefore important to keep in mind to try the individual signals, in case the fluorescence ratio does not give a satisfying result. As far as the choice of temperature for isothermal analysis is concerned, we recommend to scan a wide region around the melting temperature of interest and to use the fitting error as a measure of quality. This will be exemplified later (cf. Fig. 8) by the heating rate experiments. Fitting a binding model to the fraction unfolded is fast and therefore not a computational bottleneck during isothermal analysis.

### Reversibility of unfolding and effect on isothermal analysis

One prominent point of criticism when it comes to analysing nDSF data is the degree of irreversibility of the unfolding process during heating. In the ideal case, the protein unfolding is a two-state process that is completely reversible [cf. Eq. (5)]. The data analysis scheme presented here uses this model. However, irreversible unfolding can occur, which can affect the results of data analysis^33^. One example is the dependence of the apparent ${T}_{m}$ on the heating rate used during the experiment^9^. In the following two subsections, we present experiments to assess (I) the effect of the heating rate on melting temperatures and binding affinities and (II) the reversibility of unfolding for two systems presented earlier: SS1/ADPR and Pcs60/$\gamma$S-ATP.

**Figure 7:**
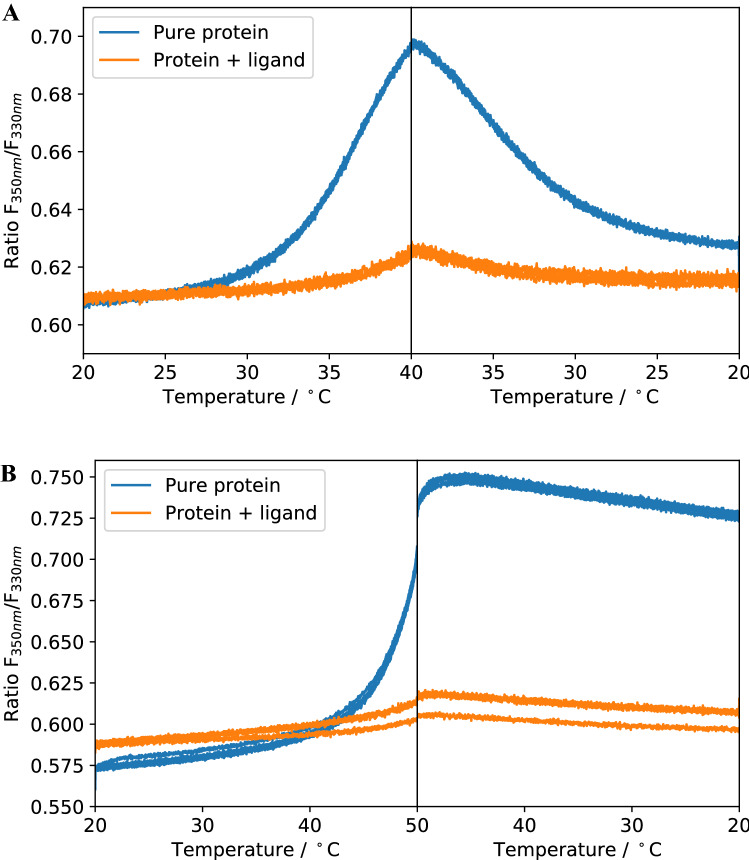
Refolding experiment with Pcs60 (**A**) and SS1 (**B**) with a maximum temperature set to the temperature at which *K*_*d*_ values were extracted by isothermal analysis in Figs. 4 and 6 (40 °C for Pcs60 and 50 °C for SS1). Refolding experiment for Pcs60 up to 50 °C and SS1 up to 45 °C are shown in S32.

To investigate the irreversibility of thermal unfolding we performed refolding experiments. For these experiments, the protein is heated up to a defined temperature and then cooled down at the same rate until the starting temperature is reached. Both during heating and cooling, the fluorescence signal is acquired. The maximum temperature chosen here is related to the temperature at which the error of the isothermal analysis is minimal. These temperatures are 40 °C for Pcs60/$\gamma$S-ATP and 50 °C for SS1/ADPR for a heating rate of 1 °C/min (Table 3). While Pcs60 unfolding is mostly reversible, SS1 unfolds irreversibly to a large extent (Fig. 7). This is even the case when decreasing maximum temperature for SS1 to 45 °C and increasing it for Pcs60 to 50 °C (Fig. S32).

**Table 3:** Summary of isothermal analysis for different heating rates for Pcs60/gS-ATP and SS1/ADPR for different heating rates shown in Fig. 8.

Pcs60/gS-ATP									
Heating rate	5 °C/min			3 °C/min			1 °C/min		
	*T*_*m*_	*T*_sel_	$K_{{{\text{d}},{\text{T}}_{{{\text{sel}}}} }}$	*T*_*m*_	*T*_sel_	$K_{{{\text{d}},{\text{T}}_{{{\text{sel}}}} }}$	*T*_*m*_	*T*_sel_	$K_{{{\text{d}},{\text{T}}_{{{\text{sel}}}} }}$
**∆C**_***p***_									
Default: 0 kcal/mol	38 °C	39 °C	23 µM	37 °C	39 °C	16 µM	36 °C	40 °C	18 µM
Fitted: 6.7 kcal/mol	37 °C	40 °C	24 µM	37 °C	39 °C	19 µM	35 °C	38 °C	17 µM

SS1/ADPR									
Heating rate	3 °C/min			1 °C/min			0.1 °C/min		
	*T*_*m*_	*T*_sel_	$K_{{{\text{d}},{\text{T}}_{{{\text{sel}}}} }}$	*T*_*m*_	*T*_sel_	$K_{{{\text{d}},{\text{T}}_{{{\text{sel}}}} }}$	*T*_*m*_	*T*_sel_	$K_{{{\text{d}},{\text{T}}_{{{\text{sel}}}} }}$
**∆C**_***p***_									
Default: 0 kcal/mol	53 °C	53 °C	6.7 µM	50 °C	51 °C	6.3 µM	46 °C	45 °C	6.2 µM
Fitted: 7.6 kcal/mol	53 °C	54 °C	6.4 µM	50 °C	52 °C	6.2 µM	46 °C	46 °C	7.6 µM

The analyse were done for two different ∆C_p_ values: a default value of 0 and the fitted value for the 1 °C/min measurement (cf. Table 1). T_sel_ is the temperature with the lowest fitting error for K_d_. For Pcs60 we have used the heating rates 7, 5, 3 and 1 °C/min. For SS1 heating rates of 3, 1 and 0.1 °C/min are shown.

### Influence of heating rate and choice of temperature for isothermal analysis

The heating rate during the nDSF experiment can have a strong influence on the apparent melting temperature ${T}_{m}$. This is caused by an irreversible unfolding step that is kinetically controlled. There are models that take into account this third state^9^ using a kinetic parameter. The relevant question here is how much the determination of binding affinities are affected by this shift in apparent melting temperatures.

**Figure 8:**
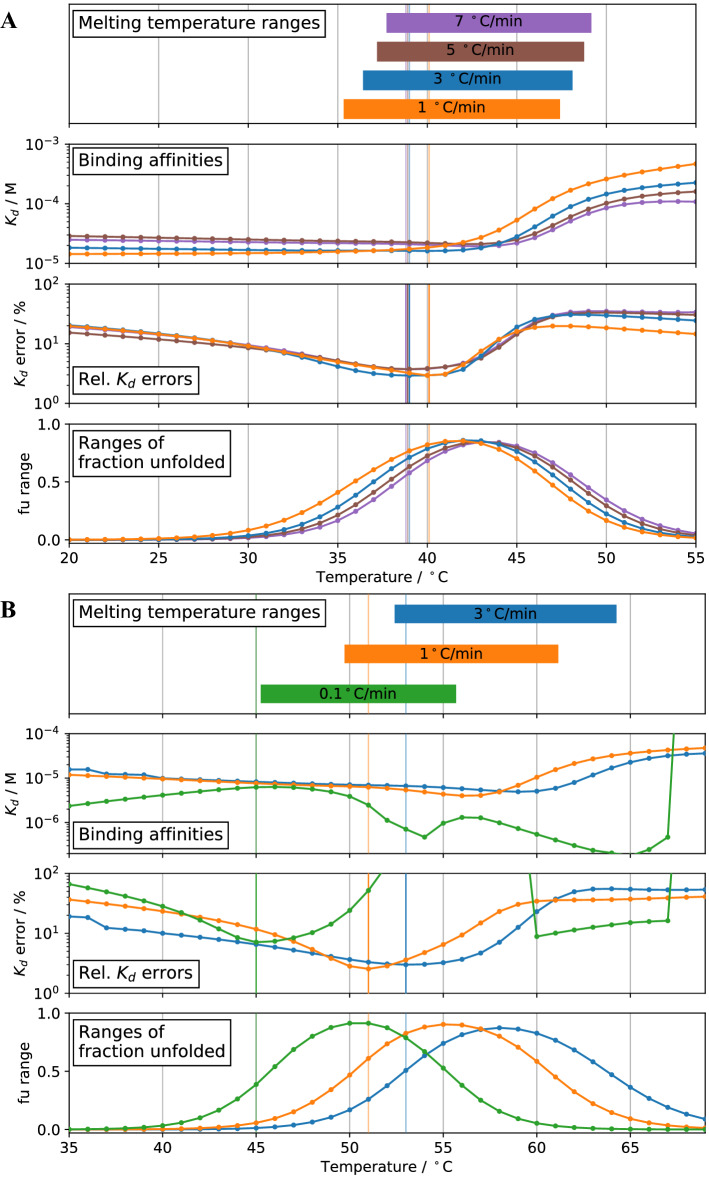
Isothermal analysis at different heating rates for Pcs60/*γ*S-ATP (**A**) and SS1/ADPR (**B**). For all datasets, the ∆*C*_*p*_ = 0 was assumed. The binding affinities were extracted at the temperature with minimum *K*_*d*_ fitting error marked by vertical lines (lines are horizontally shifted for visibility). All temperature and values are summarized in Table 3. The analyses for a fitted ∆*C*_*p*_ for a heating rate of 1 °C/min (cf. Table 1) are shown in Supplementary Information S33.

A prior question is which temperature(s) to choose for the isothermal analysis. As mentioned above, we have calculated ${K}_{d}$ values for a broad range of temperatures around the melting temperature, e.g. the whole temperature window of the experimental data. All of them yield a ${K}_{d}$ and the errors of the respective fit, similarly to the examples shown in Fig. 8. As shown in this figure, the ${K}_{d}$ fitting error usually undergoes a minimum for temperatures around the melting temperature of the apo protein in absence of the ligand. For the analysis shown here we have used the numerical error of the fit as a criterion for choosing the temperature at which to extract a ${K}_{D}$ (shown as vertical lines in Fig. 8).

To test the influence of the heating rate on the determination of binding affinity, we have done nDSF binding studies with different heating rates: 7, 5, 3 and 1 °C/min for Pcs60/γS-ATP and 3, 1 and 0.1 °C/min for SS1/ADPR and analyzed the data by isothermal analysis. The analysis for $\Delta {C}_{p}=0$ kcal/mol are shown in Fig. 8. The respective analysis for fitted $\Delta {C}_{p}$ values (cf. Table 1) are shown in supplementary information S33. All results are summarized in Table 3. As far as apparent melting temperatures are concerned, Pcs60 and SS1 exhibit a very different behaviour upon change of the heating rate (Fig. 8A and B, top panels). Pcs60 only shows a slight increase of apparent melting temperatures with increased heating rates, even for very fast heating. In contrast to that, the apparent melting temperatures for SS1 increase drastically by 7 °C when changing heating rates from 1 to 3 °C/min. This might be an additional indication that Pcs60 is less prone to irreversible unfolding processes than SS1 and corroborates the results from the refolding experiments. As seen in the second panel of Fig. 8B, the determined affinities differ depending on which temperature is chosen for isothermal analysis and depending on the heating rate. However, choosing the temperature of minimal fitting error, provides similar binding affinities ${K}_{d}$ (Table 3). We still recommend using the same data acquisition parameters when comparing binding affinities in a systematic study. Using a $\Delta {C}_{p}=0$ or the fitted $\Delta {C}_{p}$ (cf. Table 1) does not result in differences in K_d_ determination for these systems.

Even though unfolding for the systems described here might not be simple two-state unfolding processes (e.g. Pcs60: DSC in fig. S24; full nDSF in Fig. 4) and not reversible (e.g. SS1), isothermal analysis with a simple two-state unfolding model still could reproduce the binding affinities determined by isothermal titration calorimetry or microscale thermophoresis for the systems presented here.

### Outlook: application to more complex binding stoichiometries

One feature of the data analysis approach shown here is the simplicity of the thermodynamic model used. Fitting the thermal unfolding curves (both local and global) does not require any information of the binding stoichiometry (cf. Fig. 1). The parameters retrieved from these fittings are only two parameters $\Delta H$ and ${T}_{m}$ of protein unfolding. These parameters are used to obtain the fraction unfolded ${f}_{u}$ at a chosen temperature vs. ligand concentration, which are subsequently fitted to a binding model. All the examples shown in this work are 1:1 binders and thus a 1:1 binding model was chosen for fitting the ${f}_{u}$. However, the analysis scheme can also be applied to more complex binding models. The only requirement is a mathematical model to describe the fraction unfolded ${f}_{u}$ as a function of the ligand concentration with the binding affinities as parameters. This includes analytical or numerical expressions for the equilibrium concentrations of the different species. The model has to take into account which species can be distinguished by the fluorescence signal. Normally, DSF will distinguish only between the folded and unfolded species. However, quenching effects (e.g. at different binding sites) can require a more complicated model.

## Methods

### Protein expression and purification

#### Human EG1 and zebrafish SS1 proteins

Human (hs) EG1 was produced with a TEV-cleavable C-terminal His10-tag using the pET15b(+) vector. The sequence coding for the zebrafish (dr) SS1 was cloned into the pnEK-vH vector to generate a construct with a TEV-cleavable N-terminal His6-tag. Protein production was carried out in BL21 Gold (DE3) *E. coli* bacteria in Terrific Broth (TB) media. Cells were grown at 37 °C to a cell density of 0.6–0.9 (measured at 600 nm) and after induction with 0.1 mM isopropyl-$\beta$-d-1-thio-galactopyranoside (IPTG), the target sequences were expressed overnight at 20 °C. The cells were harvested by centrifugation (5000×*g* for 25 min) and lysed by sonication or homogenization. Lysates were centrifuged (39,000×*g* for 45 min) and the tagged proteins were purified from the supernatant using immobilized metal affinity chromatography (IMAC). After His-tag removal by TEV protease and a second round of IMAC, the target proteins were further purified by gel filtration (Superdex 200 Increase 10/300 GL) using buffer N (for EG1; 20 mM HEPES pH 7.5, 150 mM NaCl, 5 mM CaCl_2_) or buffer M (for SS1; 25 mM HEPES pH 7.5, 150 mM NaCl, 5 mM CaCl_2_). Peak fractions were pooled and protein identity confirmed by SDS-PAGE and mass spectrometry.

#### MHC-I

HLA-A heavy chain disulfide mutant (dsA2) and human β2m light chain (hβ2m) were expressed and purified as described by Anjanappa et al*.*^23^. Briefly, expression was performed in *E. coli* Rosetta BL21 (DE3) pLysS using pET plasmid under the control of a T7 promoter. Cells were pelleted and lysed in cell lysis buffer (25% sucrose, 1 mM EDTA, 1 mM PMSF, 10 mM DTT in 50 mM Tris–Cl pH 8.0). Inclusion bodies were harvested by centrifugation, washed three times with detergent buffer (50 mM Tris pH 8.0, 150 mM NaCl, 0.5% Triton X 100, 1 mM DTT), followed by final wash with TBS (50 mM Tris pH 8.0, 150 mM NaCl, 1 mM DTT). The pellet was dissolved in solubilization buffer (6 M Guanidine HCl, 50 mM HEPES pH 6.5, 0.1 mM DTT, 0.1 mM EDTA) for 24 h at 4 °C. The solubilized protein was centrifuged at 30,000 × g for 30 min and the supernatant fraction was stored at − 80 °C until use. The refolding reaction was performed by diluting 1 μM of dsA2 heavy chain and 2 μM of hβ2m in a refolding buffer (100 mM Tris·Cl pH 8, 0.5 M arginine, 2 mM EDTA, 0.5 mM oxidized glutathione, 5 mM reduced glutathione, 10 mM dipeptide GM (Bachem) and incubated for 4–5 days at 4 °C with constant stirring, followed by protein concentration using 30 kDa cutoff membrane filters (Vivaflow-200; Sartorius). The concentrated protein was purified by SEC in 20 mM Tris–HCl pH 8.0, 150 mM NaCl on an ÄKTA system (GE healthcare) using a HiLoad Superdex 200 16/600 column (GE healthcare).

#### Pcs60

Full length *S. cerevisiae* Pcs60 was cloned in a pETM14 vector. Pcs60 was expressed in BL21 *E. coli* strain grown in auto-induction medium^34^, with 5 h at 37 °C and 16 h at 18 °C. Cells were harvested, resuspended in lysis buffer (50 mM HEPES pH 7.5, 150 mM NaCl, 20 mM imidazole, protease inhibitor (Roche), DNAse (Sigma) and lysozyme (Sigma)), homogenized 1 h at 4 °C and lysed by sonication. Lysates were then cleared by centrifugation and the supernatant loaded onto Ni–NTA resin (Qiagen). Bound proteins were washed with 50 mM HEPES pH 7.5, 750 mM NaCl, 20 mM Imidazole and the protein eluted with 50 mM HEPES pH 7.5, 150 mM NaCl, 250 mM Imidazole. The eluate was then dialysed against HEPES pH 7.5, 150 mM NaCl, 0.5 mM TCEP and simultaneously digested with 1 mg of TEV-protease per 100 mg of protein. Undigested protein and TEV protease were removed by a second Ni–NTA step and flow through containing Pcs60 was concentrated to 5 ml for gel filtration (HiLoad 16/60 Superdex 200 pg, GE healthcare) using an ÄKTA pure purification system. Relevant fractions were pooled together and the protein was concentrated, flash frozen in liquid nitrogen and stored at − 80 °C.

For the binding studies we have selected a Pcs60 variant with optimized oligomerization behavior. This construct shows very similar binding affinities compared to the wild-type PCS60 (cf. Fig. S29) and a larger melting temperature range with lower melting temperatures (cf. Fig. 6A and Fig. S29A).

### Isothermal titration calorimetry (ITC)

ITC measurements for Human EG1 and zebrafish SS1 proteins were carried out on a MicroCal ITC-200 isothermal titration calorimeter (Malvern Panalytcal, Malvern, UK) and thermodynamic parameters were analysed using the MicroCal Origin program. Measurements were performed at 20 °C in buffer N (for EG1) or at 25 °C in buffer M (for SS1). The ligand ADPR was dissolved in the respective buffer. After an initial injection of 0.5 μl, 18 regular injections of 2 μl of ADPR were added to 10–20  μM EG1 or SS1 in the sample cell. The individual injections were interspaced by 150 s and stirring speed was set to 750 rpm. Baseline corrections were obtained by titrating ADPR into the corresponding buffer.

### Microscale thermophoresis (MST)

MST curves for Pcs60/$\gamma$S-ATP were acquired with a Nanotemper Monolith NT.LabelFree (Nanotemper) using the MO.Control v1.6 acquisition software and Monolith NT.LabelFree capillaries. The same protein concentration and buffer conditions as for the nDSF experiments were used (9.9 μM). 16 different ligand concentrations (serial dilution with a factor of 2) in the range between 5 mM and 0.15 μM were used for each measurement. For the MST experiment the IR laser power was set to 20% and the UV laser power to 1%. In total, two independent measurements were done plus an additional repeat. The data was exported as xlsx and analyzed and plotted with a self-written Python script. The regions for F_norm_ determination were − 1–0 s for “cold” and 19–20 s for “hot”. For each curve F_norm_ was calculated as the ratio between the mean values in the “hot” and “cold” region. In the final step, F_norm_ was fitted to a 1:1 binding model. The self-written Python scripts for spectral analysis including MST analysis are available online: https://github.com/steniegit/libspec

### Sequence alignment

Sequence alignment of *Saccharomyces cerevisiae* Pcs60 (UniProt P38137) and *Arabidopsis thaliana* AAE3 (UniProt Q9SMT7) were performed using MUSCLE^35^ and visualized with Jalview^36^.

### nDSF experiments: general information

All nDSF temperature curves were acquired with a Nanotemper Prometheus NT.48 fluorimeter (Nanotemper) controlled by PR.ThermControl (version 2.1.2). Excitation power was pre-adjusted to obtain fluorescence readings above 2000 RFU for fluorescence emission at 330 nm (F330) and 350 nm (F350), and samples were heated from 20 to 95 °C with a rate of 1 °C/min unless otherwise stated. For all measurements, Prometheus NT.48 series nDSF grade standard capillaries (Nanotemper, Munich, Germany) were used. For each binding study a serial dilution series of the ligand (dilution ratio 2) at constant protein concentration was prepared. In addition to the ligand dilutions, one pure protein sample was used. If not stated otherwise, all binding studies were performed in duplicates.

### nDSF binding studies

#### Human EG1 and zebrafish SS1 proteins

Binding studies of EG1 and SS1 were performed in the same buffers as mentioned in the protein expression and purification section at protein concentrations of 8 μM and 5 μM, respectively. The ligand ADPR was dissolved in the same buffer and a dilution series of a solution containing the protein at a constant concentration (as shown above) and ADPR concentrations between 2 mM and 25 nM (14 dilutions, triplicates) were used for the nDSF study (LED power 10%).

#### MHC-I

11 dilutions of NT8 peptide ligand (NLVPMVAT) starting at 200 μM in the presence of 2.2 μM of MHC-I in SEC buffer (20 mM Tris pH 8, 150 mM NaCl) were prepared. Peptide was purchased from Genecust (Boynes, France) with 90% purity. The dilutions span a ligand concentration range between 200 μM and 195 nM with a dilution factor of 2 between each point. After 30 min incubation at room temperature, the samples were transferred to nDSF capillaries (duplicates) and nDSF curves were acquired with a LED power of 70%.

#### Pcs60

The following protocol was used for all nDSF experiments with Pcs60 and the nucleotide ligand γS-ATP. 14 dilutions of the nucleotide ligand (γS-ATP) starting at 5 mM at a constant Pcs60 concentration of 9.9 μM were prepared in buffer (50 mM HEPES pH 7.5, 150 mM NaCl, 0.5 mM TCEP). The dilutions span a ligand concentration range between 5 mM and 610 nM with a dilution factor of 2 between each point. In addition to the ligand dilutions, a pure Pcs60 sample in the same buffer was used. If not stated otherwise, the measurements were run as triplicates (in total 45 temperature curves). The LED laser power for the experiments was between 70 and 80%.

### Isothermal analysis of nDSF data in a nutshell

The isothermal analysis follows the approach by Bai et al*.*^16^. Briefly, obtaining binding affinities from the experimental data is a four-step process. In the first step, each fluorescence curve is fitted individually using six parameters: the melting temperature ${T}_{m}$, the enthalpy of unfolding $\Delta$H, the initial and final intercepts, and the initial and final slopes (combining Eqs. 14, 12 and 9). These values can in principle already be used to continue with the isothermal analysis to obtain a binding affinity. However, an additional global fit of the fluorescence curves using the parameters from the previous fit as input gives more consistent results. In the global fit, the initial and final slope are fitted globally while the other parameters are still fitted individually for each curve. In the third step, the fraction unfolded (${f}_{u}$) is calculated for each ligand concentration at one or several temperatures. These temperatures can be selected in the temperature range, where unfolding of the relevant domain takes place. In the last step, the fraction unfolding vs. the ligand concentrations are fitted to a binding model. Here, we used a 1:1 binding model but the method can be extended to more complex models. The whole process is depicted as flowchart in Fig. 1. More details about data analysis are given in the experimental section and in the supplementary information S10 and figure S38.

### Isothermal fitting routines

Both the local and global fitting routines for the isothermal analysis contain boundaries for the fitted melting temperatures ${T}_{m}$ and the unfolding enthalpy $\Delta H$. The melting temperature is constrained to the experimental temperature region that is selected for the analysis. $\Delta H$ is constrained to be positive. Our program contains an experimental feature that allows to fit the change of heat capacity $\Delta {C}_{p}$ as a global variable in addition to the others. This feature remains to be experimentally validated. All errors specified in the plots are numerical fitting errors derived from the square root of the diagonal elements of the covariance matrix (output of Scipy curve_fit function).

## Supplementary Information

Supplementary Information.

## Data Availability

The data analysis was programmed in Python 3 using the modules SciPy^37^, NumPy^38^, Matplotlib^39^ and Pandas^40^.An interactive web server based on this code is available here: http://spc.embl-hamburg.de.

## References

[1] Linkuvienė V (2018). Thermodynamic, kinetic, and structural parameterization of human carbonic anhydrase interactions toward enhanced inhibitor design. Q. Rev. Biophys..

[2] Wienken CJ, Baaske P, Rothbauer U, Braun D, Duhr S (2010). Protein-binding assays in biological liquids using microscale thermophoresis. Nat. Commun..

[3] Seidel SAI (2012). Label-free microscale thermophoresis discriminates sites and affinity of protein-ligand binding. Angew. Chem. Int. Ed..

[4] Kairys V, Baranauskiene L, Kazlauskiene M, Matulis D, Kazlauskas E (2019). Binding affinity in drug design: Experimental and computational techniques. Expert. Opin. Drug Discov..

[5] Sandoval PJ, Santiago J (2020). In vitro analytical approaches to study plant ligand-receptor interactions. Plant Physiol..

[6] Bruce D, Cardew E, Freitag-Pohl S, Pohl E (2019). How to stabilize protein: Stability screens for thermal shift assays and nano differential scanning fluorimetry in the virus-x project. J. Vis. Exp.

[7] Kotov V (2019). High-throughput stability screening for detergent-solubilized membrane proteins. Sci. Rep..

[8] Hamborg L (2020). Global analysis of protein stability by temperature and chemical denaturation. bioRxiv.

[9] Wu T (2020). Three essential resources to improve differential scanning fluorimetry (DSF) experiments. bioRxiv.

[10] Gao K, Oerlemans R, Groves MR (2020). Theory and applications of differential scanning fluorimetry in early-stage drug discovery. Biophys. Rev..

[11] Matulis D, Kranz JK, Salemme FR, Todd MJ (2005). Thermodynamic stability of carbonic anhydrase: Measurements of binding affinity and stoichiometry using ThermoFluor. Biochemistry.

[12] Cimmperman P (2008). A quantitative model of thermal stabilization and destabilization of proteins by ligands. Biophys. J..

[13] Cimmperman, P. & Matulis, D. Chapter 8. Protein thermal denaturation measurements via a fluorescent dye. In *Biophysical Approaches Determining Ligand Binding to Biomolecular Targets*, 247–274, 10.1039/9781849732666-00247 (Royal Society of Chemistry, 2011).

[14] Matulis D (2019). Carbonic Anhydrase as Drug Target.

[15] Boland C (2018). Membrane (and soluble) protein stability and binding measurements in the lipid cubic phase using label-free differential scanning fluorimetry. Anal. Chem..

[16] Bai N, Roder H, Dickson A, Karanicolas J (2019). Isothermal analysis of ThermoFluor data can readily provide quantitative binding affinities. Sci. Rep..

[17] Vivoli M, Novak HR, Littlechild JA, Harmer NJ (2014). Determination of protein-ligand interactions using differential scanning fluorimetry. J. Vis. Exp..

[18] Yoshida T (2019). Differential scanning fluorimetric analysis of the amino-acid binding to taste receptor using a model receptor protein, the ligand-binding domain of fish t1r2a/t1r3. PLoS ONE.

[19] Schellman JA (1975). Macromolecular binding. Biopolymers.

[20] Jarmoskaite I, AlSadhan I, Vaidyanathan PP, Herschlag D (2020). How to measure and evaluate binding affinities. Elife.

[21] Robertson AD, Murphy KP (1997). Protein structure and the energetics of protein stability. Chem. Rev..

[22] Gómez J, Hilser VJ, Xie D, Freire E (1995). The heat capacity of proteins. Proteins Struct. Funct. Genet..

[23] Anjanappa R (2020). Structures of peptide-free and partially loaded MHC class i molecules reveal mechanisms of peptide selection. Nat. Commun..

[24] Moritz A (2019). High-throughput peptide-MHC complex generation and kinetic screenings of TCRs with peptide-receptive HLA-a*02:01 molecules. Sci. Immunol..

[25] Saini SK (2019). Empty peptide-receptive MHC class i molecules for efficient detection of antigen-specific t cells. Sci. Immunol..

[26] Hellman LM (2016). Differential scanning fluorimetry based assessments of the thermal and kinetic stability of peptide–MHC complexes. J. Immunol. Methods.

[27] Andreatta M, Nielsen M (2015). Gapped sequence alignment using artificial neural networks: Application to the MHC class i system. Bioinformatics.

[28] Blobel F, Erdmann R (1996). Identification of a yeast peroxisomal member of the family of AMP-binding proteins. Eur. J. Biochem..

[29] Foster J, Nakata PA (2013). An oxalyl-CoA synthetase is important for oxalate metabolism inSaccharomyces cerevisiae. FEBS Lett..

[30] Fan M, Xiao Y, Li M, Chang W (2016). Crystal structures of arabidopsis thaliana oxalyl-CoA synthetase essential for oxalate degradation. Mol. Plant.

[31] Walburger A (2004). Protein kinase g from pathogenic mycobacteria promotes survival within macrophages. Science.

[32] Žoldák G, Jancura D, Sedlák E (2017). The fluorescence intensities ratio is not a reliable parameter for evaluation of protein unfolding transitions. Protein Sci..

[33] Svilenov HL, Menzen T, Richter K, Winter G (2020). Modulated scanning fluorimetry can quickly assess thermal protein unfolding reversibility in microvolume samples. Mol. Pharm..

[34] Studier FW (2005). Protein production by auto-induction in high-density shaking cultures. Protein Exp. Purif..

[35] Edgar RC (2004). MUSCLE: multiple sequence alignment with high accuracy and high throughput. Nucleic Acids Res..

[36] Waterhouse AM, Procter JB, Martin DMA, Clamp M, Barton GJ (2009). Jalview version 2: a multiple sequence alignment editor and analysis workbench. Bioinformatics.

[37] Virtanen P (2020). SciPy 1.0: Fundamental algorithms for scientific computing in python. Nat. Methods.

[38] Harris CR (2020). Array programming with NumPy. Nature.

[39] Hunter JD (2007). Matplotlib: A 2d graphics environment. Comput. Sci. Eng..

[40] McKinney, W. Data structures for statistical computing in python. In *Proceedings of the 9th Python in Science Conference*. 10.25080/majora-92bf1922-00a (SciPy, 2010).

