# Supplementary material for: FoldAffinity: binding affinities from nDSF experiments
Source: Sci Rep. 2021 May 5;11:9572. doi: 10.1038/s41598-021-88985-z (PMC8099913; doi:10.1038/s41598-021-88985-z)
Supplement: Supplementary file 1 — Supplementary Information. [file 41598_2021_88985_MOESM1_ESM.pdf]

Supplementary information: FoldAffinity – Binding affinities from nDSF experiments

Stephan Niebling[1,4,*], Osvaldo Burastero[1,6,7], Jérôme Bürgi[1], Christian Günther[1], Lucas Defelipe[1], Simon Sander[2], Ellen Gattkowski[2], Raghavendra Anjanappa[3], Matthias Wilmanns[1,5], Sebastian Springer[3], Henning Tidow[2], and María García-Alai[1,4*]

[1]European Molecular Biology Laboratory, Hamburg Outstation, Hamburg, Germany
[2]Hamburg Advanced Research Centre for Bioorganic Chemistry (HARBOR) & Department of Chemistry, Institute for Biochemistry and Molecular Biology, University of Hamburg, Luruper Chaussee 149, 22761 Hamburg, Germany
[3]Department of Life Sciences and Chemistry, Jacobs University Bremen, 28759 Bremen, Germany
[4]Centre for Structural Systems Biology, Notkestrasse 85, D-22607 Hamburg, Germany
[5]University Hamburg Clinical Centre Hamburg-Eppendorf, Martinistraße 52, 20246 Hamburg, Germany
[6]Departamento de Química Biológica, Facultad de Ciencias Exactas y Naturales, Universidad de Buenos Aires, Intendente Güiraldes 2620, Ciudad Autónoma de Buenos Aires, Argentina
[7]IQUIBICEN-UBA/CONICET, Intendente Güiraldes 2620, Ciudad Autónoma de Buenos Aires, Argentina
[*]Correspondence and requests for materials should be addressed to M.G.A. (email: garcia@embl-hamburg.de) and S.N. (stephan.niebling@embl-hamburg.de)

Contents

1 Simulated data and isothermal analysis (S1–S22) 2
1.1 Examples for simulated data . . . 2
1.2 K_d fittings with 0% noise . . . 3
1.3 K_d fittings with 1% noise . . . 7
1.4 K_d fittings with 2% noise . . . 11
1.5 K_d fittings with 5% noise . . . 15
1.6 K_d fittings with 10% noise . . . 19
1.7 Fitted ΔC_p values . . . 23
2 Differential scanning calorimetry of Pcs60 25
3 Isothermal analyses with fitted ΔC_p for proof-of-principle studies (S25–S28) 26
3.1 EG1/ADPR . . . 26
3.2 SS1/ADPR . . . 27
3.3 MHC/NT8 . . . 28
3.4 Pcs60/γS-ATP . . . 29
4 WT Pcs60 in comparison with the Pcs60 mutant used for this manuscript 30
5 Pcs60_K523A mutant 31
5.1 Sequence alignment between Pcs60 and oxalyl-CoA synthetase from arabidopsis thaliana . . . 31
5.2 Binding study with Pcs60_K523A/γS-ATP . . . 32
6 Reversibility tests at different temperatures as fig. 7 (S32) 33
7 Heating rate tests with fitted ΔC_p (S33) 34
8 nDSF binding study: Protein kinase G (pknG) with inhibitor AX20017 35
9 Comparison between nDSF and Thermofluor for Pcs60/γS-ATP 38
10 Fitting procedure of the isothermal analysis 38
References 40

1 Simulated data and isothermal analysis (S1–S22)

1.1 Examples for simulated data

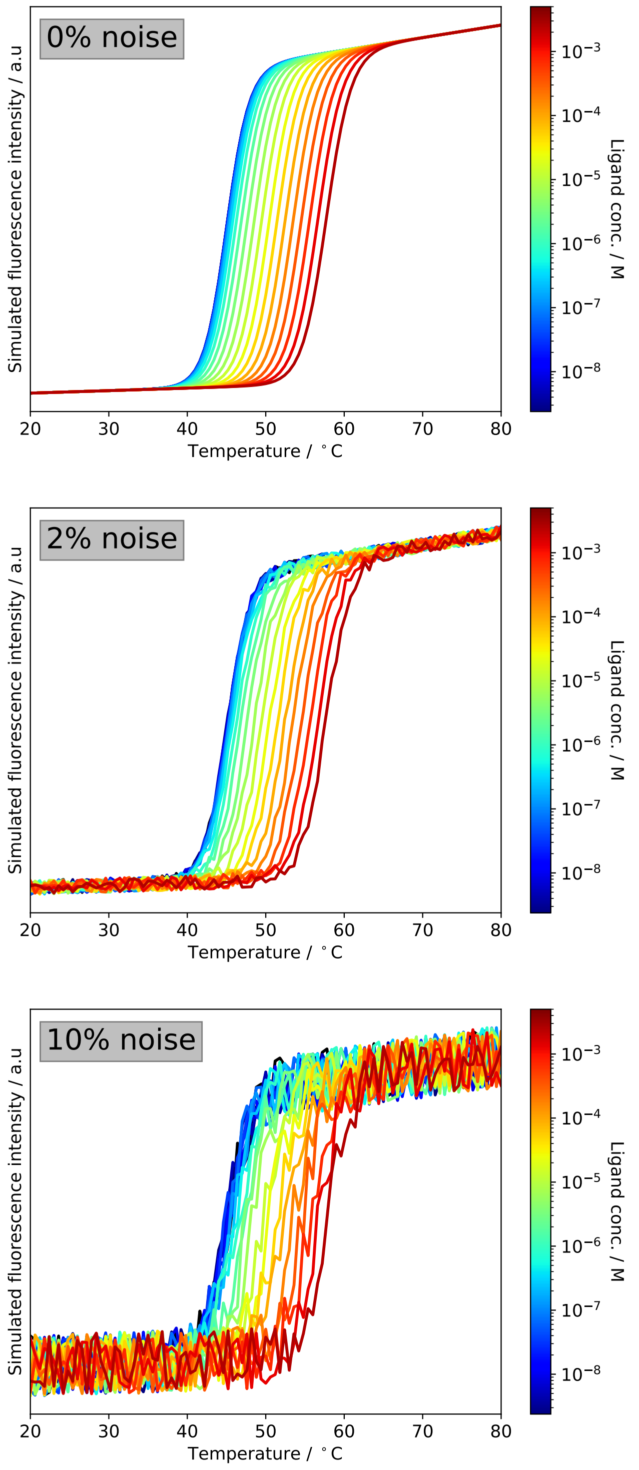

Figure S1. Example of simulated data with $\Delta C_p = 0\,\mathrm{kcal/molK}$ and different noise levels. The dataset shown here was simulated with an initial $K_d = 10^{-6}\,\mathrm{M}$ and a protein concentration $[P]_0 = 10^{-6}\,\mathrm{M}$.

1.2 K_d fittings with 0% noise

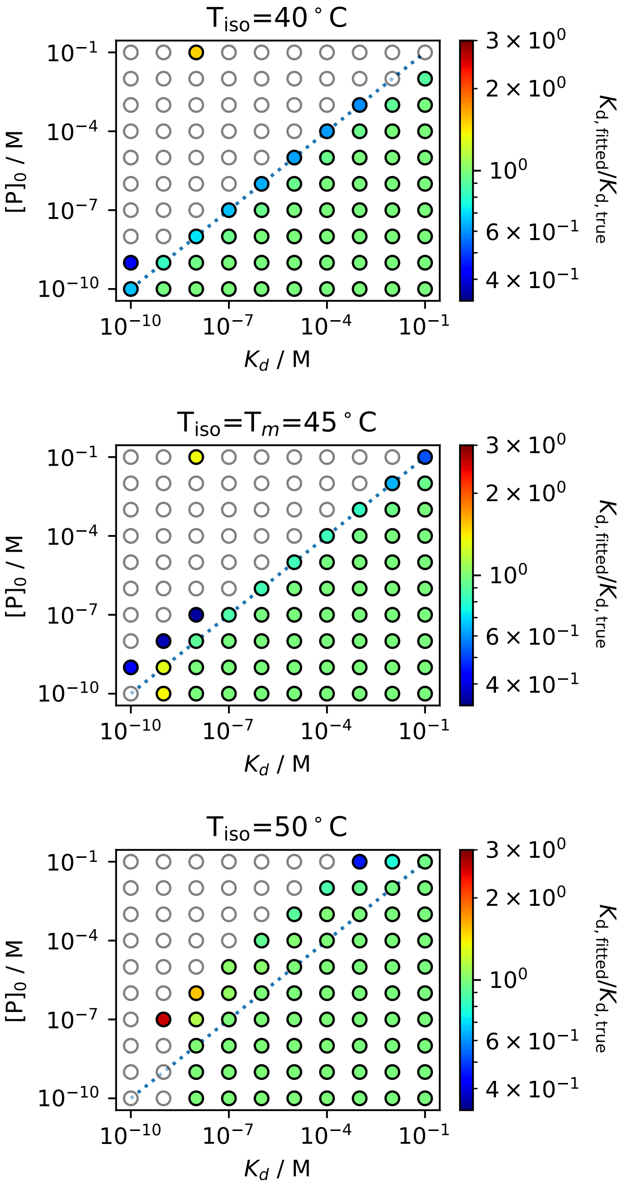

Figure S2. K_d fits for simulated data with $\Delta C_p = 0$ kcal/molK and 0% noise. K_d deviations are color coded with green corresponding to the initial value. Datasets outside the range are shown as white circles. Fitting the ΔC_p during the thermal curve fitting process results in better agreement with the intial K_d values (B) compared to fits with a fixed $\Delta C_p = 0$ kcal/molK (A).

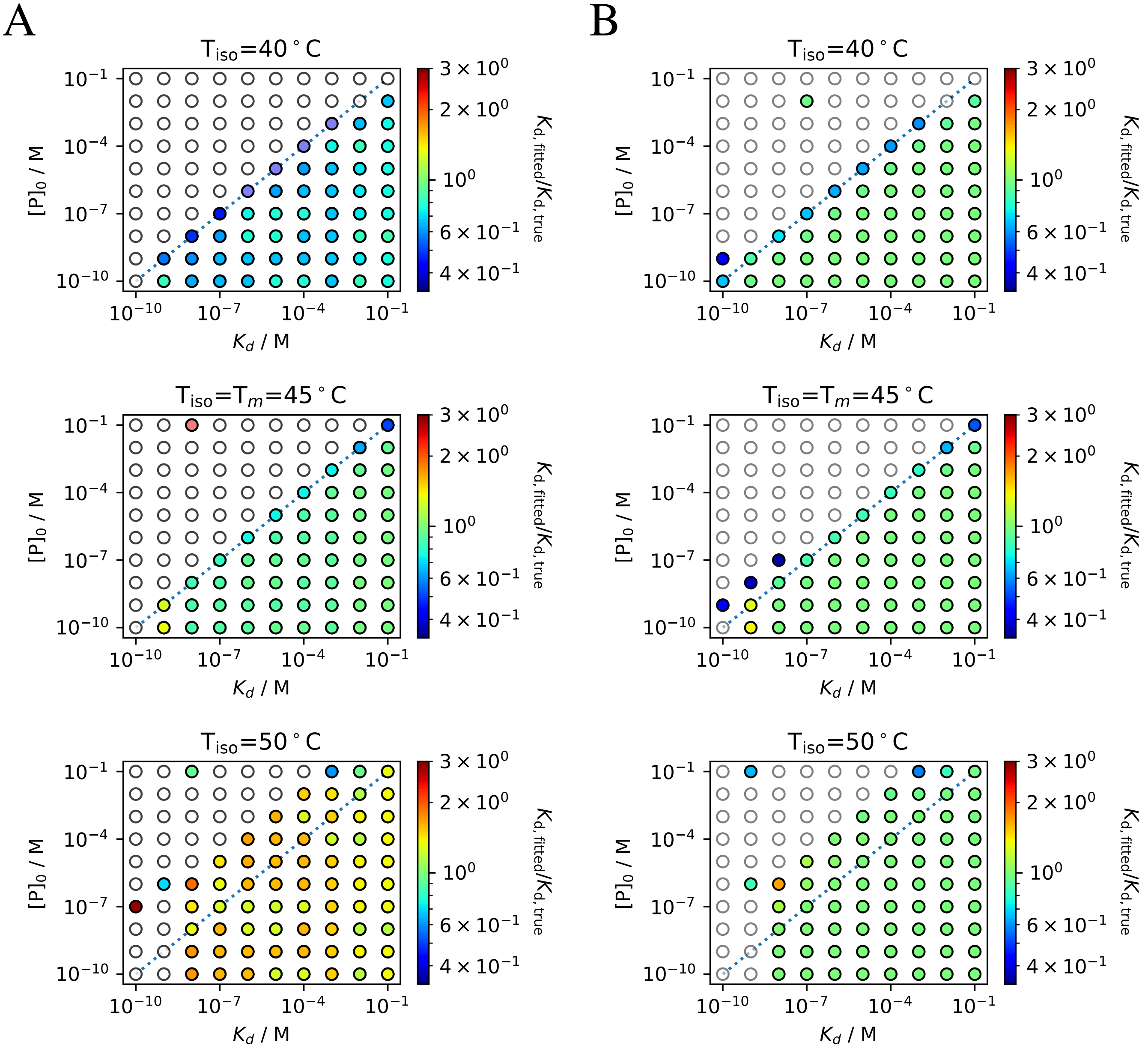

Figure S3. K_d fits for simulated data with $\Delta C_p = 4\,\text{kcal/molK}$ and 0% noise. K_d deviations are color coded with green corresponding to the initial value. Datasets outside the range are shown as white circles. Fitting the ΔC_p during the thermal curve fitting process results in better agreement with the intial K_d values (B) compared to fits with a fixed $\Delta C_p = 0\,\text{kcal/molK}$ (A).

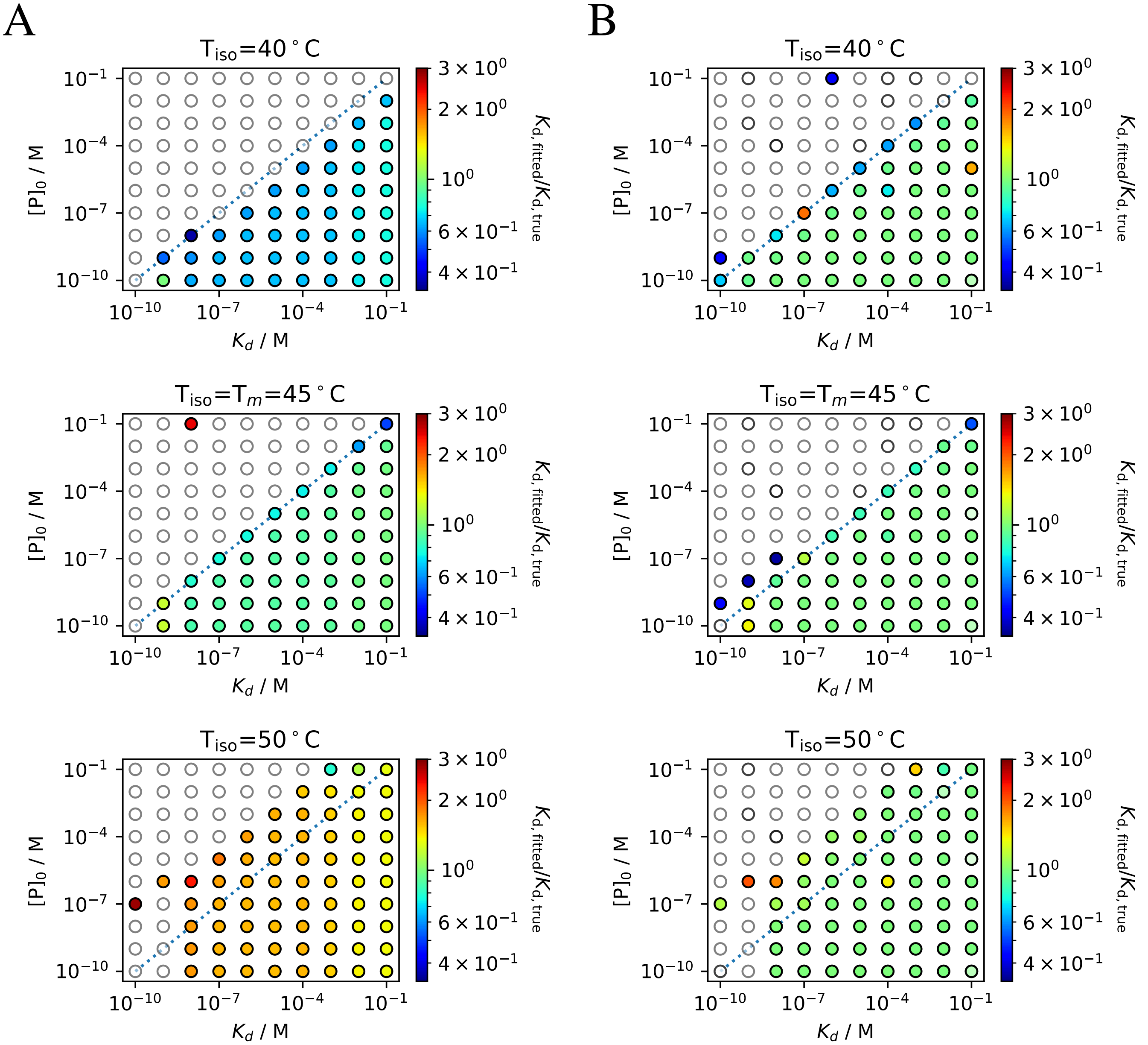

Figure S4. K_d fits for simulated data with $\Delta C_p = 8\,\mathrm{kcal/molK}$ and 0% noise. K_d deviations are color coded with green corresponding to the initial value. Datasets outside the range are shown as white circles. Fitting the ΔC_p during the thermal curve fitting process results in better agreement with the intial K_d values (B) compared to fits with a fixed $\Delta C_p = 0\,\mathrm{kcal/molK}$ (A).

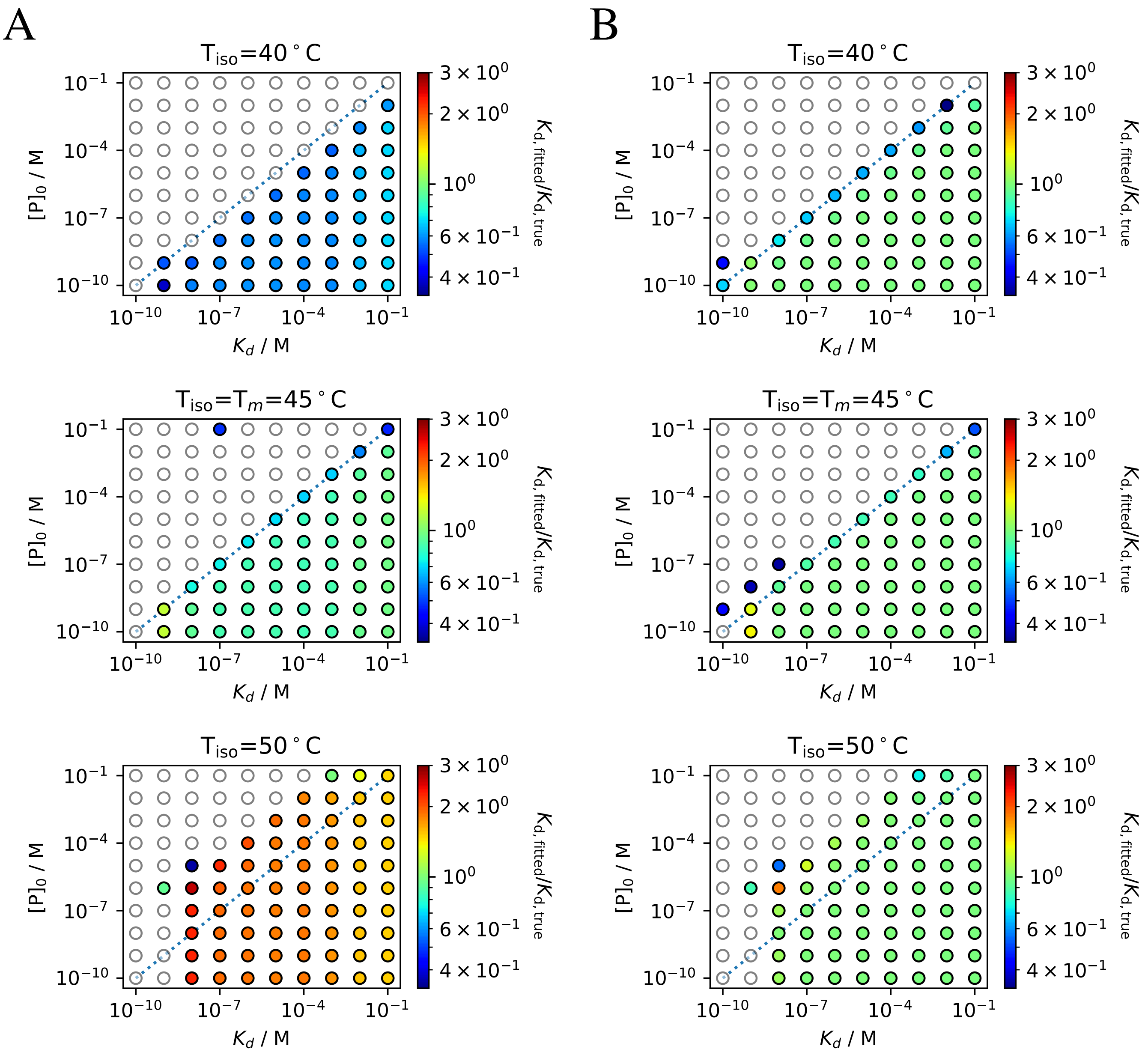

Figure S5. K_d fits for simulated data with $\Delta C_p = 12\,\text{kcal/molK}$ and 0% noise. K_d deviations are color coded with green corresponding to the initial value. Datasets outside the range are shown as white circles. Fitting the ΔC_p during the thermal curve fitting process results in better agreement with the intial K_d values (B) compared to fits with a fixed $\Delta C_p = 0\,\text{kcal/molK}$ (A).

1.3 K_d fittings with 1% noise

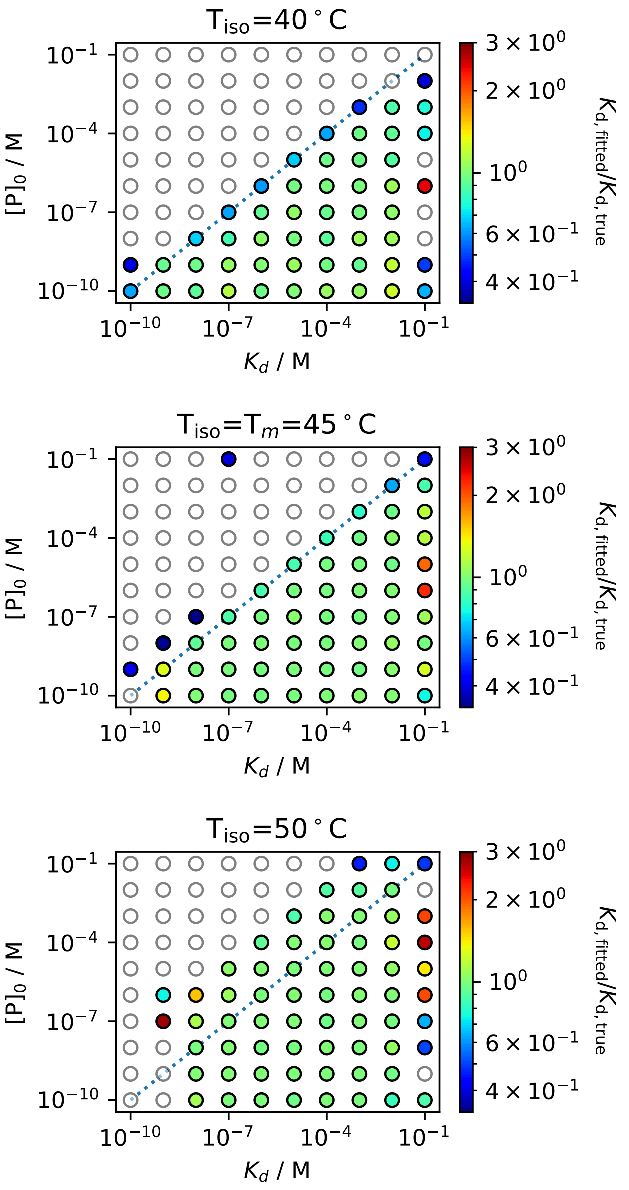

Figure S6. K_d fits for simulated data with $\Delta C_p = 0\,\text{kcal/molK}$ and 1% noise. K_d deviations are color coded with green corresponding to the initial value. Datasets outside the range are shown as white circles. Fitting the ΔC_p during the thermal curve fitting process results in better agreement with the intial K_d values (B) compared to fits with a fixed $\Delta C_p = 0\,\text{kcal/molK}$ (A).

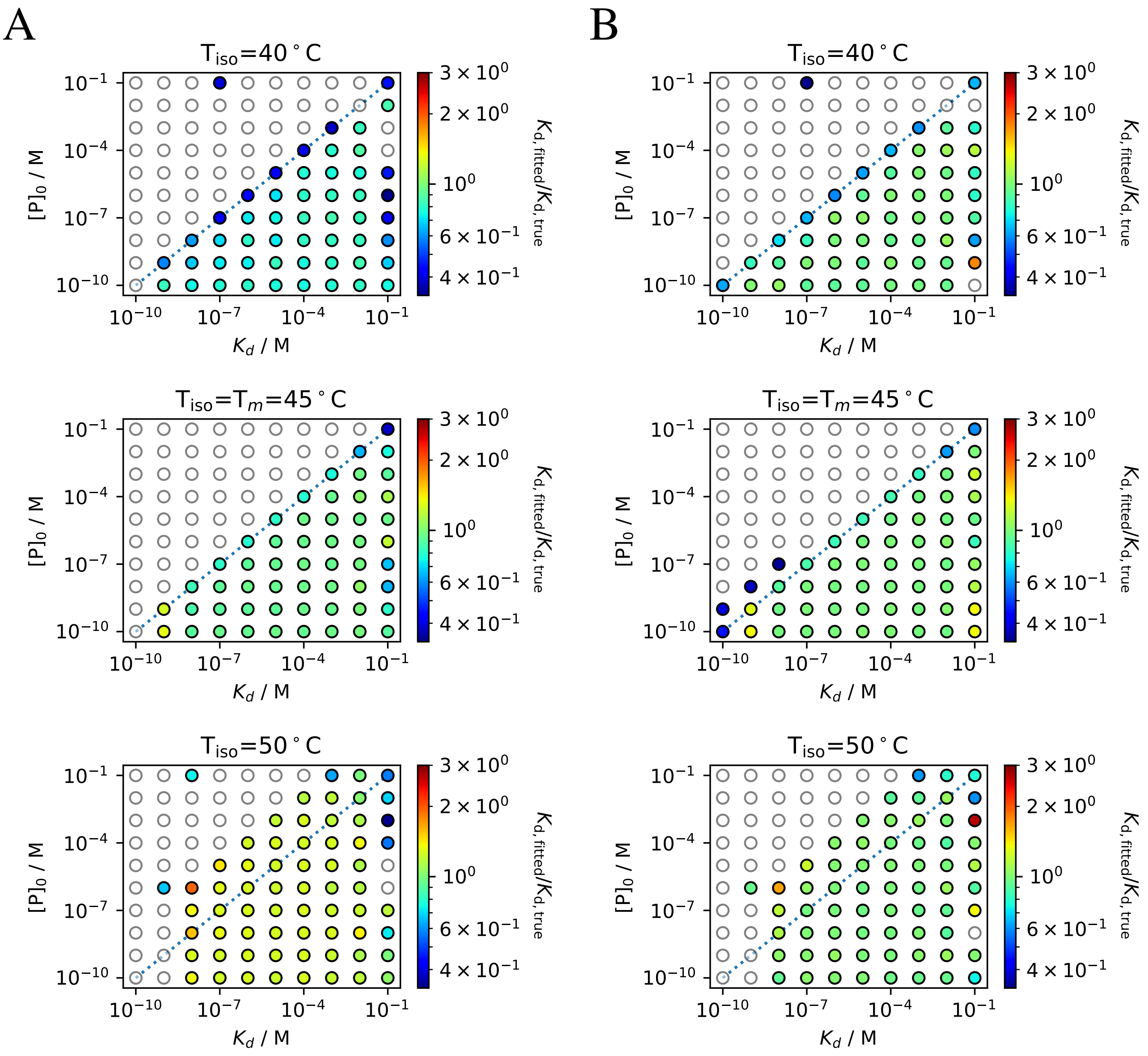

Figure S7. K_d fits for simulated data with $\Delta C_p = 4\,\text{kcal/molK}$ and 1% noise. K_d deviations are color coded with green corresponding to the initial value. Datasets outside the range are shown as white circles. Fitting the ΔC_p during the thermal curve fitting process results in better agreement with the intial K_d values (B) compared to fits with a fixed $\Delta C_p = 0\,\text{kcal/molK}$ (A).

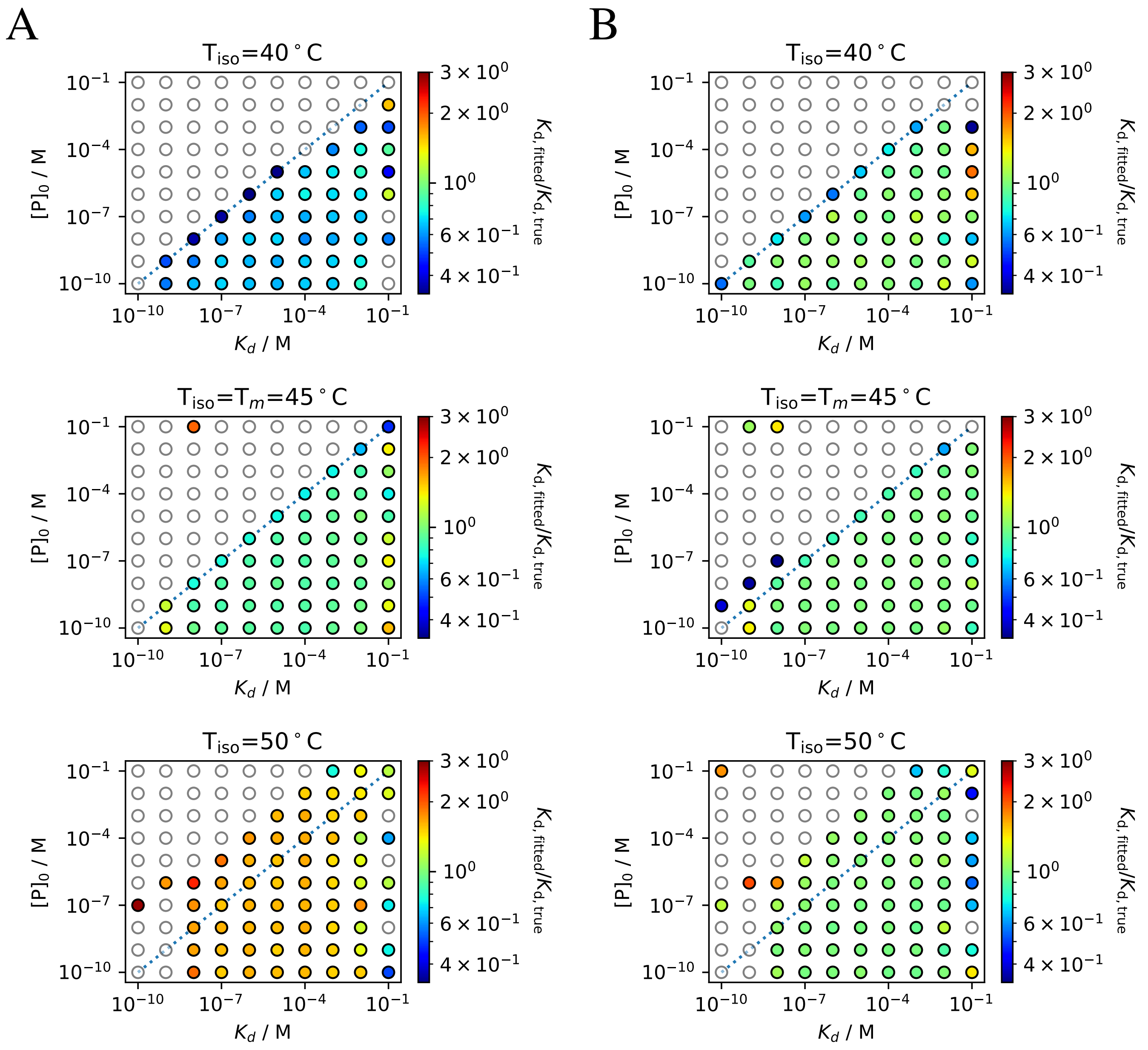

Figure S8. K_d fits for simulated data with $\Delta C_p = 8\,\text{kcal/molK}$ and 1% noise. K_d deviations are color coded with green corresponding to the initial value. Datasets outside the range are shown as white circles. Fitting the ΔC_p during the thermal curve fitting process results in better agreement with the intial K_d values (B) compared to fits with a fixed $\Delta C_p = 0\,\text{kcal/molK}$ (A).

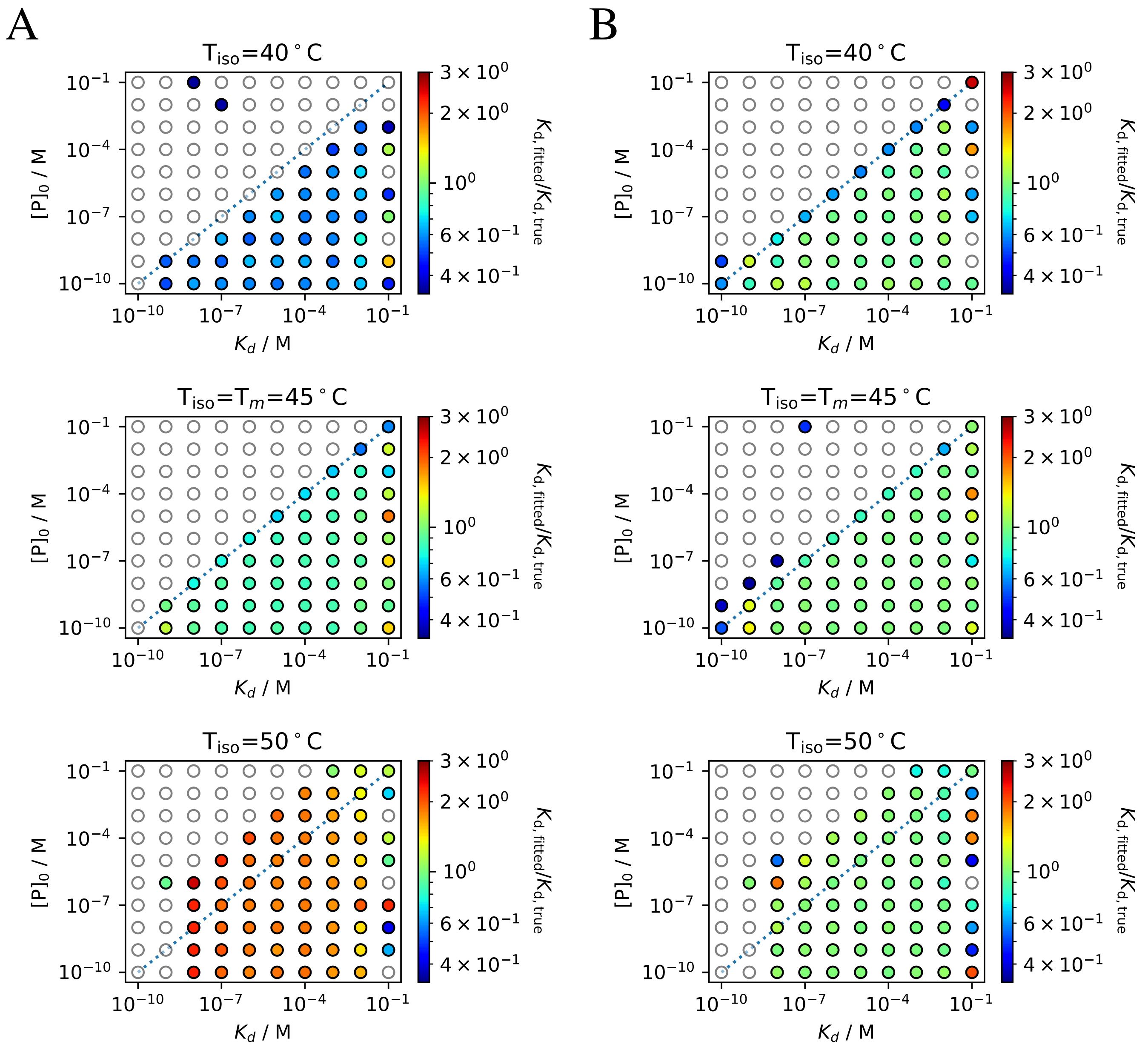

Figure S9. K_d fits for simulated data with $\Delta C_p = 12\,\mathrm{kcal/molK}$ and 1% noise. K_d deviations are color coded with green corresponding to the initial value. Datasets outside the range are shown as white circles. Fitting the ΔC_p during the thermal curve fitting process results in better agreement with the intial K_d values (B) compared to fits with a fixed $\Delta C_p = 0\,\mathrm{kcal/molK}$ (A).

1.4 K_d fittings with 2% noise

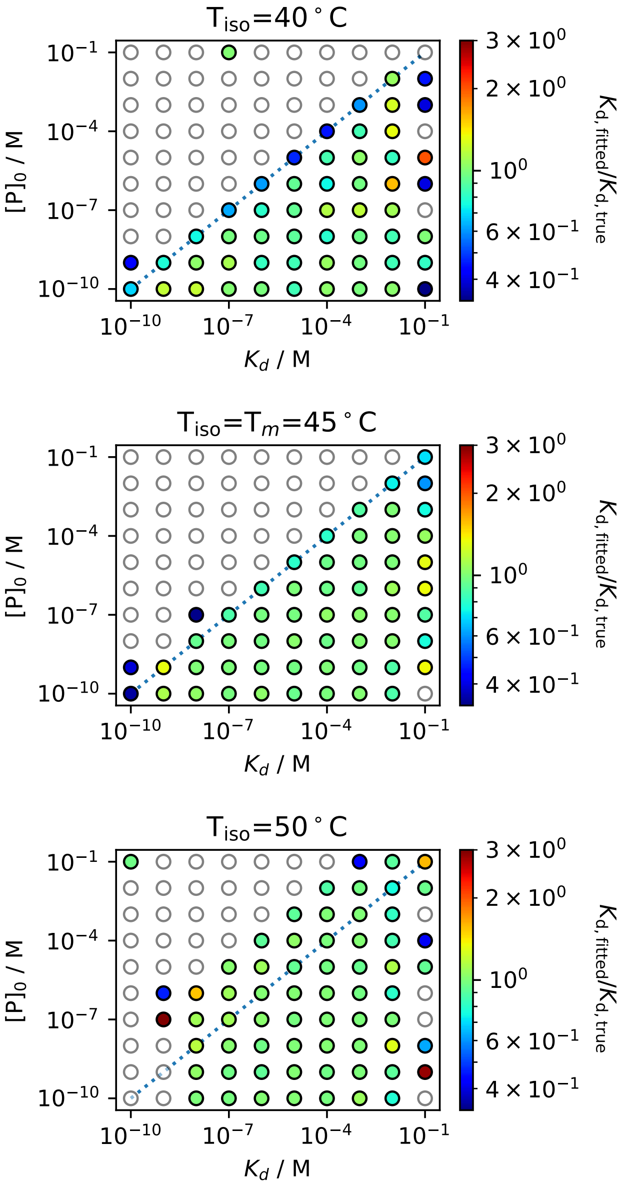

Figure S10. K_d fits for simulated data with $\Delta C_p = 0\,\mathrm{kcal/molK}$ and 2% noise. K_d deviations are color coded with green corresponding to the initial value. Datasets outside the range are shown as white circles. Fitting the ΔC_p during the thermal curve fitting process results in better agreement with the intial K_d values (B) compared to fits with a fixed $\Delta C_p = 0\,\mathrm{kcal/molK}$ (A).

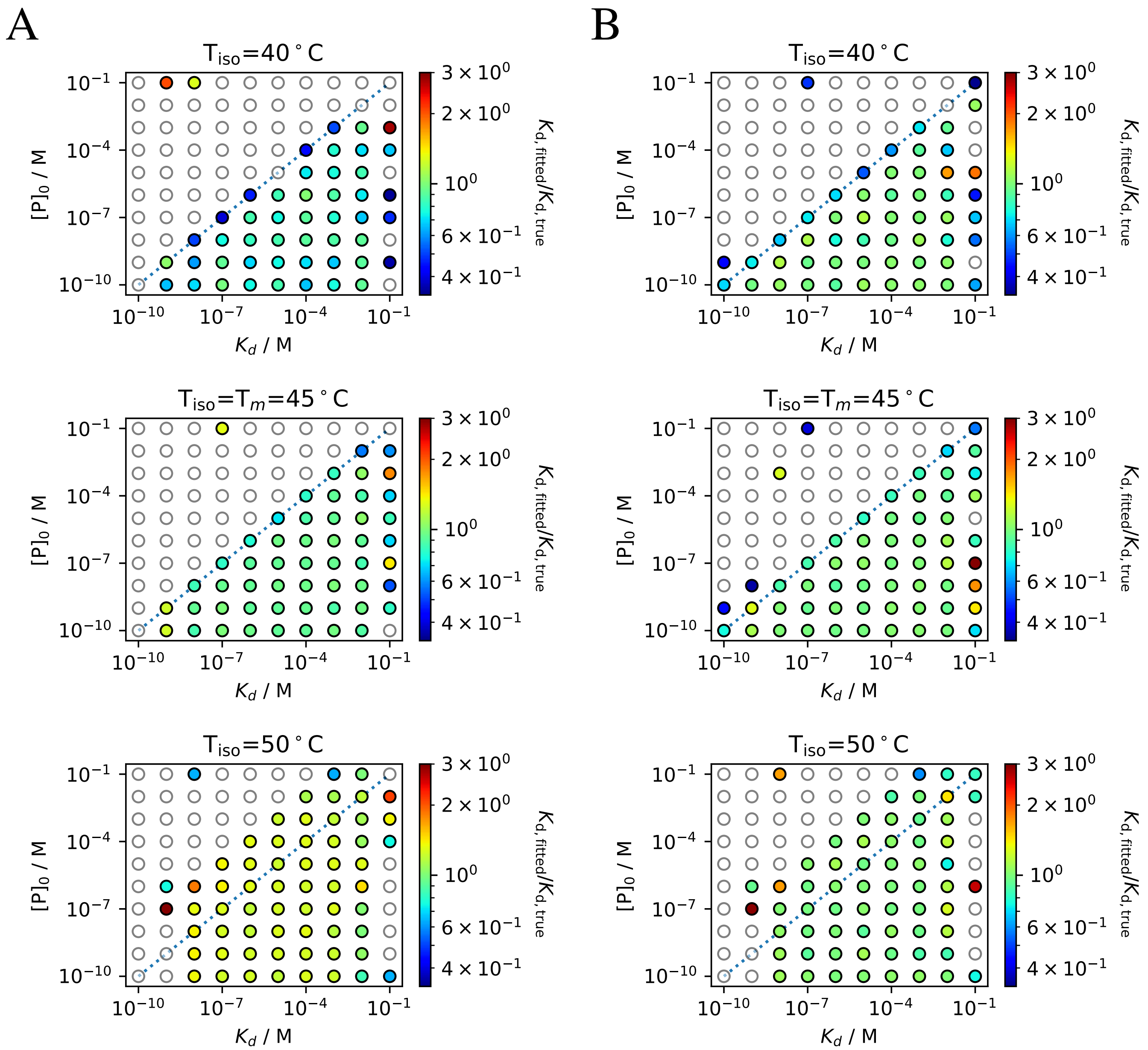

Figure S11. K_d fits for simulated data with $\Delta C_p = 4\,\mathrm{kcal/molK}$ and 2% noise. K_d deviations are color coded with green corresponding to the initial value. Datasets outside the range are shown as white circles. Fitting the ΔC_p during the thermal curve fitting process results in better agreement with the intial K_d values (B) compared to fits with a fixed $\Delta C_p = 0\,\mathrm{kcal/molK}$ (A).

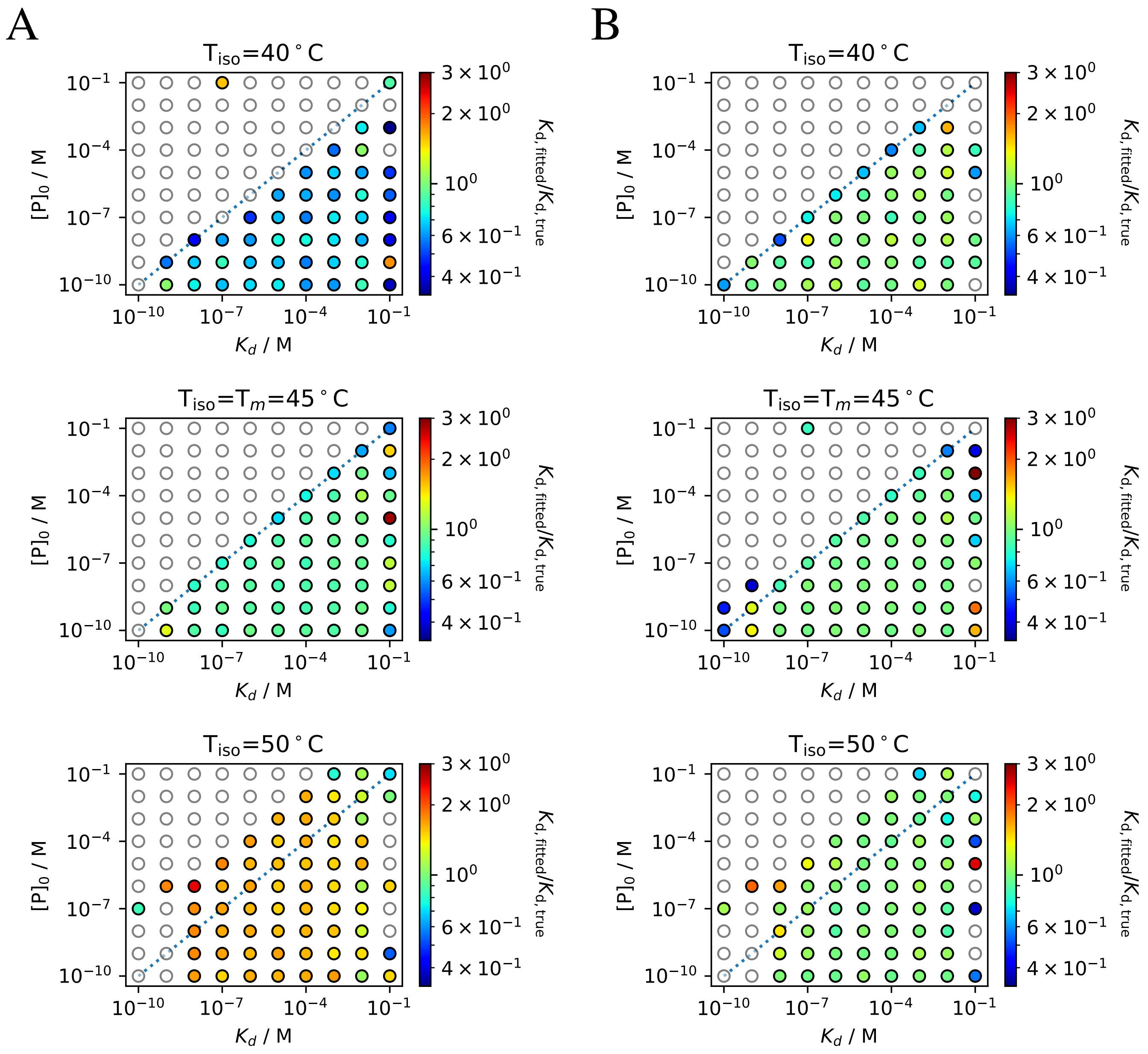

Figure S12. K_d fits for simulated data with $\Delta C_p = 8\,$kcal/molK and 2% noise. K_d deviations are color coded with green corresponding to the initial value. Datasets outside the range are shown as white circles. Fitting the ΔC_p during the thermal curve fitting process results in better agreement with the intial K_d values (B) compared to fits with a fixed $\Delta C_p = 0\,$kcal/molK (A). Datasets outside the range are shown as white circles.

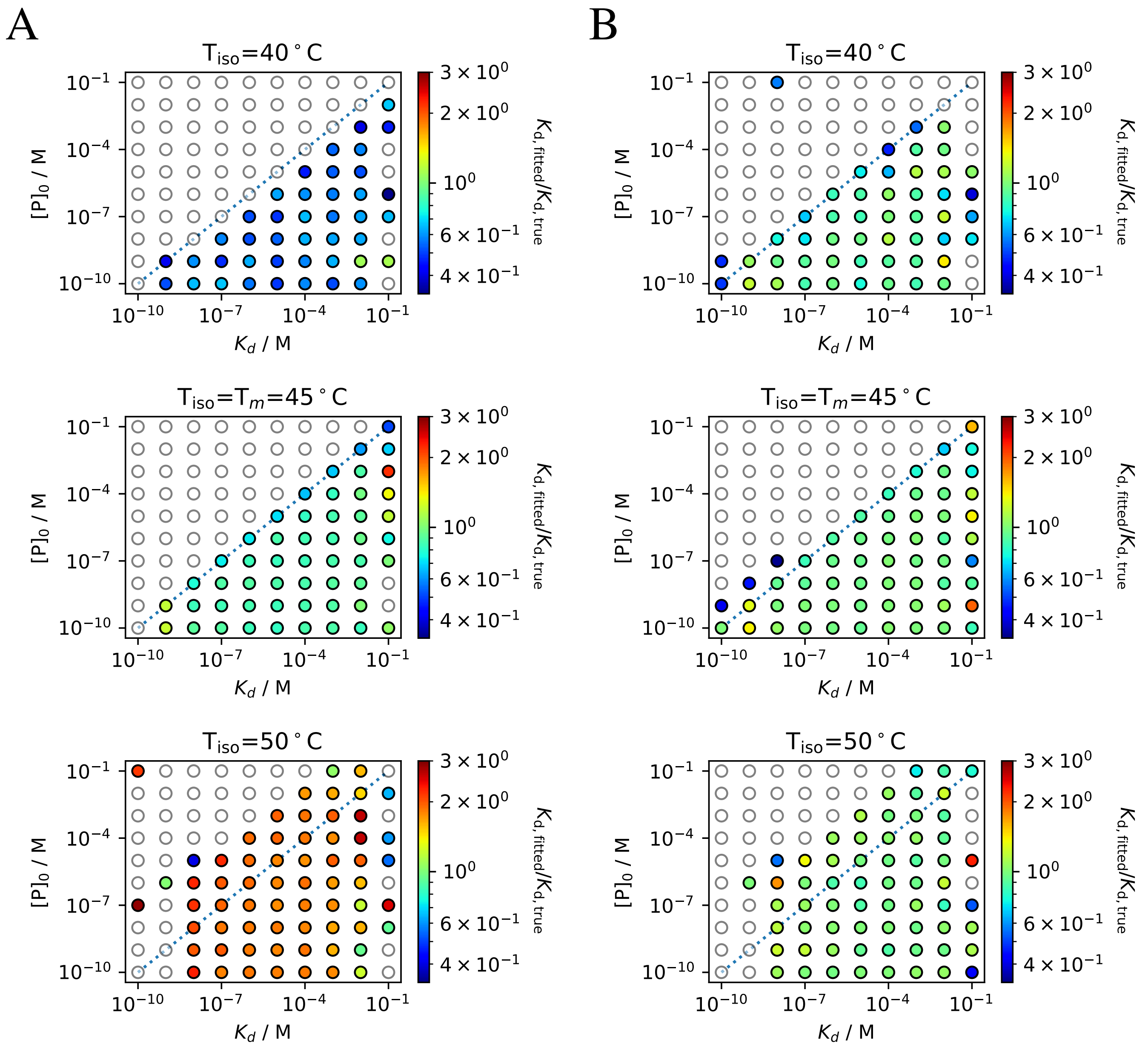

Figure S13. K_d fits for simulated data with $\Delta C_p = 12\,\mathrm{kcal/molK}$ and 2% noise. K_d deviations are color coded with green corresponding to the initial value. Datasets outside the range are shown as white circles. Fitting the ΔC_p during the thermal curve fitting process results in better agreement with the intial K_d values (B) compared to fits with a fixed $\Delta C_p = 0\,\mathrm{kcal/molK}$ (A).

1.5 K_d fittings with 5% noise

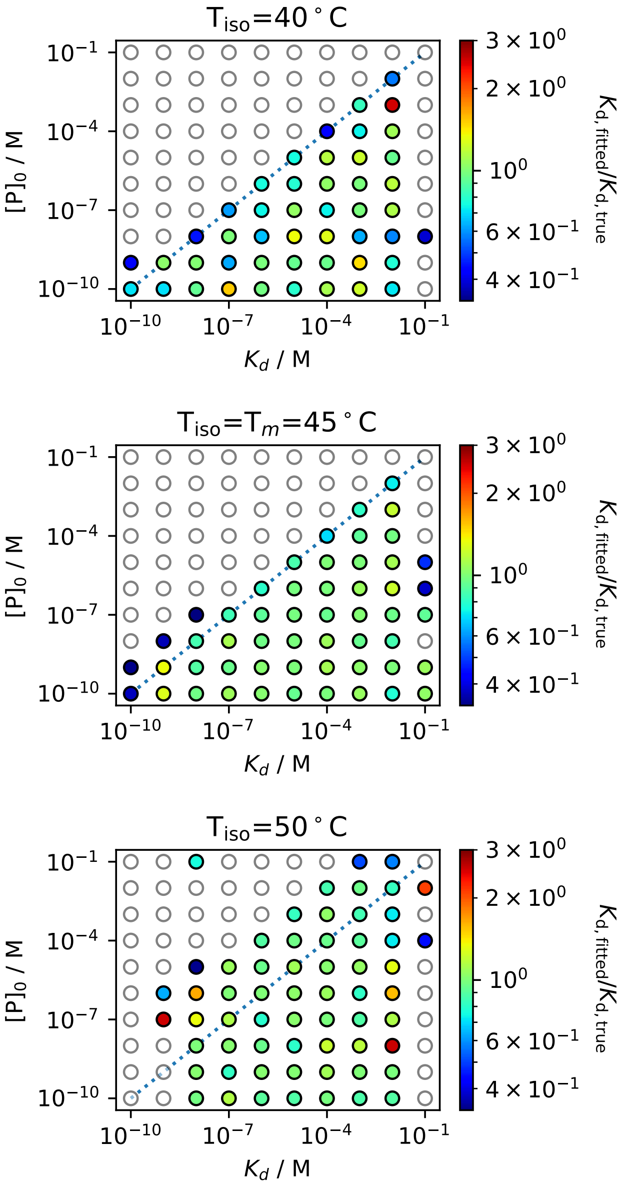

Figure S14. K_d fits for simulated data with $\Delta C_p = 0$ kcal/molK and 5% noise. K_d deviations are color coded with green corresponding to the initial value. Datasets outside the range are shown as white circles. Fitting the ΔC_p during the thermal curve fitting process results in better agreement with the intial K_d values (B) compared to fits with a fixed $\Delta C_p = 0$ kcal/molK (A).

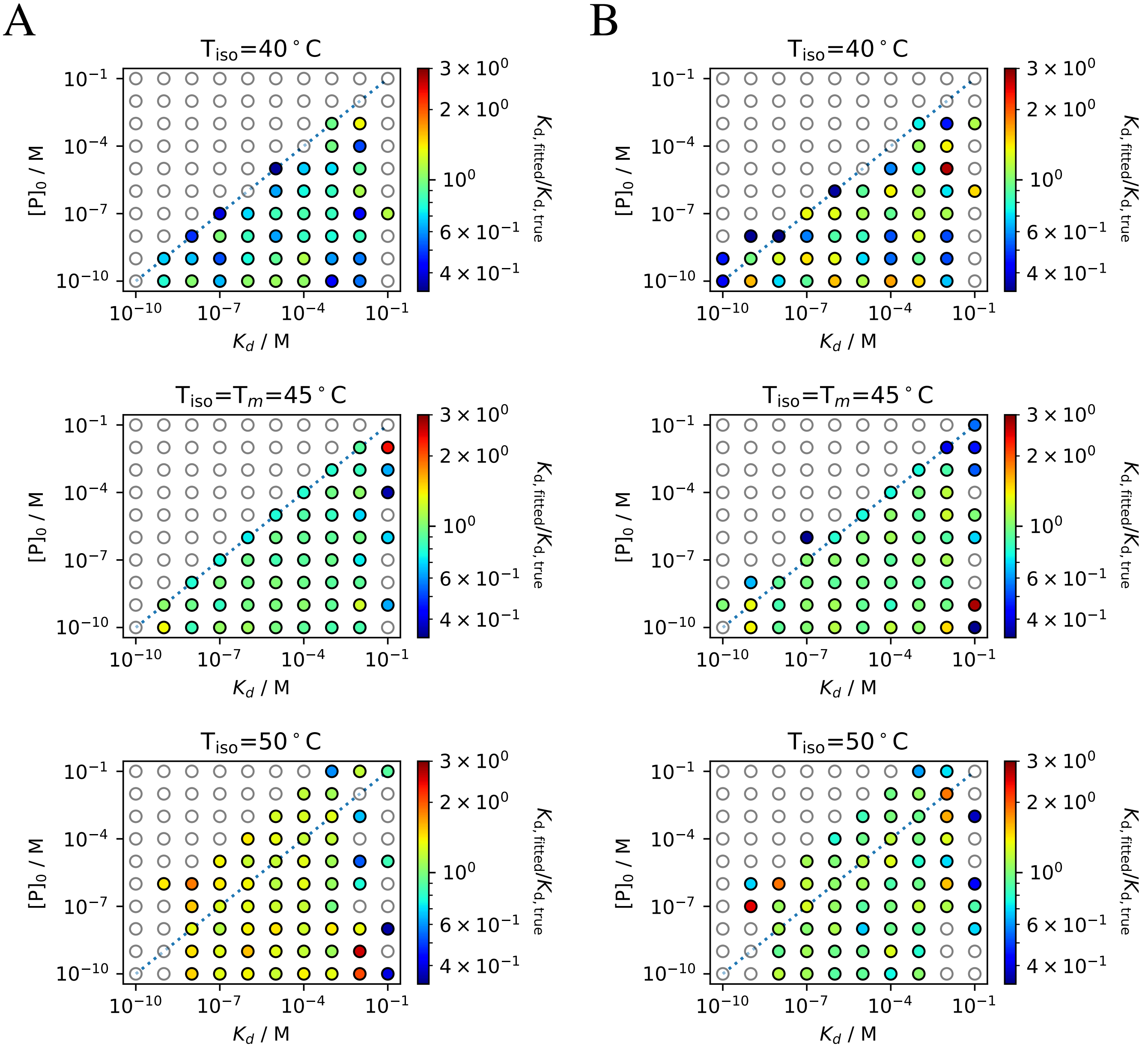

Figure S15. K_d fits for simulated data with $\Delta C_p = 4$ kcal/molK and 5% noise. K_d deviations are color coded with green corresponding to the initial value. Datasets outside the range are shown as white circles. Fitting the ΔC_p during the thermal curve fitting process results in better agreement with the intial K_d values (B) compared to fits with a fixed $\Delta C_p = 0$ kcal/molK (A).

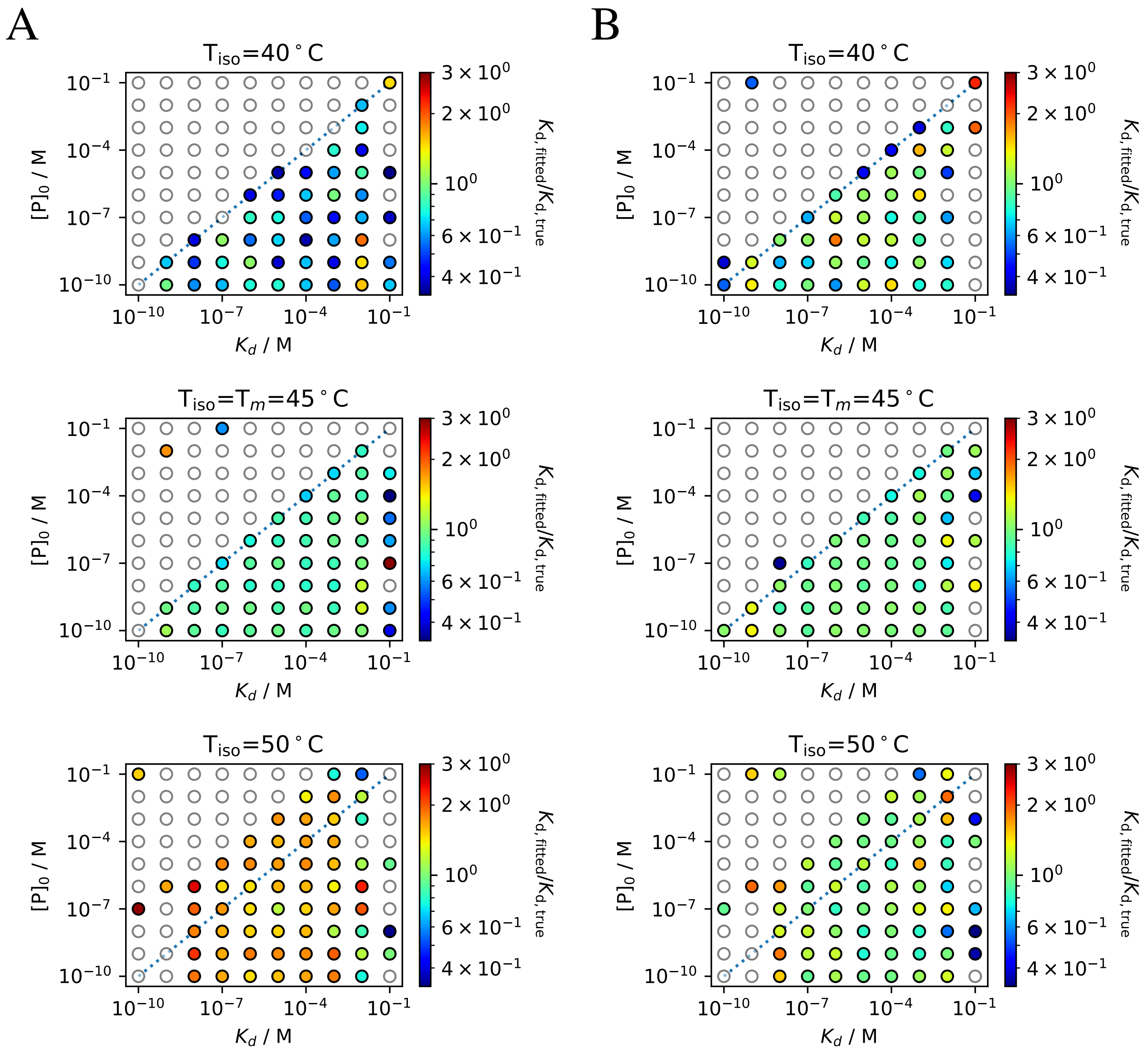

Figure S16. K_d fits for simulated data with $\Delta C_p = 8$ kcal/molK and 5% noise. K_d deviations are color coded with green corresponding to the initial value. Datasets outside the range are shown as white circles. Fitting the ΔC_p during the thermal curve fitting process results in better agreement with the intial K_d values (B) compared to fits with a fixed $\Delta C_p = 0$ kcal/molK (A).

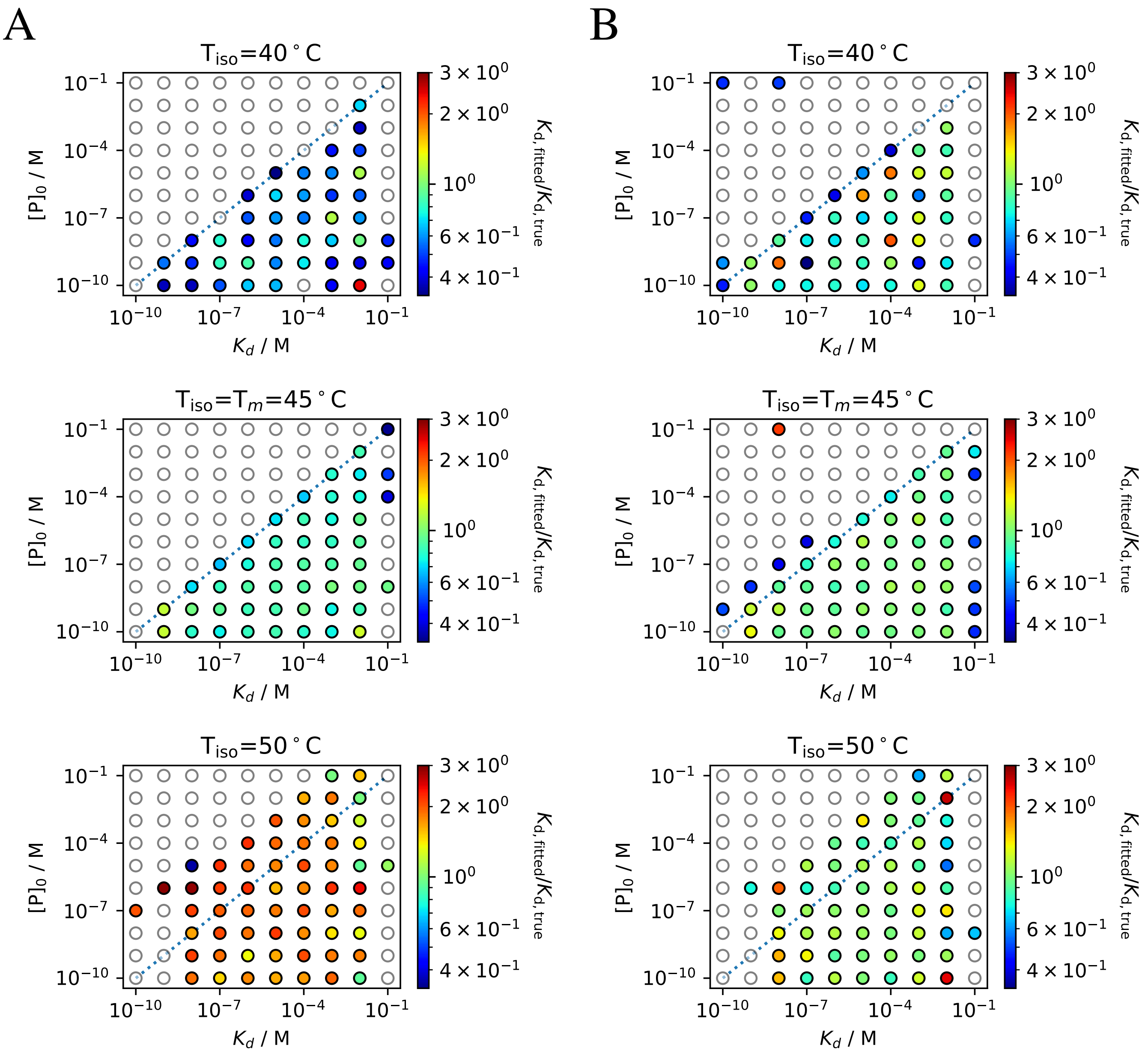

Figure S17. K_d fits for simulated data with $\Delta C_p = 12\,\mathrm{kcal/molK}$ and 5% noise. K_d deviations are color coded with green corresponding to the initial value. Datasets outside the range are shown as white circles. Fitting the ΔC_p during the thermal curve fitting process results in better agreement with the intial K_d values (B) compared to fits with a fixed $\Delta C_p = 0\,\mathrm{kcal/molK}$ (A).

1.6 K_d fittings with 10% noise

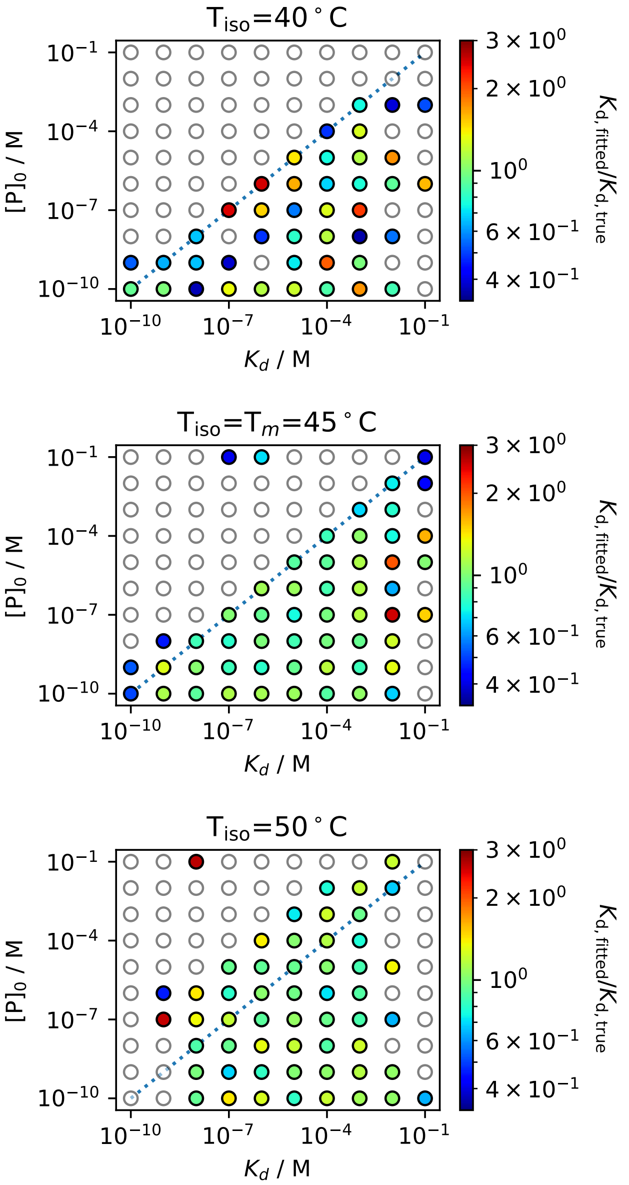

Figure S18. K_d fits for simulated data with $\Delta C_p = 0\,\mathrm{kcal/molK}$ and 10% noise. K_d deviations are color coded with green corresponding to the initial value. Datasets outside the range are shown as white circles. Fitting the ΔC_p during the thermal curve fitting process results in better agreement with the intial K_d values (B) compared to fits with a fixed $\Delta C_p = 0\,\mathrm{kcal/molK}$ (A).

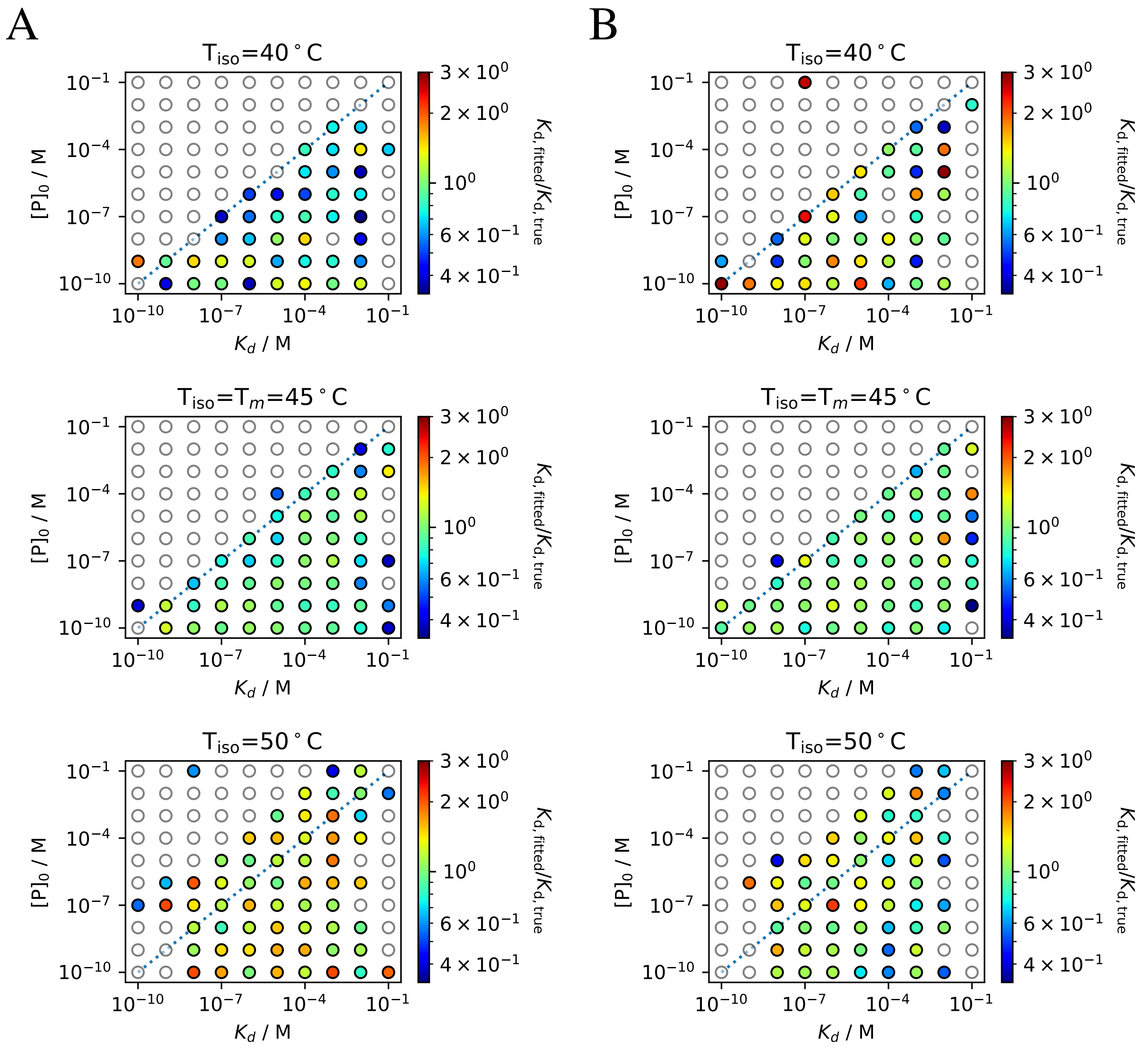

Figure S19. K_d fits for simulated data with $\Delta C_p = 4$ kcal/molK and 10% noise. K_d deviations are color coded with green corresponding to the initial value. Datasets outside the range are shown as white circles. Fitting the ΔC_p during the thermal curve fitting process results in better agreement with the intial K_d values (B) compared to fits with a fixed $\Delta C_p = 0$ kcal/molK (A).

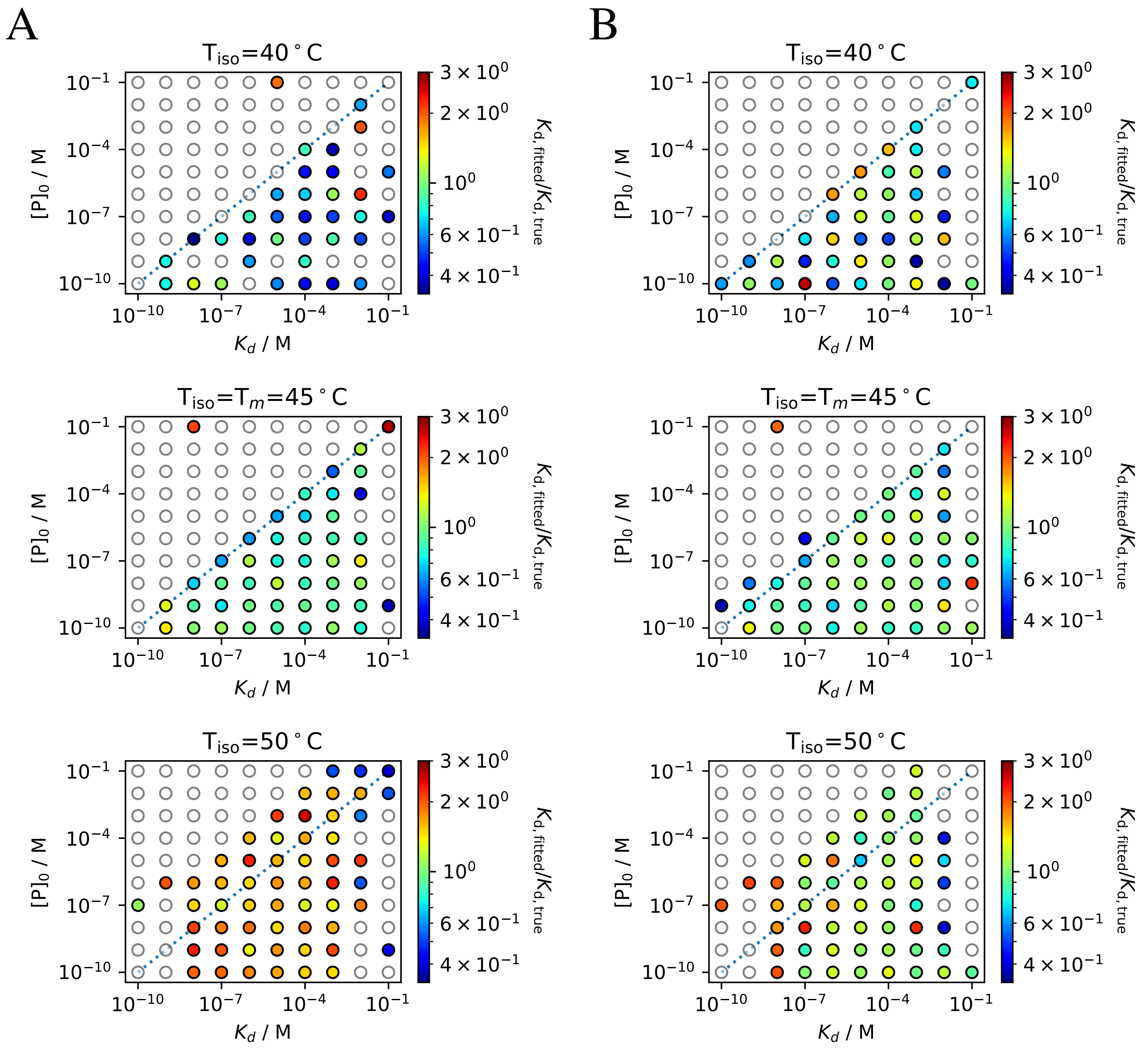

Figure S20. K_d fits for simulated data with $\Delta C_p = 8\,\text{kcal/molK}$ and 10% noise. K_d deviations are color coded with green corresponding to the initial value. Datasets outside the range are shown as white circles. Fitting the ΔC_p during the thermal curve fitting process results in better agreement with the intial K_d values (B) compared to fits with a fixed $\Delta C_p = 0\,\text{kcal/molK}$ (A).

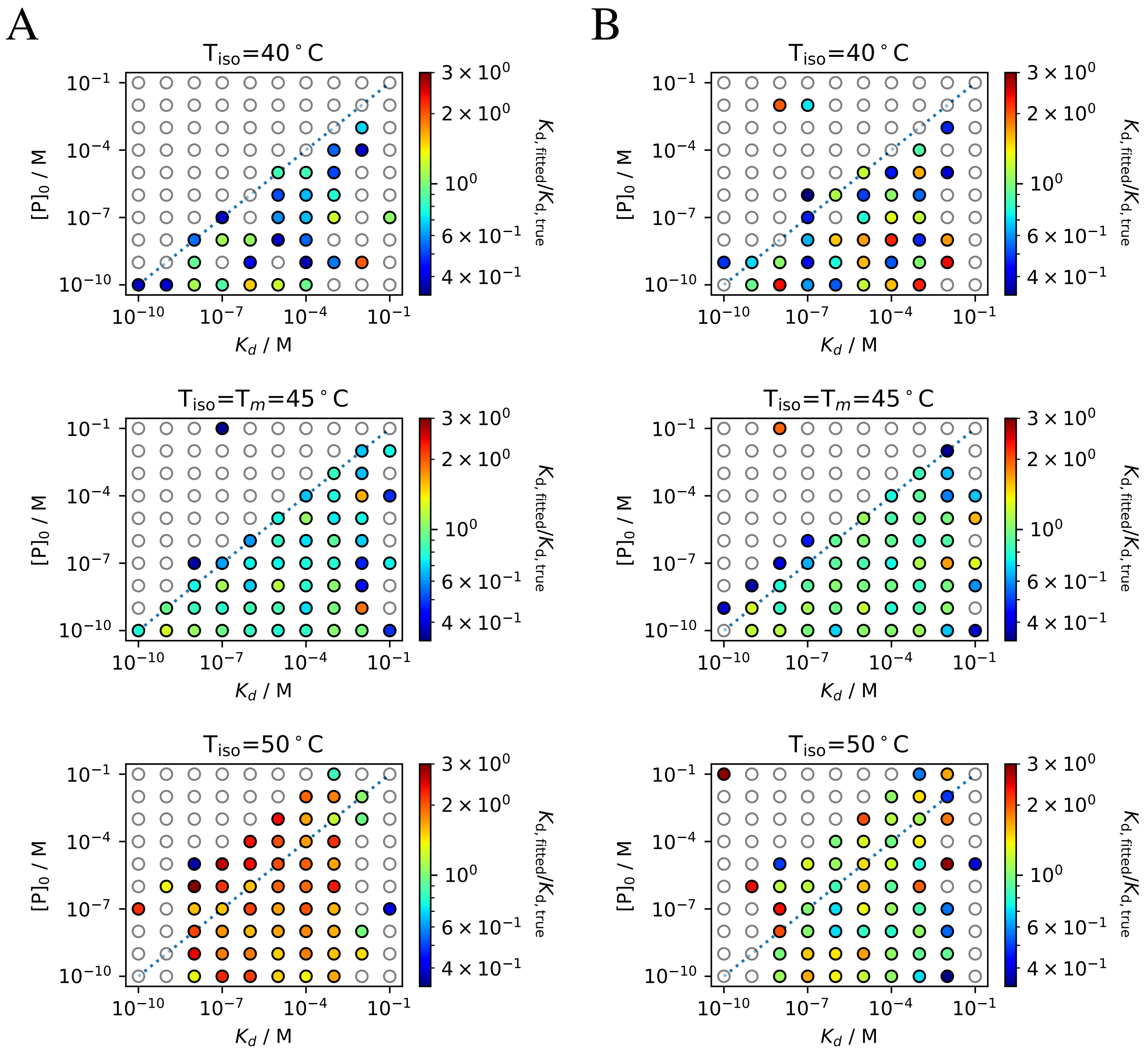

Figure S21. K_d fits for simulated data with $\Delta C_p = 12\,$kcal/molK and 10% noise. K_d deviations are color coded with green corresponding to the initial value. Datasets outside the range are shown as white circles. Fitting the ΔC_p during the thermal curve fitting process results in better agreement with the intial K_d values (B) compared to fits with a fixed $\Delta C_p = 0\,$kcal/molK (A).

1.7 Fitted ΔC_p values

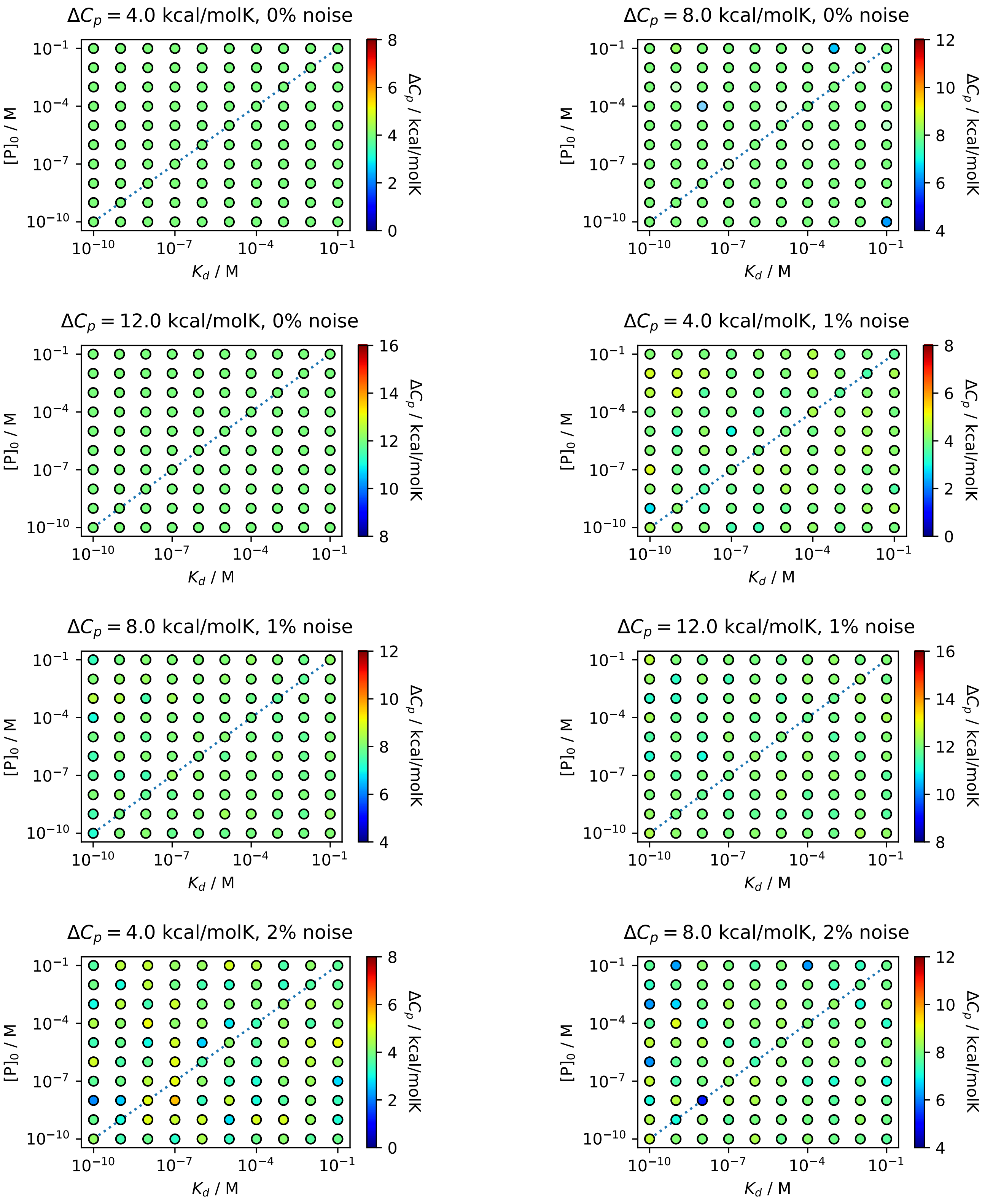

Figure S22. Fitted ΔC_p values for simulated data with different initial ΔC_p noise levels. ΔC_p deviations are color coded with green corresponding to the initial value. Datasets outside the range are shown as white circles. Continued in next figure.

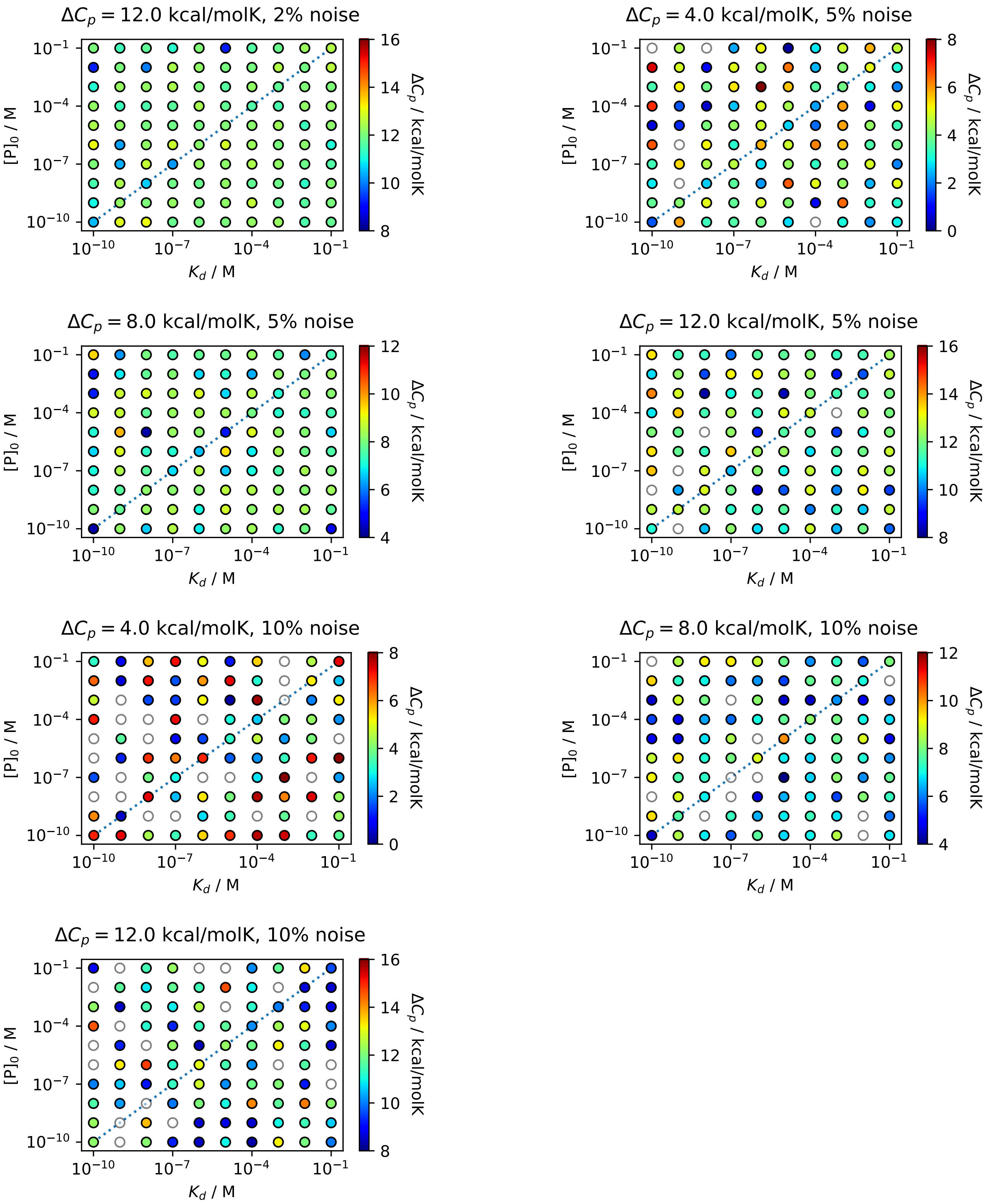

Figure S23. Continuation: Fitted ΔC_p values for simulated data with different initial ΔC_p noise levels. ΔC_p deviations are color coded with green corresponding to the initial value. Datasets outside the range are shown as white circles.

2 Differential scanning calorimetry of Pcs60

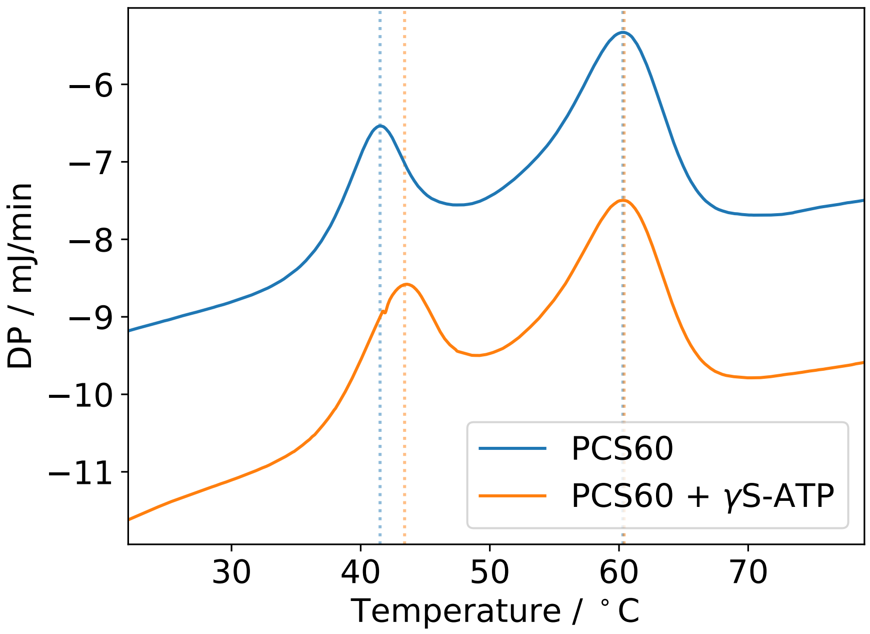

Figure S24. Differential scanning calorimetry (DSC) of Pcs60 (180 μM) in absence and presence of its ligand γS-ATP (192 μM). Here the raw data is shown before baseline correction. Pcs60 shows two transitions between 20 and 80 °C. The temperature of the first transition is shifted by ligand binding from 41.5 to 43.4 °C. The second transition at 60.3 °C is not affected upon ligand binding. Since the two transitions overlap, it was not possible to extract a reliable value of the heat capacity change upon unfolding ΔC_p.

3 Isothermal analyses with fitted ΔC_p for proof-of-principle studies (S25–S28)

3.1 EG1/ADPR

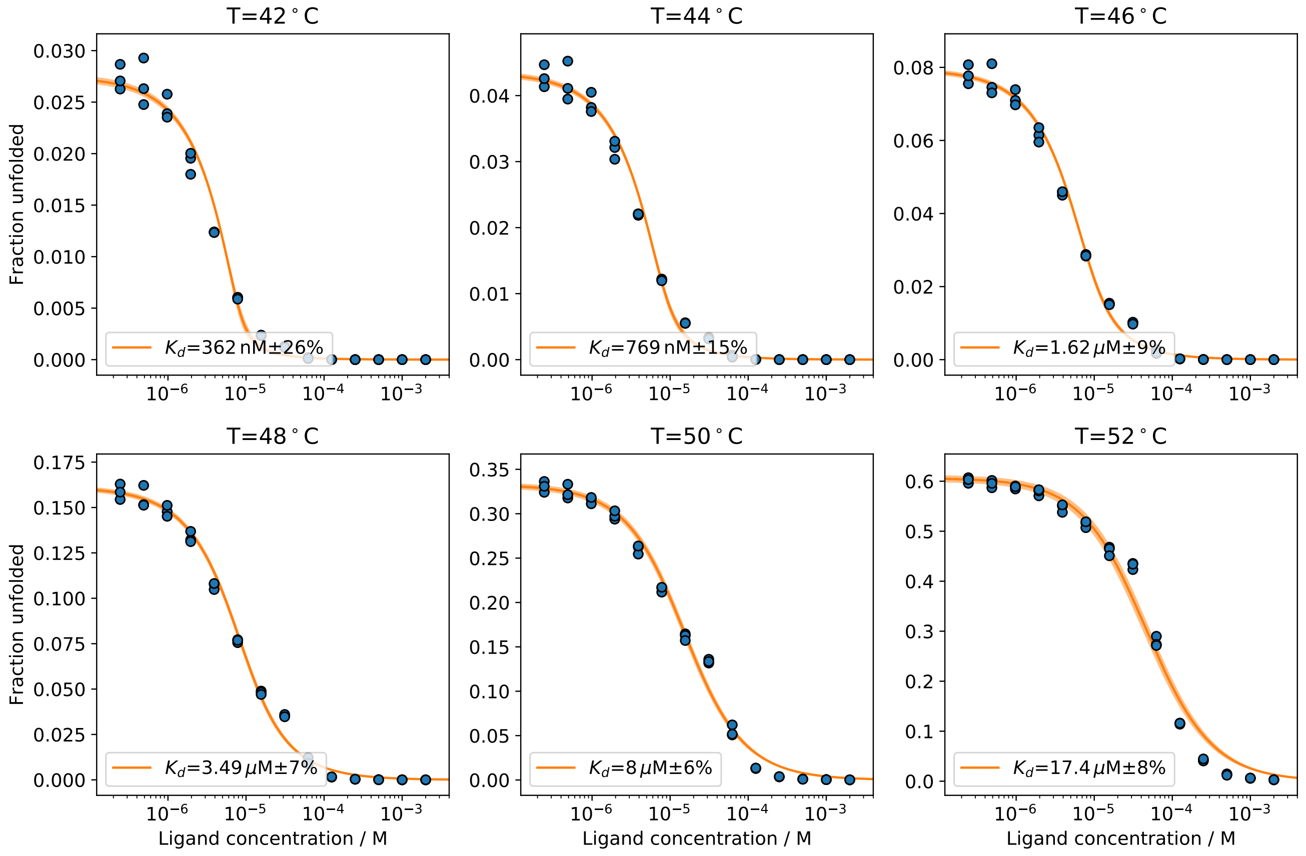

Figure S25. Isothermal analysis for nDSF data shown in fig. 3 for a fitted $\Delta C_p = 8.8$ kcal/molK (cf. table 1 in main text).

3.2 SS1/ADPR

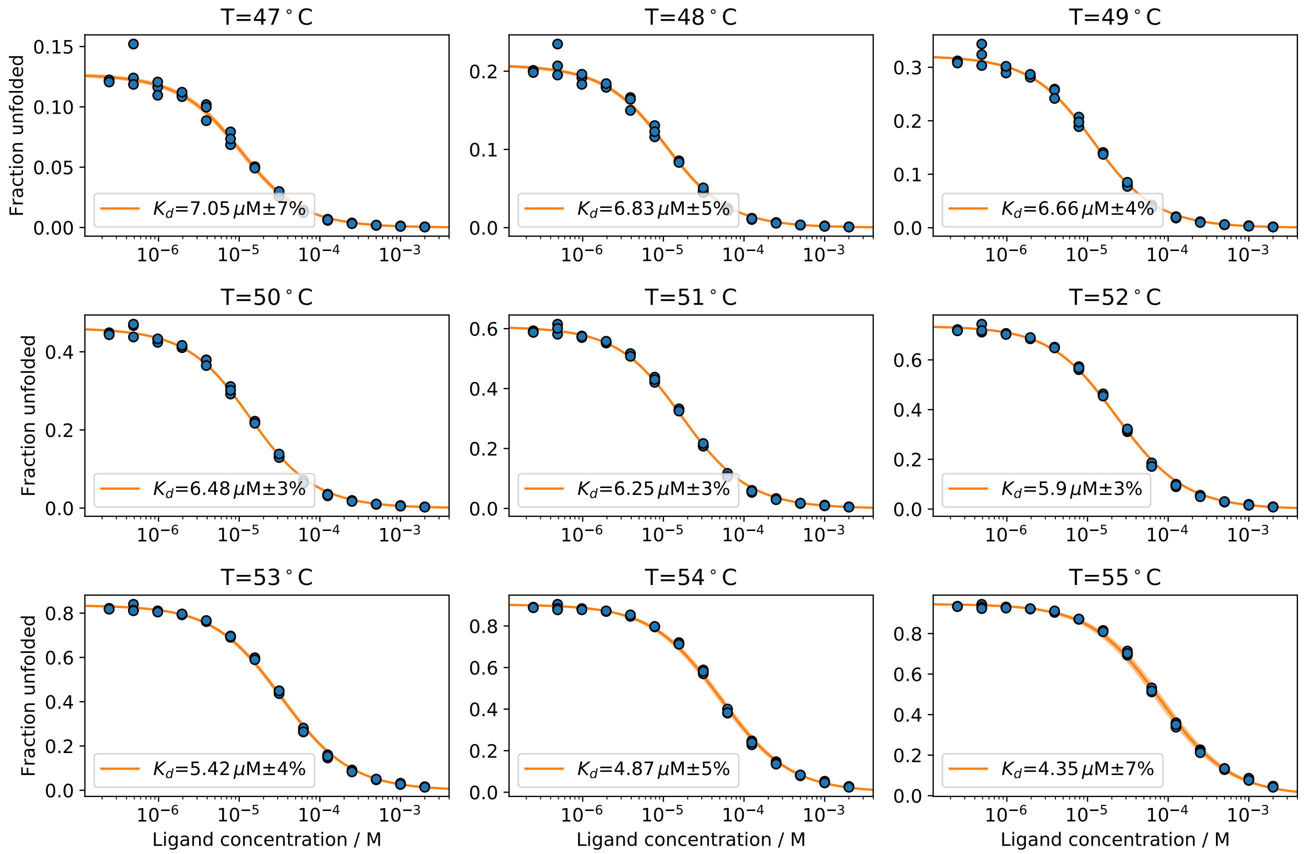

Figure S26. Isothermal analysis for data shown in fig. 4 for a fitted $\Delta C_p = 7.6$kcal/molK (cf. table 1 in main text).

3.3 MHC/NT8

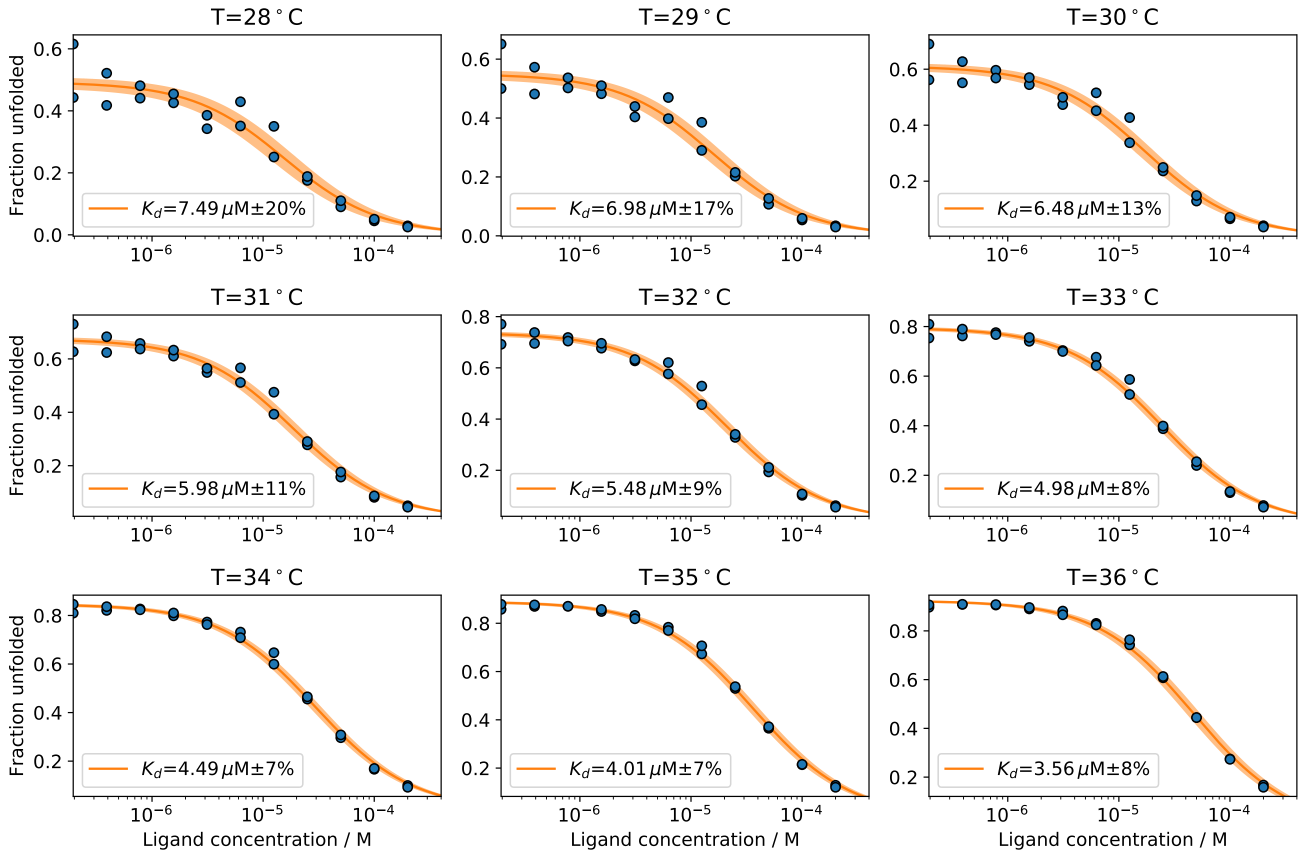

Figure S27. Isothermal analysis for data shown in fig. 5 for a fitted $\Delta C_p = 4.0$kcal/molK (cf. table 1 in main text).

3.4 Pcs60/γS-ATP

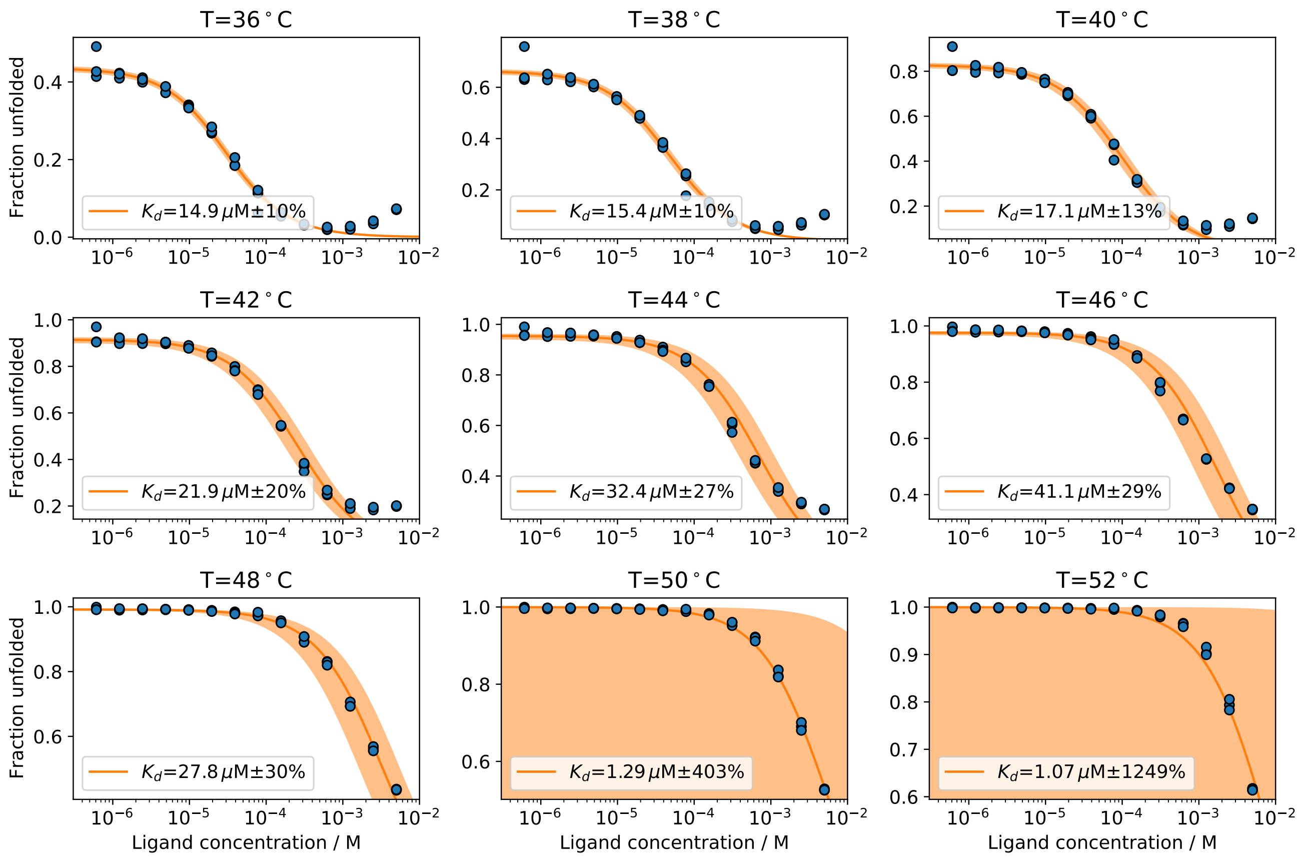

Figure S28. nDSF binding study for Pcs60/γS-ATP (top) and isothermal analysis (bottom) for a fitted $\Delta C_p = 6.7$ kcal/molK (cf. table 1).

4 WT Pcs60 in comparison with the Pcs60 mutant used for this manuscript

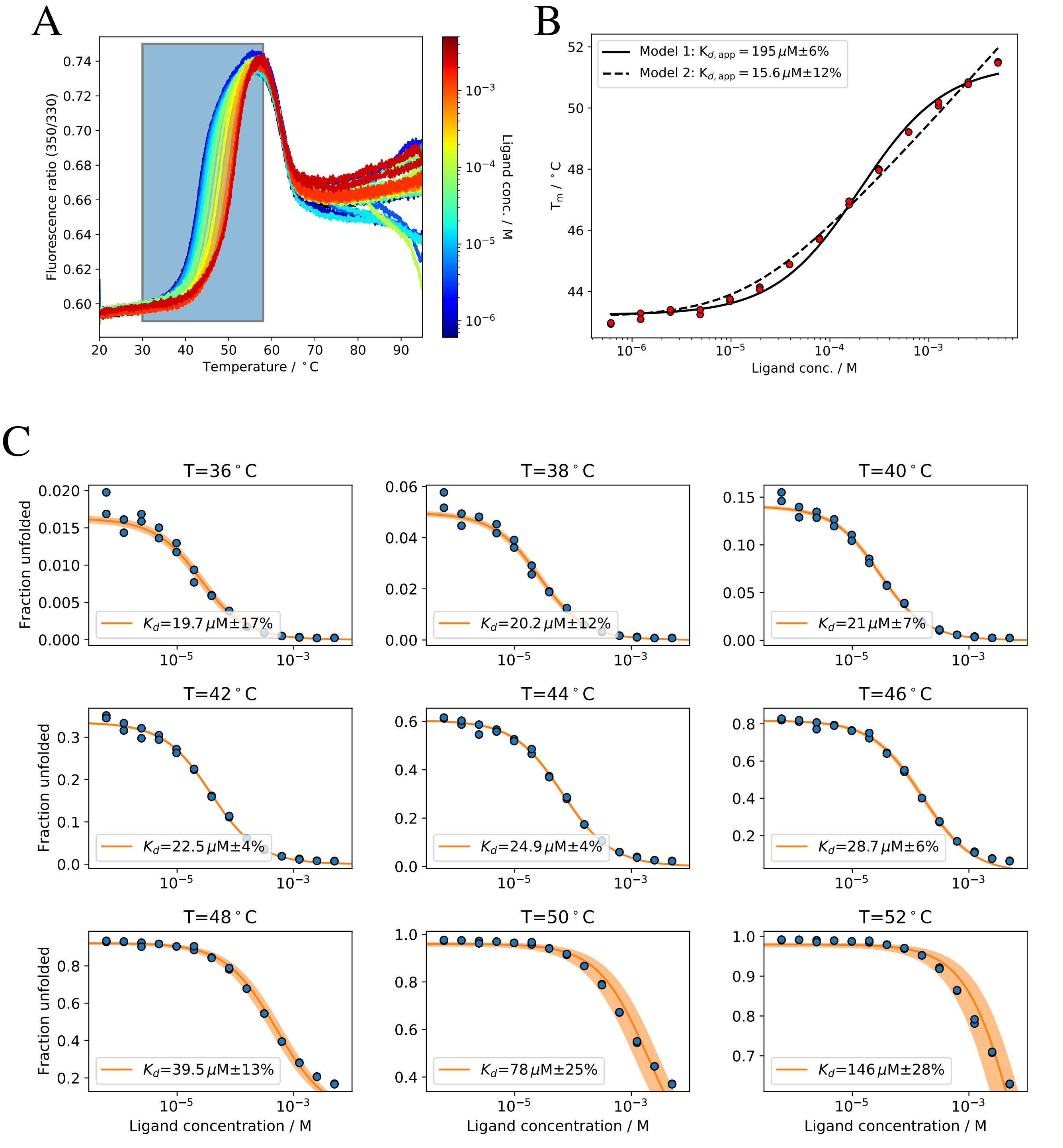

Figure S29. (A) nDSF binding study for wild-type Pcs60 WT/γS-ATP. The region in the colored box was used for isothermal analysis. (B) Melting temperature analysis with two different models yields apparent $K_{d,app}$ values. (C) Isothermal analysis for $\Delta C_p = 0$. Compared to the Pcs60 mutant used for the studies in the main manuscript, the WT shows a very similar binding behaviour with K_d values around 20 μM.

5 Pcs60_K523A mutant

5.1 Sequence alignment between Pcs60 and oxalyl-CoA synthetase from arabidopsis thaliana

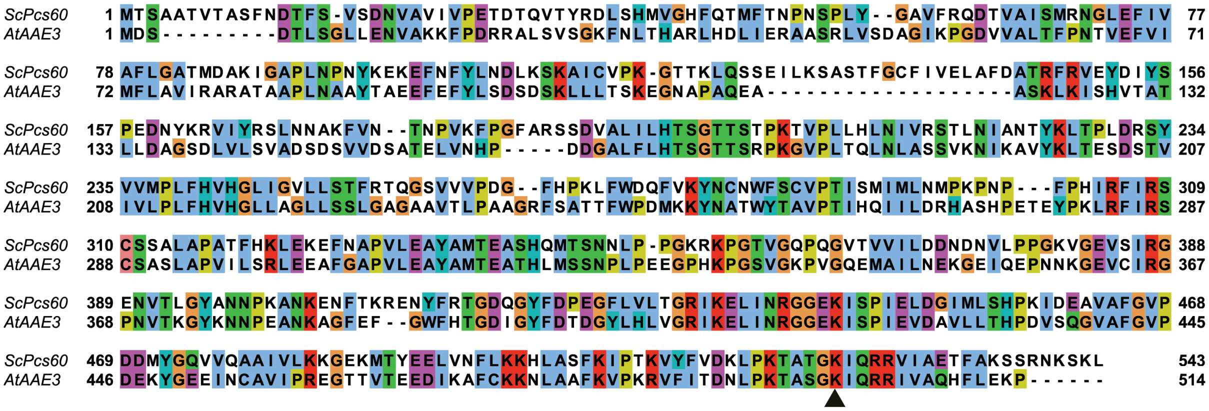

Figure S30. Sequence alignment of Saccharomyces cerevisiae Pcs60 and Arabidopsis thaliana AAE3 oxalyl-CoA synthase. Protein sequences were alignment using MUSCLE (ref.) and visualized using Jalview (ref.). Conserved positively charged residues are in red, negatively charged residues are in magenta, hydrophobic residues in blue, polar residues in green, cysteines in pink, glycines in orange, prolines in yellow aromatic in cyan and non-conserved residues in white.

5.2 Binding study with Pcs60_K523A/γS-ATP

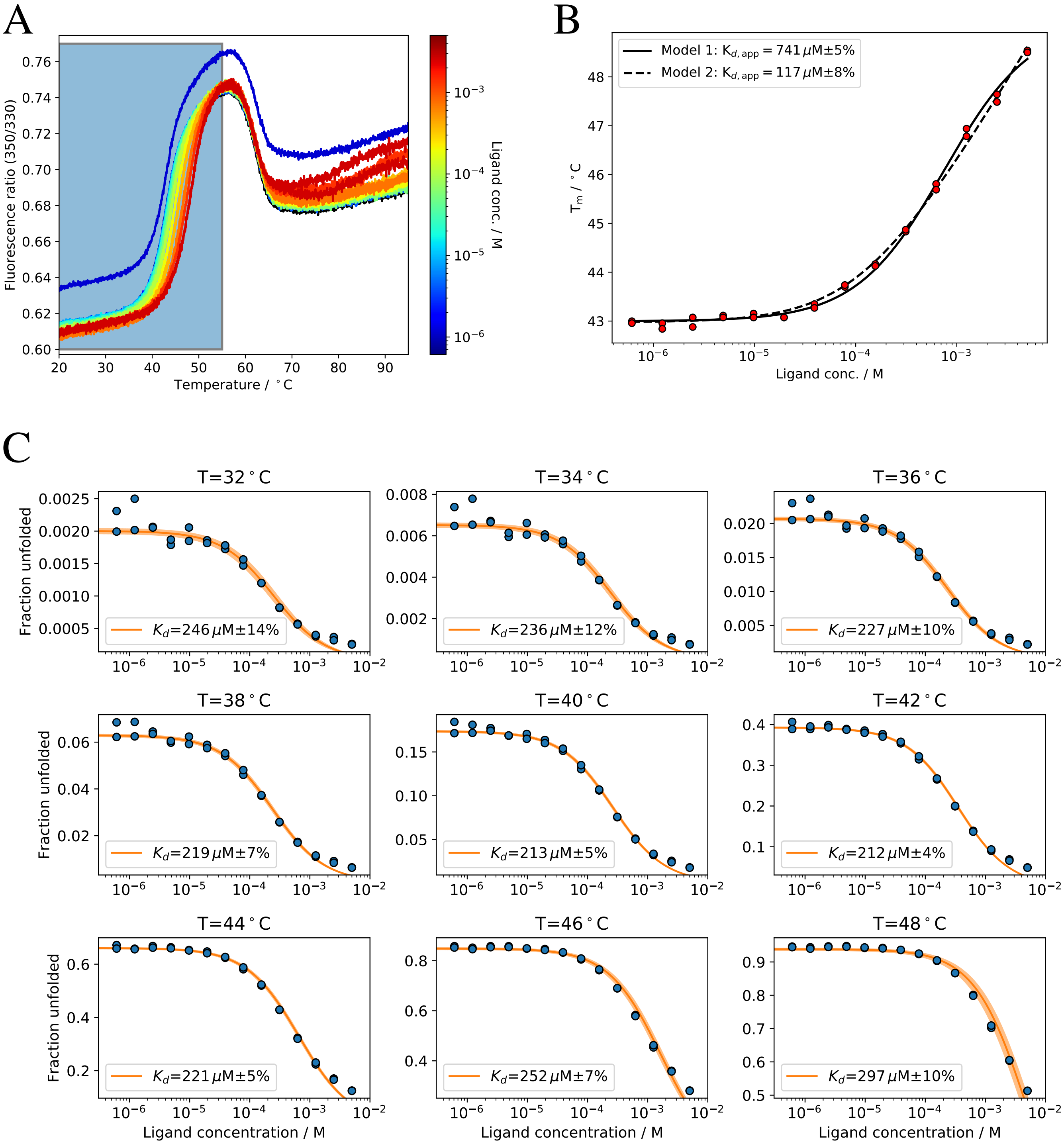

Figure S31. (A) nDSF binding study for Pcs60 K523A/γS-ATP (B) Melting temperature analysis with two different models yields apparent $K_{d,app}$ values. (C) Isothermal analysis for $\Delta C_p = 0$ (bottom).

6 Reversibility tests at different temperatures as fig. 7 (S32)

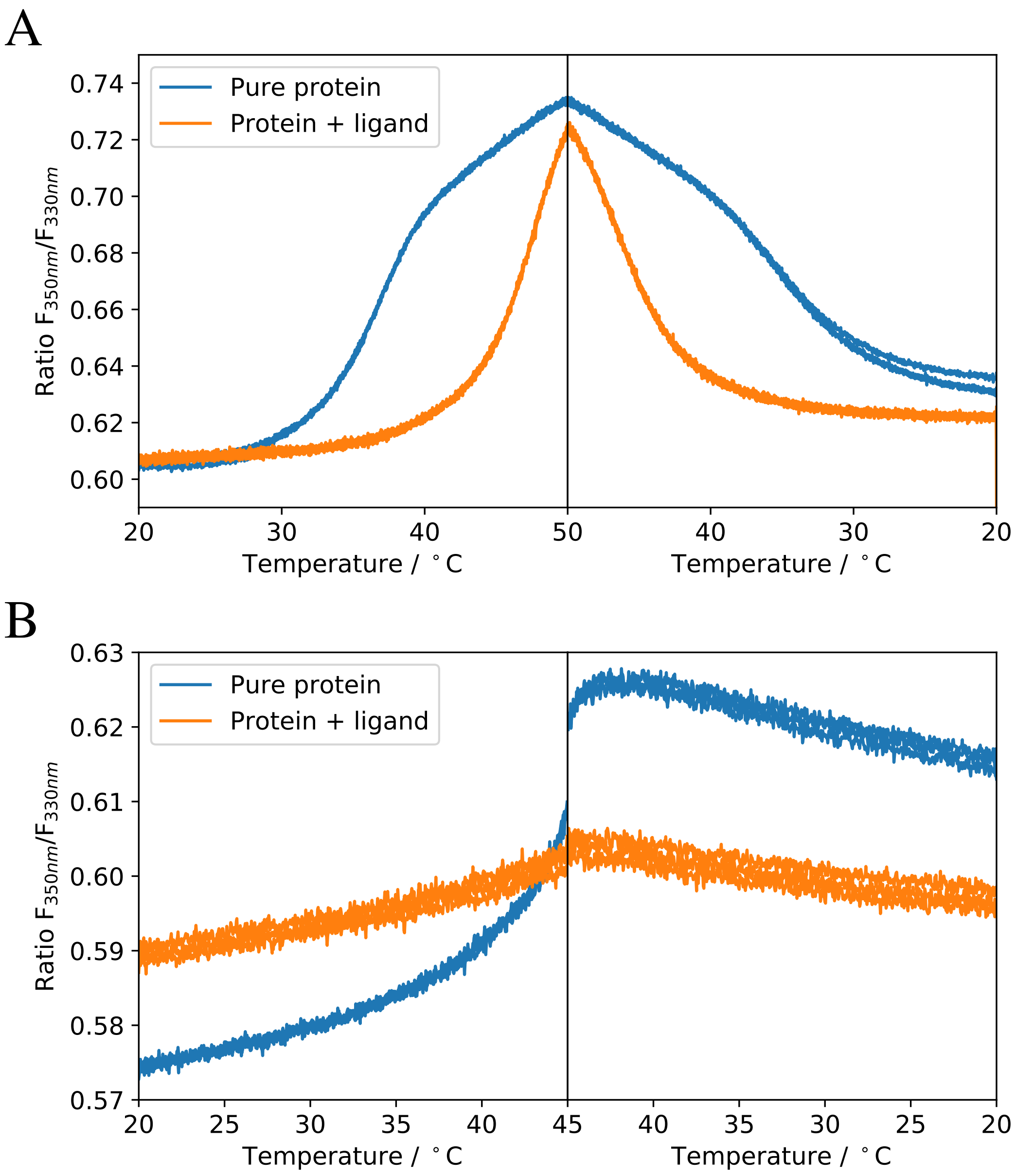

Figure S32. Refolding experiment with Pcs60 (A) and SS1 (B) with a maximum temperature of 50°C. Pcs60 unfolding is still considerably reversible at 50°C whereas SS1 unfolding is irreversible already at 45°C.

7 Heating rate tests with fitted ΔC_p (S33)

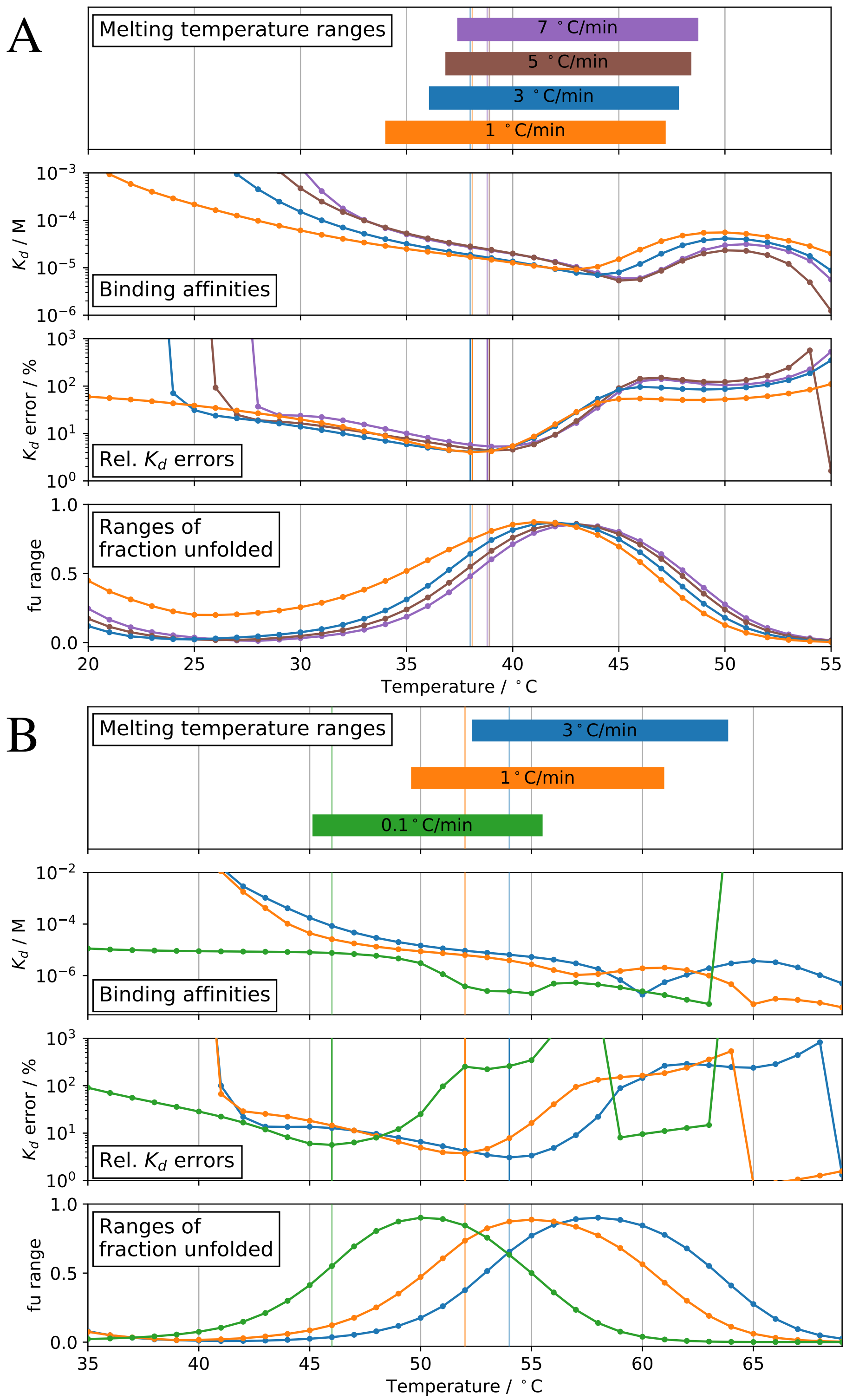

Figure S33. Isothermal analysis at different heating rates for Pcs60/γS-ATP (A) and SS1/ADPR (B). For all datasets, the fitted ΔC_p for a heating rate of 1°C/min was used (cf. main text table 1). The binding affinities were extracted at the temperature with minimum K_d fitting error marked by vertical lines (lines are horizontally shifted for visibility). All temperatures and values are summarized in table 3.

8 nDSF binding study: Protein kinase G (pknG) with inhibitor AX20017

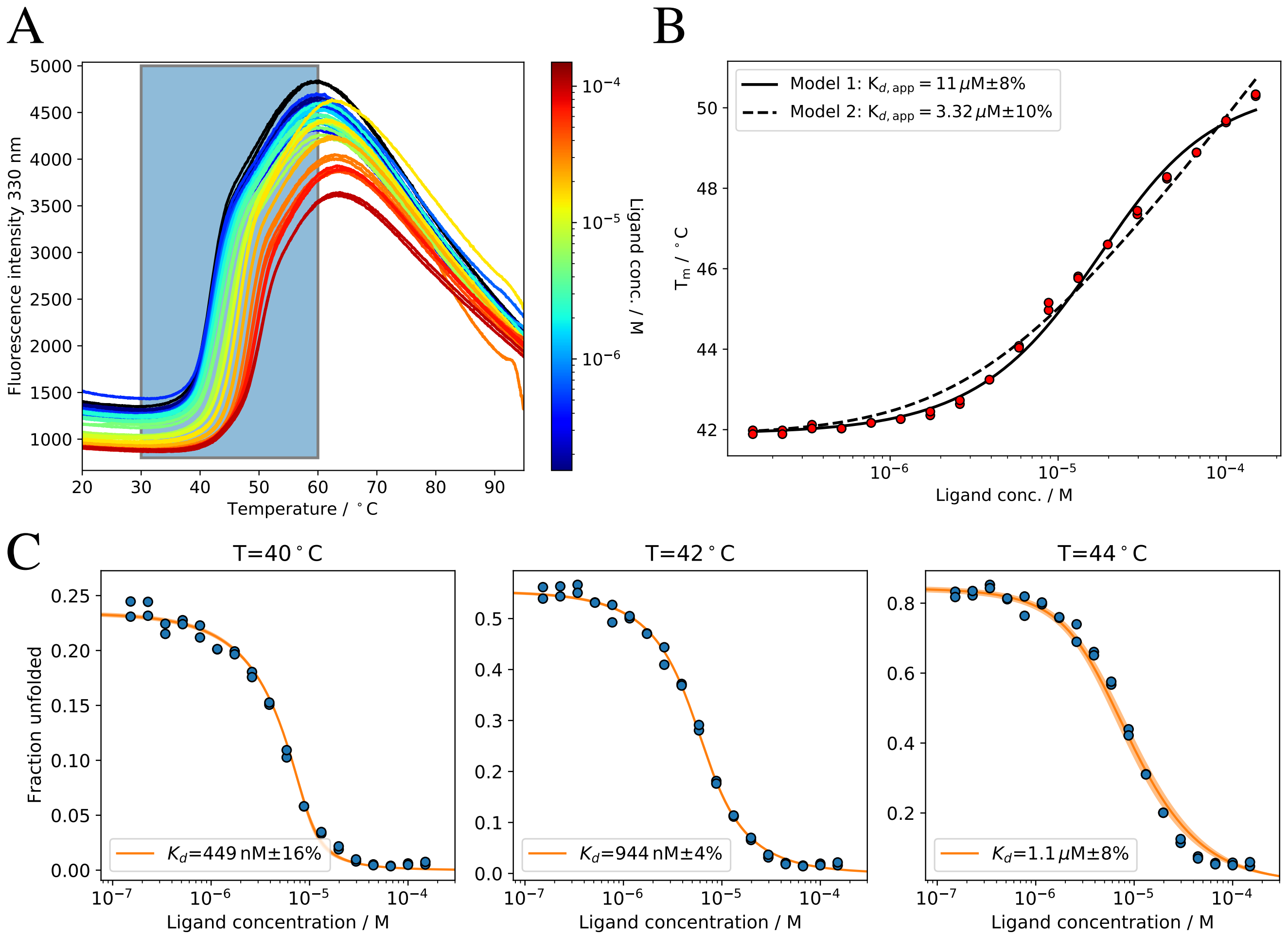

Figure S34. (A) nDSF binding study pknG with inhibitor AX20017[1] at 330 nm. (B) Melting temperature analysis with model 1 and 2. (C) Isothermal analysis of (A) in the marked region at three different temperatures. The K_d from isothermal analysis is in good agreement with the reported IC50 of 390 nM[1].

Fluorescence at 350 nm

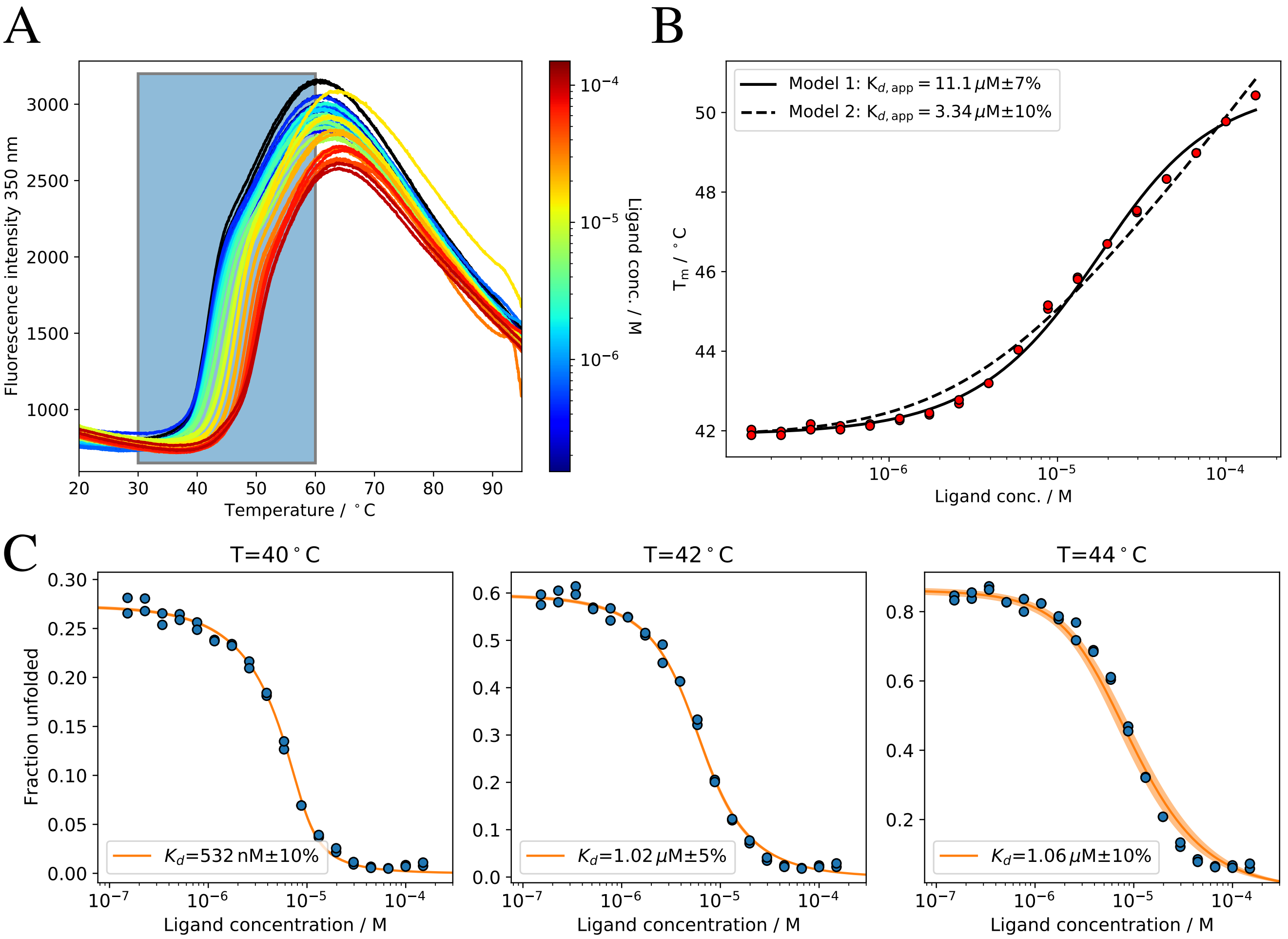

Figure S35. (A) nDSF binding study pknG with inhibitor AX20017[1] at 350 nm. (B) Melting temperature analysis with model 1 and 2. (C) Isothermal analysis of (A) in the marked region at three different temperatures. The K_d from isothermal analysis is in good agreement with the reported IC50 of 390 nM[1].

Fluorescence Ratio

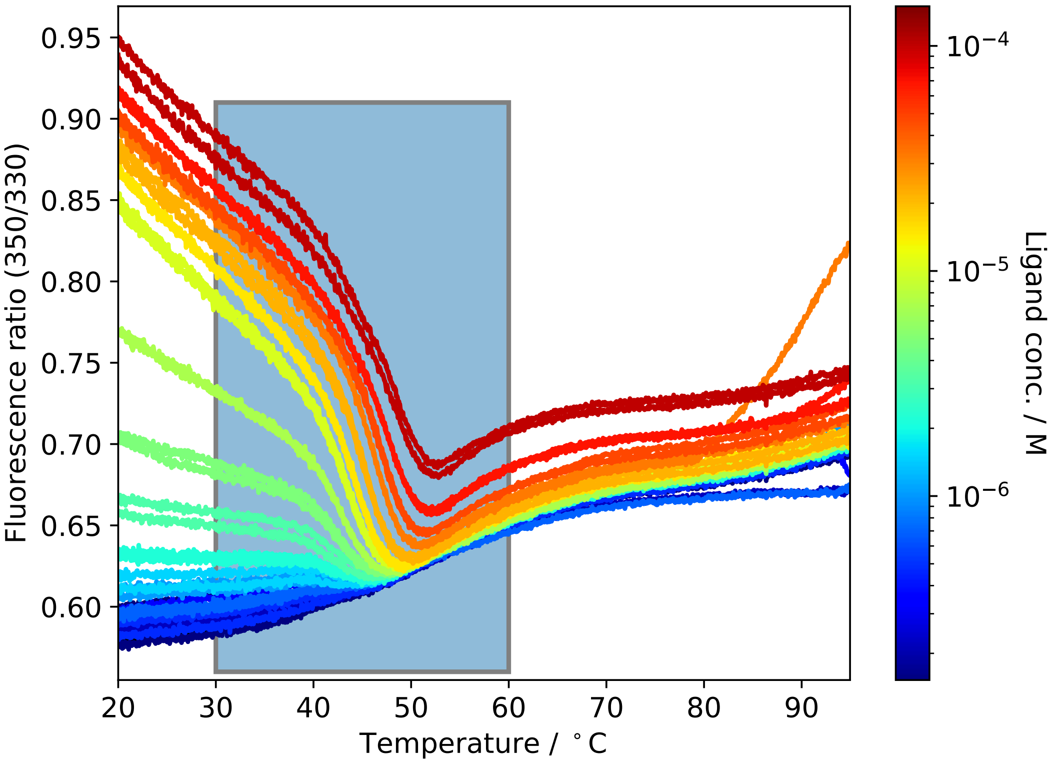

Figure S36. nDSF binding study pknG with inhibitor AX20017[1]. The ratio between of the fluorescence intensities at 350 nm(fig. S35) and 330 nm (fig. S34) could not be used for a melting temperature analysis nor isothermal analysis in this case. This is due to different quenching of the intrinsic protein fluorescence at the two wavelengths for the lower temperatures caused by the ligand which results in a distorted fluorescence ratio.

Methods part: nDSF with pknG

18 dilutions of inhibitor ligand AX20017 starting at 150 μM at a constant pknG concentration of 12 μM were prepared in buffer (25 mM tris-HCl at pH 7.6, 500 mM NaCl, 5% glycerol, 1% DMSO). The dilutions span a ligand concentration range between 150 μM and 152 nM with a dilution factor of 1.5 between each point. In addition to the ligand dilutions, a pure pknG sample in the same buffer was used. The measurements were run as duplicate (in total 38 temperature curves). The LED laser power for the experiments was 100%.

9 Comparison between nDSF and Thermofluor for Pcs60/γS-ATP

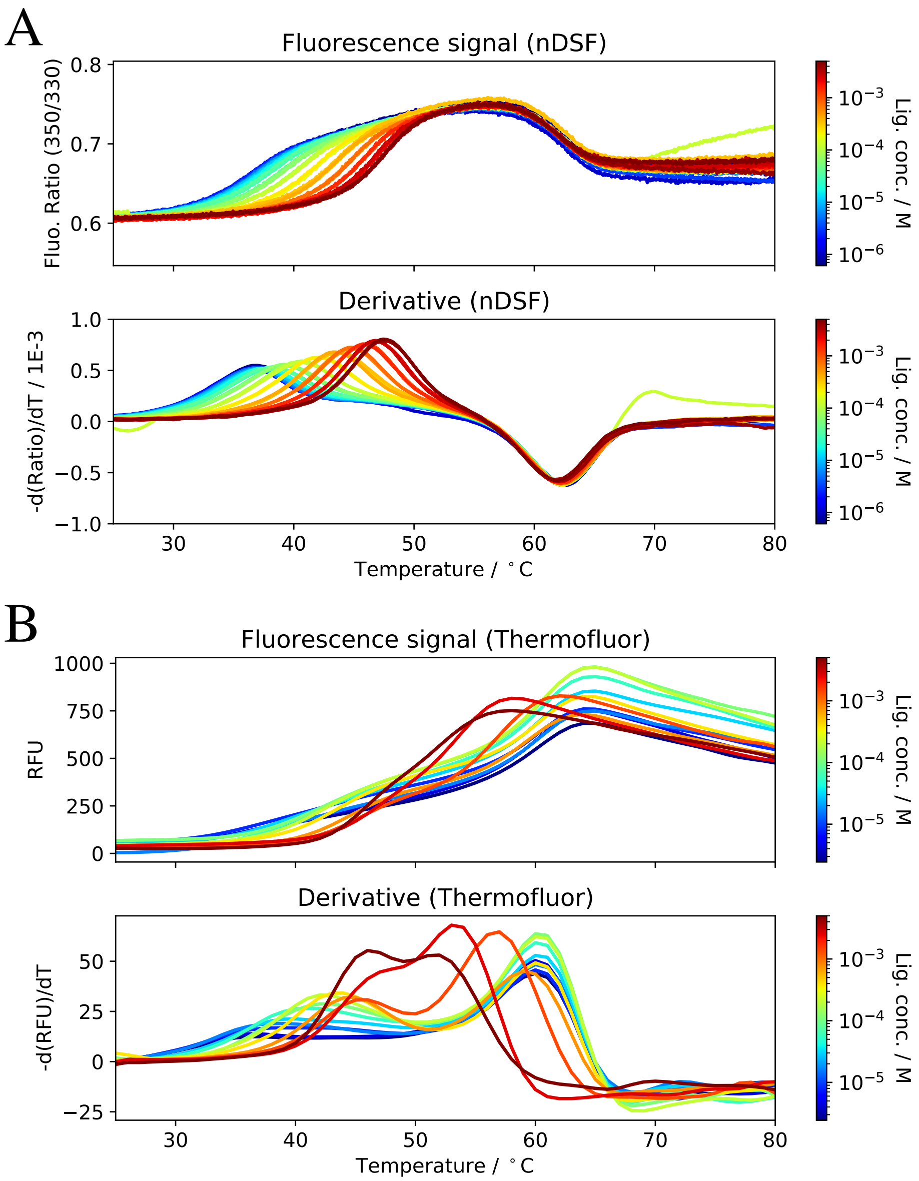

Figure S37. (A) nDSF binding study between Pcs60 and γS-ATP. For the nDSF data, two well separated transitions can be identified around 40 and 60 °C (maximum and minimum of derivatives). The first transition reports on ligand binding. (B) The thermofluor binding study using the dye SYPRO Orange also shows two transitions. However, the first transition is dominated by the second, which renders isothermal analysis more difficult for this protein system.

Methods part: Thermofluor

The Thermofluor measurement shown in fig. S37 was done as described by Boivin *et al.*[2]. Briefly, 2 μl of 20μM Pcs60 and 2 μl of 64x SYPRO orange (Invitrogen) were added to 21μl of γS-ATP ligand solutions (serial dilution). The final concentrations of the ligand are between 5 mM and 2.4 μM (12 dilutions). The final concentration of the protein is 1.6 μM for each sample (including a control without ligand). Thermofluor measurements were performed with an iQ5 PCR detection system (Bio-Rad) using a 96 well plate PCR plate (Greiner Bio One).

10 Fitting procedure of the isothermal analysis

Isothermal analysis consists of three steps:

1. Fitting of thermal curves:
   The observed unfolding enthalpy $\Delta H_{T_m,Obs}$ and the observed melting temperature $T_{m,Obs}$ are fitted from the fluorescence signal versus the temperature T. This is done for each experimental curve (i.e. at each initial ligand concentration $[L]_0$).

2. Slicing of the fraction unfolded along T_{iso}:
   From all $f_{u,[L]_0}(T)$, the fraction unfolded $f_{u,T_{iso}}([L]_0)$ at a chosen temperature T_{iso}, which is typically close to the melting temperature of the protein, is extracted for each initial ligand concentration $[L]_0$.

3. Fitting of the dissociation constant $K_{d,T_{iso}}$:
   This fraction unfolded $f_{u,T_{iso}}([L]_0)$ versus $[L]_0$ is then used to fit the $K_{d,T_{iso}}$ and the unfolding equilibrium constant $K_{U,T_{iso}}$), both at the temperature T_{iso}.

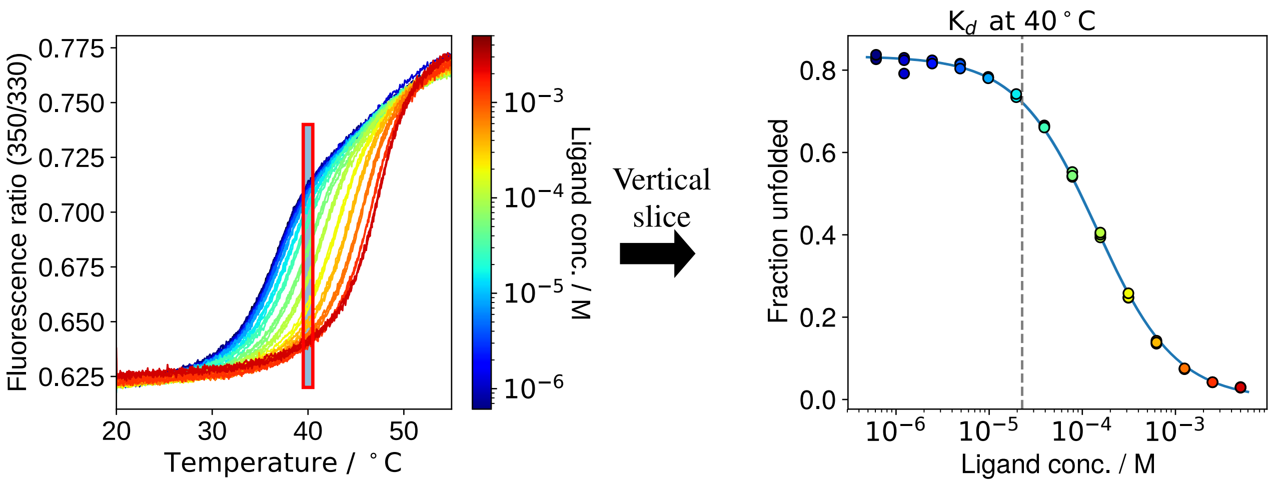

Figure S38. Simplified graphical representation of isothermal analysis.

Step 1. Fitting of thermal curves

For fitting the thermal unfolding curves $Fluo(T)$, we use a reversible two-state folding model between the folded (F) and unfolded protein (U) and the corresponding equilibrium constant K_U which is coupled to a 1:1 ligand (L) binding reaction with the dissociation constant K_d:

$$\mathrm{FL} \underset{}{\overset{\mathrm{K}_d}{\rightleftharpoons}} \mathrm{F} + \mathrm{L} \underset{}{\overset{\mathrm{K_U}}{\rightleftharpoons}} \mathrm{U} + \mathrm{L} \tag{1}$$

The unfolding equilibrium constant is defined as

$$K_U = \frac{[U]}{[F]} \tag{2}$$

with $[U]$ and $[F]$ being the concentrations of the unfolded and folded protein.

We assume that the fluoresence thermal shift assay discriminates between the unfolded species U and the sum of folded species F'. The latter includes both the folded protein F and the protein/ligand complex FL. This approximation is equivalent to assuming the same signal for F and FL. The total fluorescence signal $Fluo(T)$ can then be expressed using the following model:

$$Fluo(T) = f_u(T) * (m_u * T + b_u) + (1 - f_u(T)) * (m_{f'} * T + b_{f'}) \tag{3}$$

with $m_{u/f'}$ being the slopes and $b_{u/f'}$ being the intercepts of the unfolded/folded protein signal, respectively. The fraction unfolded $f_u(T)$ is defined as the ratio between the concentration of the unfolded protein U and the total protein concentration $[P]_0$:

$$f_u(T) = \frac{[U]}{[P]_0} = \frac{[U]}{[U] + [F] + [FL]} \tag{4}$$

In practice, not $f_u(T)$ but the observed unfolding enthalpy $\Delta_{T_m,Obs}$ and the observed melting temperature $T_{m,Obs}$ are fitted for each experimental curve using the following relations:

$$f_u(T) = \frac{K_{U,Obs}(T)}{K_{U,Obs}(T)+1} \tag{5}$$

$$K_{U,Obs}(T) = \frac{[U]}{[F]+[FL]} = e^{-\frac{\Delta G_{U,Obs}(T)}{RT}} \tag{6}$$

$$\Delta G_{U,obs}(T) = \Delta H_{T_m,Obs} * \left(1 - \frac{T}{T_{m,Obs}}\right) - \Delta C_p * \left(T_{m,Obs} - T + ln\left(\frac{T}{T_{m,Obs}}\right)\right) \tag{7}$$

Here, $[U]$, $[F]$ and $[FL]$ are the equilibrium concentrations of the unfolded, folded and complexed protein. $\Delta G_{U,Obs}(T)$ is the apparent Gibbs energy of unfolding and ΔC_p is the change in heat capacity upon unfolding.

When using our webserver, there are three options for the fitting:

1. Local fit of each thermal curve with a fixed change of heat capacity ΔC_p (all six parameters in eq. 3 are fitted individually for each thermal curve)
2. Global fit of the slopes $m_{u/f}$ for all thermal curves (the other parameters are fitted locally)
3. Global fit of $m_{u/f}$ and ΔC_p (the other parameters are fitted locally)

Step 2: Slicing of the fraction unfolded

The parameters $\Delta H_{T_m,Obs}$ and $T_{m,Obs}$ at each initial ligand concentration $[L]_0$ are then used to obtain the fraction unfolded at a selected, fixed temperature T_{iso} using equations 5–7. This way, the fraction unfolded $f_{u,T_{iso}}([L]_0)$ at T_{iso} versus the initial ligand concentrations $[L]_0$ is obtained.

Step 3: Fitting the dissociation constant $\mathbf{K_d}$

So far, ligand binding of the folded protein was not explicitly included in the model by using the dissociation constant K_d:

$$K_d = \frac{[F]*[L]}{[FL]} \tag{8}$$

Still, binding of the ligand was already indirectly included by the dependence of $\Delta H_{T_m,Obs}$ and $T_{m,Obs}$ versus the initial ligand concentration $[L]_0$. Without binding of the ligand, these values and therefore also $f_{u,T_{iso}}([L]_0)$ would be constant. However, if binding of the ligand causes a stabilization of the protein, a decrease of the fraction unfolded with increasing $[L]_0$ is observed.

Inserting equations 2 and 8 into 4 results in an expression for the fraction unfolded that now contains the dissociation constant K_d

$$f_{u,T_{iso}}([L]_{free}) = \frac{K_{U,T_{iso}}}{K_{U,T_{iso}} + \frac{[L]_{free}}{K_{d,T_{iso}}} + 1} \tag{9}$$

The depletion of the free ligand by binding to the protein is taken into account by using the equilibrium concentration of the free ligand $[L]_{free}$ instead of the initial ligand concentration $[L]_0$:

$$[L]_{free} = [L]_0 - [FL] \tag{10}$$

The complex concentration $[FL]$ can be expressed as a function of the initial protein concentration $[P]_0$, the initial ligand concentration $[L]_0$, the dissociation constant K_d and the unfolding equilibrium K_u at T_{iso} using the law of mass action 8 and by expressing $[F]_0$ with the total protein concentration $[P]_0$ using the relation $[F]_0 = [P]_0/(K_U+1)$:

$$K_d = \frac{\left(\frac{[P]_0}{K_U+1} - [FL]\right) * ([L]_0 - [FL])}{[FL]} \tag{11}$$

Solving the respective quadratic equation for the concentration of the complex $[FL]$ yields

$$[FL] = \frac{[L]_0 + [P]_0/(K_U+1) + K_d}{2} - \sqrt{\frac{([L]_0 + [P]_0/(K_U+1) + K_d)^2}{4} - \frac{[L]_0[P]_0}{K_U+1}} \tag{12}$$

Combining equations 9, 10 and 12 results in a function $f_{u,T_{iso},[P]_0}([L]_0, K_{d,T_{iso}}, K_{u,T_{iso}})$ that can be used to fit $K_{d,T_{iso}}$ (and $K_{d,T_{iso}}$) from the calculated $f_{u,T_{iso}}$ versus $[L]_0$ obtained from step 2.

References

1. Walburger, A. Protein kinase g from pathogenic mycobacteria promotes survival within macrophages. *Science* **304**, 1800–1804, DOI: 10.1126/science.1099384 (2004).

2. Boivin, S., Kozak, S. & Meijers, R. Optimization of protein purification and characterization using thermofluor screens. *Prot. Expr. Purif.* **91**, 192–206, DOI: 10.1016/j.pep.2013.08.002 (2013).
